# Design, Synthesis, and Biological Evaluation of Novel N-Acylhydrazone Bond Linked Heterobivalent β-Carbolines as Potential Anticancer Agents

**DOI:** 10.3390/molecules24162950

**Published:** 2019-08-14

**Authors:** Xiaofei Chen, Liang Guo, Qin Ma, Wei Chen, Wenxi Fan, Jie Zhang

**Affiliations:** 1 School of Chemistry and Chemical Engineering, Key Laboratory for Green Processing of Chemical Engineering of Xinjiang Bingtuan, Shihezi University, Shihezi 832003, China; 2 Xinjiang Huashidan Pharmaceutical Research Co. Ltd., 175 He Nan East Road, Urumqi 830011, China

**Keywords:** asymmetric dimeric β-carboline, acylhydrazone group, cytotoxic, antitumor, structure–activity relationship

## Abstract

Utilizing a pharmacophore hybridization approach, we have designed and synthesized a novel series of 28 new heterobivalent β-carbolines. The in vitro cytotoxic potential of each compound was evaluated against the five cancer cell lines (LLC, BGC-823, CT-26, Bel-7402, and MCF-7) of different origin—murine and human, with the aim of determining the potency and selectivity of the compounds. Compound **8z** showed antitumor activities with half-maximal inhibitory concentration (IC_50_) values of 9.9 ± 0.9, 8.6 ± 1.4, 6.2 ± 2.5, 9.9 ± 0.5, and 5.7 ± 1.2 µM against the tested five cancer cell lines. Moreover, the effect of compound **8z** on the angiogenesis process was investigated using a chicken chorioallantoic membrane (CAM) in vivo model. At a concentration of 5 μM, compound **8z** showed a positive effect on angiogenesis. The results of this study contribute to the further elucidation of the biological regulatory role of heterobivalent β-carbolines and provide helpful information on the development of vascular targeting antitumor drugs.

## 1. Introduction

Cancer remains a leading cause of death in developed and developing countries, although much significant progress has been achieved recently [1]. Cancer resistance to therapy is becoming a common phenomenon that threatens the current strategies against this disease. For that reason, we need to discover new anticancer agents. One of the successful and effective methods for the discovery of new anticancer drugs from natural products is synthesis of novel compounds through chemical structural modifications on the basis of leading compounds.

β-Carbolines are a large group of heterocyclic compounds with a 9*H*-pyrido[3,4-*b*]indole structural unit. They compose a class of alkaloids that are widely distributed in nature, including plants, foodstuffs, marine creatures, insects, mammals, human tissues, and body fluids [2]. In the last few decades, there have been intense research efforts in the design and development of β-carbolines as a new class of antitumor agents. A large number of β-carboline derivatives have been prepared in search of more potent antitumor agents. The structure–activity relationships (SARs) of these β-carbolines have been extensively investigated [3,4,5,6,7,8,9,10]. Research has indicated that this class of compounds exert their antitumor effects through multiple mechanisms of action, including intercalating into DNA [11,12,13] and inhibiting topoisomerases I and II [14,15], cyclin-dependent kinase (CDK) [16,17], polo-like kinase 1 (PLK1) [18], kinesin-like protein Eg5 [19], and IκB kinases [20].

**Figure 1:**
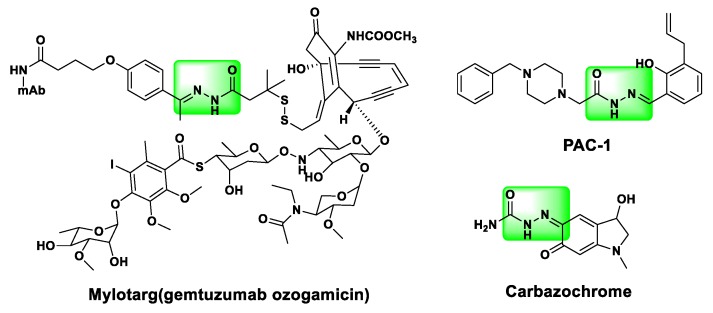
Structures of Mylotarg, PAC-1, and carbazochrome.

Among frequently studied novel bioactive chemical entities, the acylhydrazone scaffold (−CONHN=) has attracted considerable attention for decades due to its broad applications ranging from medicinal agents to agrochemicals to functional materials. Many compounds containing this moiety have been reported, and many reports demonstrate that the introduction of this pharmacophore may have high potential for antitumor activity [21,22,23,24,25,26]. The acylhydrazone moiety is able to act as pharmacophore or auxophore subunit in different pharmaceutic classes, with a variety of action profiles, depending on the other functionalities present in the molecular structure [27]. For example, Mylotarg^TM^ (gemtuzumab ozogamicin; Pfizer) [28] (Figure 1) is a humanized anti-CD33 monoclonal antibody linked covalently to the cytotoxic agent *N*-acetyl gamma calicheamicin. Peterson reported PAC-1 (Figure 1), another N-acylhydrazone small-molecule, induces apoptotic death in cancer cells via the chelation of inhibitory zinc from procaspase-3, which leads to autocatalytic activation and subsequent generation of caspase-3 [29]. Carbazochrome (Figure 1), a semicarbazone-related compound, has been used as a hemostatic agent and is specifically indicated for capillary and parenchymal hemorrhage [30].

Our research group [31,32,33,34] has focused on incorporating substituents into positions 1, 2, 3, 7, and 9 of the β-carboline nucleus as antitumor agents. Structure–activity relationship (SAR) analysis indicated that (1) the β-carboline moiety was associated with their potential antitumor activities, and (2) the introduction of appropriate substituents into positions 1, 3, and 9 of the β-carboline nucleus enhanced their antitumor potencies. Previous research has shown that some antitumor agents when dimerized via an appropriate linker can lead to significantly improved antitumor effects, giving 100- to 500-fold improvement over the corresponding monomers [35,36,37,38].

**Figure 2:**
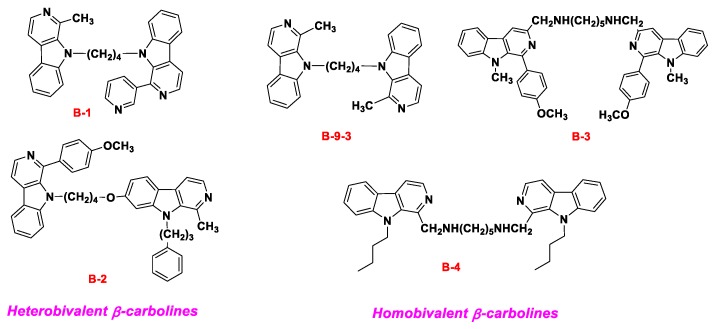
The chemical structure of the representative reported diremic β-carbolines.

So our group reported the synthesis, in vitro evaluation, in vivo efficacies, and SARs of the new homobivalent β-carbolines and heterobivalent β-carbolines with alkyl or alkylamino spacers in positions 1, 3, 7, and 9 of the β-carboline nucleus (Figure 2) [39,40,41,42]. In these homobivalent β-carbolines, 1-Methyl-9-[4-(1-methyl-β-carboline-9-yl)butyl]-β-carboline (B-9-3) [43,44] exhibited potent antitumor activity. The pharmacological mechanisms showed that B-9-3 selectively induces apoptosis of endothelial cells, in part through disruption of VEGF-A/VEGFR2 signaling [45], and also acts on the TGF-β signaling pathway [46]. Compounds B-3 [39] and B-4 [40] exhibited significant angiogenesis inhibitory effects in chicken chorioallantoic membrane (CAM) assay, and the anti-angiogenetic potency was comparable or more potent with the drug Endostar.

**Figure 3:**
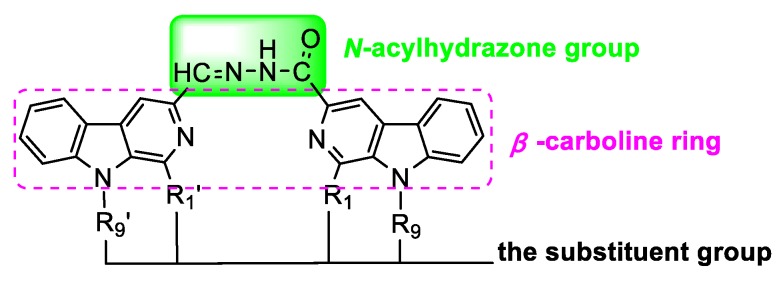
Hybrids of β-carboline and acylhydrazone fragments giving the target heterobivalent β-carbolines.

Continuing our studies to develop effective cytotoxic agents, the objective of this study was to synthesize potential anticancer compounds that are hybrids of β-carboline and acylhydrazone fragments (Figure 3). We have evaluated their cytotoxic activities for the first time, and the study also includes an investigation of the mechanism of action of these compounds for angiogenesis inhibition. These findings as well as our study of the SARs of the new compounds are discussed.

## 2. Results and Discussion

### 2.1. Chemistry

**Scheme 1:**
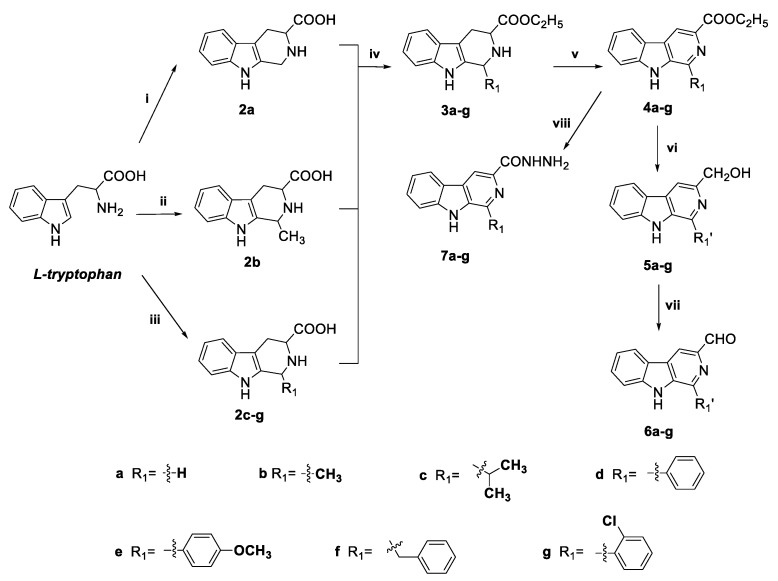
Synthesis of the key intermediates **6a**–**g**, **7a**–**g**. Reagents and conditions: (i) NaOH, H_2_O, formaldehyde, reflux, 3 h; (ii) H_2_SO_4_, H_2_O, acetaldehyde, room temperature, 3 h; (iii) acetic acid, R_1_CHO, reflux, 3 h; (iv) ethanol, SOCl_2_, reflux, 4 h; (v) xylene, S_8_, reflux, 8 h; (vi) THF, LiBH_4_, stirred at RT; (vii) CH_3_CN, MnO_2_, reflux, 2 h. (viii) hydrazine hydrate, ethanol, reflux, 4 h.

**Scheme 2:**
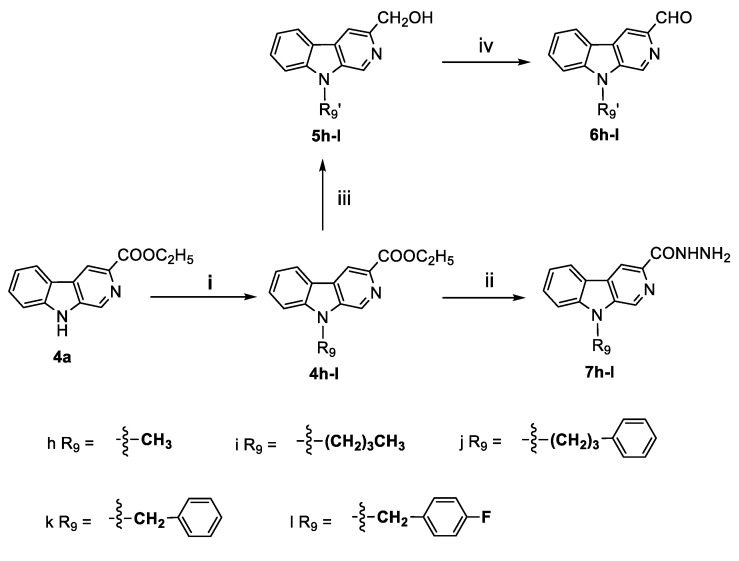
Synthesis of the key intermediates **6h**–**l**, **7h**–**l.** Reagents and conditions: (i) DMF, NaH, alkyl halogenide, stirred at RT; (ii) hydrazine hydrate, ethanol, reflux, 4 h. (iii) THF, LiBH_4_, stirred at RT; (iv) CH_3_CN, MnO_2_, reflux, 2 h.

**Scheme 3:**
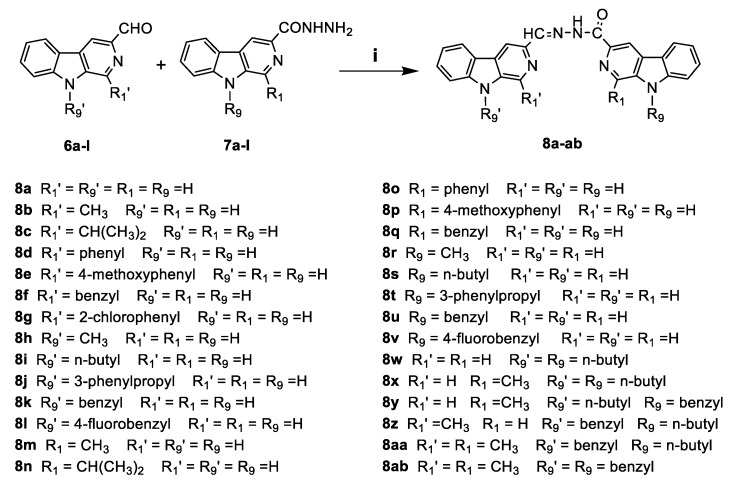
Synthesis of the asymmetric dimeric β-carboline derivatives **8a**–**ab**. Reagents and conditions: (i) methanol, reflux, 4–6 h.

The syntheses of compounds **8a**–**ab** are depicted in Scheme 1, Scheme 2 and Scheme 3. Monovalent β-carbolines **6a**–**l** and **7a**–**l** were synthesized according to previously published methods [39,47,48]. Using L-tryptophan as starting material, the tetrahydro-β-carboline skeleton (**2a**–**g**) was constructed via Pictet–Spengler cyclization. Then, the obtained carboxylic acid **2** reacted with thionyl chloride and ethanol to form ethyl ester **3**, which subsequently reacted with sulfur in xylene to afford compounds **4a**–**g**. Then compounds **4a**–**g** were reduced to their corresponding alcohols by lithium borohydride (LiBH_4_) in dry THF to provide compounds **5a**–**g**, and further oxidized by MnO_2_ in CH_3_CN to afford the key intermediates, the 3-carboxaldehyde derivatives **6a**–**g** [39]. Alternatively, refluxing of compounds **4a**–**g** with 80% hydrazine hydrate in ethanol gave the other key intermediates, hydrazides **7a**–**g** [39] (see Scheme 1).

The *N*^9^-alkylated derivative of compound **4a** was prepared by the action of sodium hydride (NaH) in anhydrous *N*,*N*-dimethylformamide (DMF) followed by the addition of alkyl halide to afford compounds **4h**–**l**, and following this, the intermediates **6h**–**l** and **7h**–**l** were prepared according to the same method for compounds **6a**–**g** and **7a**–**g** (see Scheme 2). Finally, the synthesis of compounds **8a**–**ac** (see Scheme 3) containing the acylhydrazone fragment was accomplished by the condensation of compounds **7a**–**l** with the corresponding aldehydes **6a**–**l**. We obtained the products easily in moderate to good yields when the aldehyde was 1 equiv and when the reactions were carried out in ethanol under reflux conditions. The structures of all compounds were confirmed by ^1^H-NMR, ^13^C-NMR (see Supplementary Materials), and high-resolution mass spectra (HRMS).

### 2.2. MTT Assay of Compounds ***8a**–**8ab***

From the synthetic route mentioned above, we obtained a series of novel heterobivalent β-carbolines. All of the target compounds were assayed for anticancer activity in various cancer cell lines including LLC (Lewis lung carcinoma), BGC-823 (gastric carcinoma), CT-26 (murine colon carcinoma), Bel-7402 (liver carcinoma), and MCF-7 (breast carcinoma), using the MTT method. The half-maximal inhibitory concentration (IC_50_) values for each compound with respect to the five cancer cell lines were calculated, and the results are summarized in Table 1. These values represent the concentrations at which a 50% decrease in cell growth is observed after 72 h of incubation in the presence of the drug compared with control cells treated with DMSO or positive control Cisplatin (DDP) under similar conditions.

For the first experiment, we examined the influence of the substituents in position 1 of the β-carboline core on cytotoxic activities. In order to enhance the range of substituents, we designed 11 novel compounds with methyl and isopropyl substitutions and different patterns of aryl rings substituted by electron withdrawing (Cl) and donating (OCH_3_) groups in the C-1 position of β-carboline. Of these 11 compounds, most of them showed medium or marginal cytotoxic activities in all cell lines. Interestingly, compounds **8b**, **8c**, **8d**, and **8o** were selectively active against BGC823 cells with IC_50_ values lower than 10 µM. In particular, compound **8b** was more potent against BGC823 cells than against the four other cell lines, with potencies in the double-digit µM range. Compounds **8m** (R_1_ = CH_3_) and **8q** (R_1_ = benzyl) were exceptional; they showed no distinct difference between each other, and their IC_50_ values were in the ranges of 13.8–24.7 μM and 13.3–24.5 μM, respectively. Next, we examined the influence of the substituents in position 9 of the β-carboline ring on antiproliferative effects. Compound **8s**, with an *n*-butyl group, was found to be the most potent agent among the heterobivalent β-carbolines, with IC_50_ values of 2.4 ± 0.6 µM (for BGC823) and 3.1 ± 1.2 µM (for CT26). Introduction of benzyl to the R_9_ position on β-carboline yielded compound **8u**, and it demonstrated higher cytotoxic activity than other compounds against all tested tumor cell lines, except for the BGC823 cell line.

Among all these novel molecules, the cytotoxic potencies of most heterobivalent β-carbolines (**8a–ab**) showed no distinct differences, and the IC_50_ values of this class of compounds ranged from 10 to 100 µM. **8a** and **8p** had poor inhibitory activities with IC_50_ values above 50 µM. Compound **8z** exhibited the most potent anticancer activity against the LLC, BGC-823, CT-26, Bel-7402, and MCF-7 cell lines, with IC_50_ values of 9.9 ± 0.9, 8.6 ± 1.4, 6.2 ± 2.5, 9.9 ± 0.5, and 5.7 ± 1.2 µM, respectively.

### 2.3. Inhibition of Angiogenesis in the Chicken Chorioallantoic Membrane (CAM) Assay

**Figure 4:**
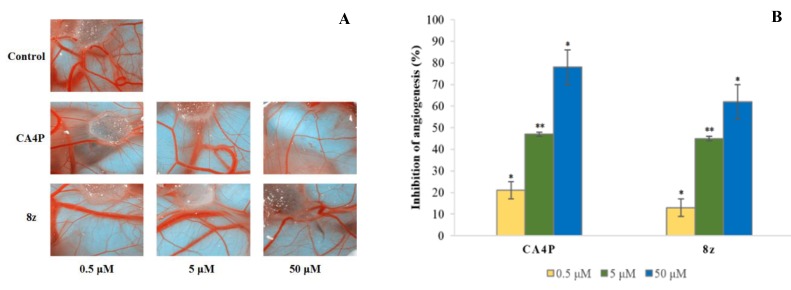
In vivo anti-angiogenic effect of compounds **8z** in CAM assay. (**A**) The representative photos of the experiments. (**B**) The anti-angiogenetic activity of compound **8z** was semi-quantitatively analyzed using Graph Pad Prism 5.0. The data represent the mean values ± SD of at least three independent determinations. (**, *p* < 0.05; *, *p* < 0.5).

The CAM assay was deployed to assess the inhibitory effect of compound **8z** on neovascularization. In this experiment, we used Combretastatin A4 disodium phosphate (CA4P) as a positive control. The inhibitory effects of compound **8z** on angiogenesis in CAM are shown in Figure 4A. At the dose 0.5 μM, the reference anti-angiogenic drug CA4P elicited 21% inhibition of angiogenesis, and compound **8z** did not show a significant anti-angiogenic activity at this concentration (13% inhibition). The anti-angiogenetic activity of compound **8z** was comparable with CA4P in an in vivo CAM assay at the 5 μM level. In this assay, **8z** inhibited blood vessel formation by 45%, compared to 47% inhibition induced by CA4P (*p* < 0.05). At 50 μM, CA4P significantly inhibited blood vessel formation, eliciting 78% inhibition (Figure 4B).

## 3. Materials and Methods

### 3.1. Reagents and General Methods

MTT was obtained from Sigma-Aldrich (Darmstadt, Germany) and Cisplatin from Qilu pharmaceutical (Jinan, China). Other commercially available starting materials and solvents were reagent grade and were purchased from Adamas-beta and used without further purification. Reactions and products were routinely monitored by thin-layer chromatography (TLC) on silica gel F254 plates (Qingdao Haiyang Inc., Qingdao, China). ^1^H-NMR and ^13^C-NMR spectra were recorded at room temperature on a Bruker Avance III HD 400 instrument (Bruker Company, Bremen, Gemany) using tetramethylsilane as the internal reference. Chemical shifts (*δ*) were reported in ppm relative to the residual solvent peak, and the multiplicity of each signal was designated by the following abbreviations—s, singlet; d, doublet; t, triplet; q, quartet; m, multiplet. Coupling constants (*J*) were quoted in Hz. HRMS were recorded on a Bruker ultrafleXtreme MALDI-TOF/TOF-MS and Thermo Scientific LTQ Orbitrap XL (Thermo Fisher Scientific Inc, Waltham, USA). Column chromatography was performed with silica gel (200–300 mesh, Qingdao Haiyang Inc., Qingdao, China).

### 3.2. General Procedure for the Preparation of ***6a**–**lc***

A mixture of compound **5a** (1.98 g, 10 mmol) and activated MnO_2_ (30 mmol) in CH_3_CN (60 mL) was stirred under reflux for 2 h. After completion of the reaction (monitored by TLC), the products were cooled to room temperature and filtered through Celite. The filtrate was passed through silica gel and washed with dichloromethane, and the solvent was removed under reduced pressure. The residue was crystallized from acetone or acetone–petroleum ether to give the corresponding compound **6a**. Products **6b**–**l** were prepared according to the same method as **6a**.

*1-benzyl-β-carboline-3-carbaldehyde* (**6f**): The compound was obtained as a white solid with 81% yield. ^1^H-NMR (400 MHz, CDCl_3_) *δ* 10.24 (s, 1H, CHO), 8.68 (s, 1H, ArH), 8.64 (s, 1H, ArH), 8.14 (d, *J* = 8.0 Hz, 1H, ArH), 7.51 (d, *J* = 8.0 Hz, 1H, ArH), 7.43 (d, *J* = 8.4 Hz, 1H, ArH), 7.35–7.30 (m, 4H, ArH), 7.25–7.23 (m, 2H, ArH), 4.61 (s, 2H, ArCH_2_). ^13^C-NMR (100 MHz, CDCl_3_) *δ* 193.41, 144.21, 143.97, 137.68, 129.04, 129.00, 128.94, 128.81, 128.77, 127.08, 127.01, 121.92, 121.80, 121.20, 114.09, 112.11, 41.56.

*1-(2-chlorophenyl)-β-carboline-3-carbaldehyde* (**6g**): The compound was obtained as a light yellow solid with 72% yield. ^1^H-NMR (400 MHz, CDCl_3_) *δ* 10.28 (s, 1H, CHO), 8.79 (s, 1H, ArH), 8.39 (s, 1H, ArH), 8.25 (d, *J* = 8.0 Hz, 1H, ArH), 7.69 – 7.66 (m, 1H, ArH), 7.64–7.59 (m, 2H, ArH), 7.56–7.49 (m, 3H, ArH), 7.40 (t, *J* = 7.6 Hz, 1H, ArH). ^13^C-NMR (100 MHz, CDCl_3_) *δ* 193.33, 144.43, 141.54, 140.61, 136.39, 136.02, 132.89, 132.03, 130.79, 130.32, 129.56, 129.42, 127.71, 122.22, 122.16, 121.48, 114.17, 111.97.

### 3.3. General Procedure for the Preparation of Compounds ***7a**–**l***

We added 85% hydrazine hydrate (10 mL) to a solution of compound **4a** (2.40 g, 10 mmol) in ethanol (100 mL), and then the mixture was refluxed for 8 h. Following the completion of reaction (as demonstrated by TLC), the resulting mixture was cooled to 5 °C and the precipitate was collected by filtration. The crude product was further purified first by washing with ethanol and then by recrystallization in ethanol to obtain compound **7a** with a yield of 85%. Product **7b**–**l** was prepared according to the same method as **7a**.

*1-isopropyl-β-carboline-3-carbohydrazide* (**7c**): The compound was obtained as a white solid with 91% yield. ^1^H-NMR (400 MHz, DMSO-*d*_6_) *δ* 9.47 (s, 1H, NH), 8.65 (s, 1H, ArH), 8.35 (d, *J* = 7.6 Hz, 1H, ArH), 7.65 (d, *J* = 8.0 Hz, 1H, ArH), 7.60–7.55 (m, 1H, ArH), 7.30–7.26 (m, 1H, ArH), 4.58 (s, 2H, NH_2_), 3.75–3.64 (m, 1H, CH), 1.43 (d, *J* = 6.8 Hz, 6H, CH_3_). ^13^C-NMR (100 MHz, DMSO-*d*_6_) *δ* 164.65, 149.72, 141.16, 138.99, 134.81, 128.67, 128.22, 122.44, 121.92, 120.27, 112.62, 112.19, 31.21, 21.81.

*1-benzyl-β-carboline-3-carbohydrazide* (**7f**): The compound was obtained as a white solid with 94% yield. ^1^H-NMR (400 MHz, DMSO-*d*_6_) *δ* 9.54 (s, 1H, NH), 8.69 (s, 1H, ArH), 8.37 (d, *J* = 8.0 Hz, 1H, ArH), 7.67 (d, *J* = 8.4 Hz, 1H, ArH), 7.63–7.57 (m, 1H, ArH), 7.51 (d, *J* = 7.2 Hz, 2H, ArH), 7.34–7.25 (m, 4H, ArH), 7.20–7.15 (m, 1H, ArH), 4.60 (s, 2H, NH_2_), 4.51 (s, 2H, ArCH_2_). ^13^C-NMR (100 MHz, DMSO-*d*_6_) *δ* 164.43, 143.63, 141.29, 139.36, 139.33, 135.69, 129.36, 129.05, 128.92, 128.82, 128.76, 126.65, 122.60, 121.88, 120.44, 112.68, 112.59, 61.00.

*9-n-butyl-β-carboline-3-carbohydrazide* (**7i**): The compound was obtained as a light yellow solid with 80% yield. ^1^H-NMR (400 MHz, DMSO-*d*_6_) *δ* 9.70 (s, 1H, NH), 9.05 (d, *J* = 1.2 Hz, 1H, ArH), 8.84 (d, *J* = 0.8 Hz, 1H, ArH), 8.44 (d, *J* = 8.0 Hz, 1H, ArH), 7.77 (d, *J* = 8.4 Hz, 1H, ArH), 7.68–7.63 (m, 1H, ArH), 7.36–7.31 (m, 1H, ArH), 4.59–4.54 (m, 4H, NH_2_, –CH_2_CH_2_CH_2_CH_3_), 1.85–1.76 (m, 2H, –CH_2_CH_2_CH_2_CH_3_), 1.35–1.24 (m, 2H, –CH_2_CH_2_CH_2_CH_3_), 0.88 (t, *J* = 7.2 Hz, 3H, –CH_2_CH_2_CH_2_CH_3_). ^13^C-NMR (100 MHz, DMSO-*d*_6_) *δ* 164.37, 141.62, 140.00, 137.58, 131.70, 129.16, 128.14, 122.87, 121.16, 120.55, 114.14, 110.97, 43.05, 31.38, 20.20, 14.14.

*9-(3-phenylpropyl)-β-carboline-3-carbohydrazide* (**7j**): The compound was obtained as a gray powder with 86% yield. ^1^H-NMR (400 MHz, DMSO-*d*_6_) *δ* 9.69 (s, 1H, NH), 9.00 (d, *J* = 1.2 Hz, 1H, ArH), 8.83 (d, *J* = 0.8 Hz, 1H, ArH), 8.44 (d, *J* = 8.0 Hz, 1H, ArH), 7.72 (d, *J* = 8.4 Hz, 1H, ArH), 7.68–7.62 (m, 1H, ArH), 7.37–7.31 (m, 1H, ArH), 7.29–7.22 (m, 2H, ArH), 7.21–7.13 (m, 3H, ArH), 4.60 (t, *J* = 7.2 Hz, 2H, ArCH_2_CH_2_CH_2_), 4.56 (s, 2H, NH_2_), 2.67 (t, *J* = 7.6 Hz, 2H, ArCH_2_CH_2_CH_2_), 2.19–2.10 (m, 2H, ArCH_2_CH_2_CH_2_). ^13^C-NMR (101 MHz, DMSO-*d*_6_) *δ* 164.34, 141.59, 141.55, 140.08, 137.52, 131.62, 129.20, 128.80, 128.58, 128.24, 126.37, 122.92, 121.22, 120.62, 114.16, 110.89, 43.03, 32.85, 30.84.

*9-(4-fluorobenzyl)-β-carboline-3-carbohydrazide* (**7l**): The compound was obtained as a white solid with 96% yield. ^1^H-NMR (400 MHz, DMSO-*d*_6_) *δ* 9.76 (s, 1H, NH), 9.11 (d, *J* = 1.2 Hz, 1H, ArH), 8.89 (d, *J* = 1.2 Hz, 1H, ArH), 8.48 (d, *J* = 7.6 Hz, 1H, ArH), 7.83 (d, *J* = 8.4 Hz, 1H, ArH), 7.68–7.63 (m, 1H, ArH), 7.39–7.34 (m, 1H, ArH), 7.33–7.29 (m, 2H, ArH), 7.17–7.11 (m, 2H, ArH), 5.85 (s, 2H, ArCH_2_), 4.61 (s, 2H, NH_2_). ^13^C-NMR (100 MHz, DMSO-*d*_6_) *δ* 164.30, 162.52 (d, *J* = 242.1 Hz), 141.65, 140.50, 137.58, 133.86 (d, *J* = 3.2 Hz), 131.90, 129.51, 129.43, 129.39, 128.59, 122.98, 121.17 (d, *J* = 46.3 Hz), 115.99 (d, *J* = 21.4 Hz), 114.25, 111.18, 45.88.

### 3.4. General Procedure for the Preparation of Heterobivalent β-Carbolines ***8a**–**ab***

We added β-carboline-3-carbaldehyde **6a**–**l** (1 mmol) to a solution of β-carboline-3-carbohydrazide **7a**–**l** (1 mmol) in EtOH (50 mL), and then the reaction mixture was refluxed for 5 h. The solution was allowed to cool to room temperature. Then, the precipitates formed and were filtered, and the crude product was recrystallized with ethanol to afford compounds **8a**–**ab**.

*N′-((9H-pyrido[3,4-b]indol-3-yl)methylene)-9H-pyrido[3,4-b]indole-3-carbohydrazide* (**8a**): The compound was obtained as a yellow solid with 93% yield. ^1^H-NMR (400 MHz, DMSO-*d*_6_) *δ* 12.13 (s, 1H, NH), 12.11 (s, 1H, NH), 9.28 (s, 1H, ArH), 9.16 (d, *J* = 0.8 Hz, 1H, ArH), 9.02 (s, 1H, ArH), 8.63 (s, 1H, ArH), 8.46 (d, *J* = 8.0 Hz, 1H, CH), 8.34 (d, *J* = 8.0 Hz, 1H, ArH), 7.80 (s, 1H, ArH), 7.73 (d, *J* = 8.0 Hz, 1H, ArH), 7.70 (d, *J* = 8.0 Hz, 1H, ArH), 7.68–7.61 (m, 2H, ArH), 7.39–7.32 (m, 2H, ArH). ^13^C-NMR (100 MHz, DMSO-*d*_6_) *δ* 162.68, 142.36, 141.65, 141.50, 140.77, 139.78, 137.85, 135.54, 133.55, 133.31, 129.49, 129.16, 128.80, 128.66, 122.81, 122.53, 121.47, 121.24, 120.74, 120.59, 118.99, 115.46, 112.99, 112.78. HRMS *m*/*z* calculated for C_24_H_17_N_6_O^+^ (M + H)^+^: 405.1458; found 405.1459.

*N′-((1-methyl-9H-pyrido[3,4-b]indol-3-yl)methylene)-9H-pyrido[3,4-b]indole-3-carbohydrazide* (**8b**): The compound was obtained as a yellow solid with 84% yield. ^1^H-NMR (400 MHz, DMSO-*d*_6_) *δ* 12.15 (s, 1H, NH), 12.02 (s, 1H, NH), 9.08 (d, *J* = 0.8 Hz, 1H, ArH), 9.03 (s, 1H, ArH), 8.46 (d, *J* = 8.0 Hz, 1H, CH), 8.44 (s, 1H, ArH), 8.29 (d, *J* = 8.0 Hz, 1H, ArH), 7.74 (s, 1H, ArH), 7.73–7.69 (m, 2H, ArH), 7.66–7.60 (m, 2H, ArH), 7.34 (t, *J* = 7.6 Hz, 2H, ArH), 3.20 (s, 3H, CH_3_). ^13^C-NMR (100 MHz, DMSO-*d*_6_) *δ* 162.54, 141.96, 141.86, 141.48, 141.36, 140.92, 139.96, 137.89, 134.27, 133.02, 129.17, 129.13, 128.75, 128.02, 122.80, 122.45, 121.71, 121.48, 120.66, 120.60, 117.15, 115.48, 112.88, 112.81, 20.96. HRMS *m*/*z* calculated for C_25_H_19_N_6_O^+^ (M + H)^+^: 419.1615; found 419.1620.

*N′-((1-isopropyl-9H-pyrido[3,4-b]indol-3-yl)methylene)-9H-pyrido[3,4-b]indole-3-carbohydrazide* (**8c**): The compound was obtained as a yellow solid with 89% yield. ^1^H-NMR (400 MHz, DMSO-*d*_6_) *δ* 15.64 (s, 1H, CONH), 12.10 (s, 1H, NH), 12.05 (s, 1H, NH), 9.07 (s, 1H, ArH), 9.02 (s, 1H, ArH), 8.49 (d, *J* = 8.0 Hz, 1H, CH), 8.46 (s, 1H, ArH), 8.30 (d, *J* = 8.0 Hz, 1H, ArH), 7.78 (s, 1H, ArH), 7.75–7.69 (m, 2H, ArH), 7.67–7.62 (m, 2H, ArH), 7.38–7.33 (m, 2H, ArH), 4.02–3.91 (m, 1H, CH(CH_3_)_2_), 1.76 (d, *J* = 7.2 Hz, 6H, CH(CH_3_)_2_). ^13^C-NMR (100 MHz, DMSO-*d*_6_) *δ* 162.76, 151.03, 142.16, 141.51, 141.38, 141.17, 139.54, 138.00, 132.90, 132.63, 129.25, 129.20, 128.88, 128.60, 122.87, 122.35, 121.72, 121.45, 120.66, 120.62, 117.59, 115.71, 112.87, 112.82, 31.57, 21.63. HRMS *m*/*z* calculated for C_27_H_23_N_6_O^+^ (M + H)^+^: 447.1928; found 447.1932.

*N′-((1-phenyl-9H-pyrido[3,4-b]indol-3-yl)methylene)-9H-pyrido[3,4-b]indole-3-carbohydrazide* (**8d**): The compound was obtained as a yellow solid with 96% yield. ^1^H-NMR (400 MHz, DMSO-*d*_6_) *δ* 15.58 (s, 1H, CONH), 12.06 (s, 1H, NH), 11.95 (s, 1H, NH), 8.99 (s, 1H, ArH), 8.65 (s, 1H, ArH), 8.44 (d, *J* = 8.4 Hz, 2H, CH, ArH), 8.35 (d, *J* = 8.0 Hz, 1H, ArH), 8.26–8.23 (m, 2H, ArH), 7.85 (s, 1H, ArH), 7.82–7.73 (m, 4H, ArH), 7.69–7.59 (m, 3H, ArH), 7.41–7.35 (m, 1H, ArH), 7.35–7.29 (m, 1H, ArH). ^13^C-NMR (100 MHz, DMSO-*d*_6_) *δ* 162.83, 142.56, 142.51, 142.15, 141.43, 140.85, 139.47, 137.75, 137.64, 133.10, 132.78, 130.10, 129.75, 129.47, 129.44, 129.32, 129.16, 128.59, 122.81, 122.36, 121.46, 121.41, 120.91, 120.57, 118.56, 115.52, 113.36, 112.74. HRMS *m*/*z* calculated for C_30_H_21_N_6_O^+^ (M + H)^+^: 481.1771; found 481.1777.

*N′-((1-(4-methoxyphenyl)-9H-pyrido[3,4-b]indol-3-yl)methylene)-9H-pyrido[3,4-b]indole-3-carbohydrazide* (**8e**): The compound was obtained as a yellow solid with 91% yield. ^1^H-NMR (400 MHz, DMSO-*d*_6_) *δ* 15.53 (s, 1H, CONH), 12.12 (s, 1H, NH), 11.90 (s, 1H, NH), 9.01 (s, 1H, ArH), 8.59–8.57 (m, 2H, ArH), 8.45 (d, *J* = 7.6 Hz, 1H, CH), 8.33 (d, *J* = 7.6 Hz, 1H, ArH), 8.23 (d, *J* = 8.8 Hz, 2H, ArH), 7.83 (s, 1H, ArH), 7.74 (d, *J* = 8.4 Hz, 1H, ArH), 7.70 (d, *J* = 8.4 Hz, 1H, ArH), 7.68–7.60 (m, 2H, ArH), 7.39–7.31 (m, 4H, ArH), 4.02 (s, 3H, OCH_3_). ^13^C-NMR (100 MHz, DMSO-*d*_6_) *δ* 162.86, 160.53, 142.53, 142.45, 142.07, 141.48, 140.97, 139.51, 137.76, 132.92, 132.80, 131.10, 130.00, 129.88, 129.30, 129.18, 128.65, 122.82, 122.29, 121.50, 121.42, 120.84, 120.58, 118.19, 115.63, 114.72, 113.36, 112.75, 56.05. HRMS *m*/*z* calculated for C_31_H_23_N_6_O_2_^+^ (M + H)^+^: 511.1877; found 511.1878.

*N′-((1-benzyl-9H-pyrido[3,4-b]indol-3-yl)methylene)-9H-pyrido[3,4-b]indole-3-carbohydrazide* (**8f)**: The compound was obtained as a yellow solid with 82% yield. ^1^H-NMR (400 MHz, DMSO-*d*_6_) *δ* 12.19 (s, 1H, NH), 11.95 (s, 1H, NH), 9.06 (s, 1H, ArH), 8.88 (s, 1H, ArH), 8.50 (s, 1H, ArH), 8.47 (d, *J* = 7.6 Hz, 1H, CH), 8.29 (d, *J* = 7.6 Hz, 1H, ArH), 7.78–7.72 (m, 4H, ArH), 7.69–7.59 (m, 4H, ArH), 7.37–7.32 (m, 2H, ArH), 7.23 (t, *J* = 7.6 Hz, 2H, ArH), 7.13 (t, *J* = 7.2 Hz, 1H, ArH), 4.90 (s, 2H, ArCH_2_). ^13^C-NMR (100 MHz, DMSO-*d*_6_) *δ* 162.61, 144.08, 142.15, 141.48, 141.44, 140.83, 139.92, 139.26, 137.88, 133.58, 132.82, 131.50129.39, 129.23, 129.11, 128.95, 128.82, 126.84, 122.85, 122.44, 121.68, 121.45, 120.80, 120.64, 117.69, 115.64, 112.96, 112.78. HRMS *m*/*z* calculated for C_31_H_23_N_6_O^+^ (M + H)^+^: 495.1928; found 495.1932.

*N′-((1-(2-chlorophenyl)-9H-pyrido[3,4-b]indol-3-yl)methylene)-9H-pyrido[3,4-b]indole-3-carbohydrazide* (**8g**): The compound was obtained as a yellow solid with 81% yield. ^1^H-NMR (400 MHz, DMSO-*d*_6_) *δ* 12.04 (s, 1H, NH), 11.85 (s, 1H, NH), 8.92 (s, 1H, ArH), 8.70 (s, 1H, ArH), 8.41 (d, *J* = 8.0 Hz, 1H, CH), 8.37 (d, *J* = 8.0 Hz, 1H, ArH), 7.96 (d, *J* = 1.2 Hz, 1H, ArH), 7.90–7.85 (m, 3H, ArH), 7.84–7.79 (m, 1H, ArH), 7.77–7.72 (m, 1H, ArH), 7.70–7.55 (m, 5H, ArH), 7.40–7.35 (m, 1H, ArH), 7.31 (t, *J* = 7.2 Hz, 1H, ArH). ^13^C-NMR (100 MHz, DMSO-*d*_6_) *δ* 162.71, 142.01, 141.37, 140.58, 140.53, 139.50, 137.61, 136.59, 133.76, 133.32, 132.75, 132.47, 131.09, 130.30, 129.58, 129.55, 129.09, 128.46, 127.95, 122.75, 122.50, 121.40, 121.38, 120.86, 120.52, 118.77, 115.25, 113.09, 112.70. HRMS *m*/*z* calculated for C_30_H_20_ClN_6_O^+^ (M + H)^+^: 515.1382; found 515.1386.

*N′-((9-methyl-9H-pyrido[3,4-b]indol-3-yl)methylene)-9H-pyrido[3,4-b]indole-3-carbohydrazide* (**8h**): The compound was obtained as an ivory solid with 84% yield. ^1^H-NMR (400 MHz, DMSO-*d*_6_) *δ* 12.14 (s, 1H, NH), 9.45 (s, 1H, ArH), 9.15 (s, 1H, ArH), 9.01 (s, 1H, ArH), 8.64 (s, 1H, ArH), 8.46 (d, *J* = 8.0 Hz, 1H, CH), 8.36 (d, *J* = 8.0 Hz, 1H, ArH), 7.83 (d, *J* = 8.4 Hz, 1H, ArH), 7.80 (s, 1H, ArH), 7.74 (d, *J* = 7.6 Hz, 1H, ArH), 7.70 (d, *J* = 8.4 Hz, 1H, ArH), 7.63 (t, *J* = 7.6 Hz, 1H, ArH), 7.41 (t, *J* = 7.6 Hz, 1H, ArH), 7.34 (t, *J* = 7.4 Hz, 1H, ArH), 4.15 (s, 3H, CH_3_). ^13^C-NMR (100 MHz, DMSO-*d*_6_) *δ* 162.68, 142.51, 142.47, 141.50, 140.63, 139.79, 137.86, 136.16, 133.45, 132.20, 129.61, 129.18, 128.68, 128.32, 122.82, 122.60, 121.47, 120.92, 120.60, 118.70, 115.47, 112.77, 111.11, 30.24. HRMS calculated for C_25_H_19_N_6_O [M + H]^+^: 419.1615; found 419.1615.

*N′-((9-n-butyl-9H-pyrido[3,4-b]indol-3-yl)methylene)-9H-pyrido[3,4-b]indole-3-carbohydrazide* (**8i**): The compound was obtained as an ivory solid with 79% yield. ^1^H-NMR (400 MHz, DMSO-*d*_6_) *δ* 12.12 (s, 1H, NH), 9.46 (s, 1H, ArH), 9.17 (s, 1H, ArH), 9.02 (s, 1H, ArH), 8.64 (s, 1H, ArH), 8.47 (d, *J* = 7.6 Hz, 1H, CH), 8.36 (d, *J* = 8.0 Hz, 1H, ArH), 7.84 (d, *J* = 8.4 Hz, 1H, ArH), 7.80 (s, 1H, ArH), 7.75–7.68 (m, 2H, ArH), 7.63 (t, *J* = 7.6 Hz, 1H, ArH), 7.40 (t, *J* = 7.6 Hz, 1H, ArH), 7.34 (t, *J* = 7.6 Hz, 1H, ArH), 4.67 (t, *J* = 7.2 Hz, 2H, –CH_2_CH_2_CH_2_CH_3_), 1.92–1.84 (m, 2H, –CH_2_CH_2_CH_2_CH_3_), 1.42–1.31 (m, 2H, –CH_2_CH_2_CH_2_CH_3_), 0.92 (t, *J* = 7.6 Hz, 3H, –CH_2_CH_2_CH_2_CH_3_). ^13^C-NMR (100 MHz, DMSO-*d*_6_) *δ* 162.70, 142.48, 141.84, 141.51, 140.60, 139.77, 137.85, 135.67, 133.51, 132.16, 129.61, 129.19, 128.69, 128.42, 122.82, 122.69, 121.48, 121.00, 120.90, 120.60, 118.83, 115.47, 112.78, 111.30, 43.26, 31.50, 20.27, 14.19. HRMS calculated for C_28_H_25_N_6_O [M + H]^+^: 461.2084; found 461.2088.

*N′-((9-(3-phenylpropyl)-9H-pyrido[3,4-b]indol-3-yl)methylene)-9H-pyrido[3,4-b]indole-3-carbohydrazide* (**8j**): The compound was obtained as a yellow solid with 84% yield. ^1^H-NMR (400 MHz, DMSO-*d*_6_) *δ* 12.15 (s, 1H, NH), 9.35 (s, 1H, ArH), 9.15 (d, *J* = 1.2 Hz, 1H, ArH), 9.03 (s, 1H, ArH), 8.64 (s, 1H, ArH), 8.47 (d, *J* = 7.6 Hz, 1H, CH), 8.36 (d, *J* = 8.0 Hz, 1H, ArH), 7.81–7.78 (m, 2H, ArH), 7.73–7.68 (m, 2H, ArH), 7.66–7.63 (m, 1H, ArH), 7.42–7.38 (m, 1H, ArH), 7.37–7.34 (m, 1H, ArH), 7.32–7.27 (m, 3H, ArH), 7.25–7.20 (m, 3H, ArH), 4.69 (t, *J* = 7.2 Hz, 2H, ArCH_2_CH_2_CH_2_), 2.74 (t, *J* = 7.2 Hz, 2H, ArCH_2_CH_2_CH_2_), 2.27–2.18 (m, 2H, ArCH_2_CH_2_CH_2_). ^13^C-NMR (100 MHz, DMSO-*d*_6_) *δ* 162.70, 142.55, 141.79, 141.57, 141.51, 140.59, 139.77, 137.85, 135.59, 133.44, 131.99, 129.63, 129.20, 128.84, 128.82, 128.69, 128.62, 126.43, 122.83, 122.72, 121.48, 121.06, 120.96, 120.61, 118.85, 115.50, 112.78, 111.19, 43.12, 32.84, 30.87. HRMS calculated for C_33_H_27_N_6_O [M + H]^+^: 523.2241; found 523.2242.

*N′-((9-benzyl-9H-pyrido[3,4-b]indol-3-yl)methylene)-9H-pyrido[3,4-b]indole-3-carbohydrazide* (**8k**): The compound was obtained as a yellow solid with 86% yield. ^1^H-NMR (400 MHz, DMSO-*d*_6_) *δ* 12.32 (s, 1H, NH), 12.06 (s, 1H, NH), 9.13 (d, *J* = 0.8 Hz, 1H, ArH), 9.04 (s, 1H, ArH), 9.01 (d, *J* = 1.2 Hz, 1H, ArH), 8.92 (s, 1H, ArH), 8.83 (d, *J* = 0.8 Hz, 1H, ArH), 8.48 (t, *J* = 8.0 Hz, 2H, ArH, CH), 7.79 (d, *J* = 8.4 Hz, 1H, ArH), 7.71 (d, *J* = 8.4 Hz, 1H, ArH), 7.67–7.63 (m, 1H, ArH), 7.38–7.23 (m, 8H, ArH), 5.83 (s, 2H, ArCH_2_). ^13^C-NMR (100 MHz, DMSO-*d*_6_) *δ* 161.86, 150.06, 144.04, 141.98, 141.53, 139.51, 137.86, 137.69, 137.05, 132.81, 129.36, 129.26, 129.19, 128.79, 128.76, 128.00, 127.35, 127.31, 122.96, 122.90, 121.43, 121.30, 120.74, 120.64, 115.62, 112.80, 111.71, 111.15, 46.53. HRMS calculated for C_31_H_23_N_6_O [M + H]^+^: 495.1928; found 495.1922.

*N′-((9-(4-fluorobenzyl)-9H-pyrido[3,4-b]indol-3-yl)methylene)-9H-pyrido[3,4-b]indole-3-carbohydrazide* (**8l**): The compound was obtained as a light gray solid with 92% yield. ^1^H-NMR (400 MHz, DMSO-*d*_6_) *δ* 12.31 (s, 1H, NH), 12.04 (s, 1H, NH), 9.15 (d, *J* = 0.8 Hz, 1H, ArH), 9.03 (s, 1H, ArH), 9.00 (d, *J* = 1.2 Hz, 1H, ArH), 8.91 (s, 1H, ArH), 8.82 (d, *J* = 1.2 Hz, 1H, ArH), 8.48 (dd, *J* = 8.0, 4.8 Hz, 2H, CH, ArH), 7.81 (d, *J* = 8.4 Hz, 1H, ArH), 7.71 (d, *J* = 8.4 Hz, 1H, ArH), 7.68–7.61 (m, 2H, ArH), 7.38–7.30 (m, 4H, ArH), 7.17–7.11 (m, 2H, ArH), 5.82 (s, 2H, ArCH_2_). ^13^C-NMR (100 MHz, DMSO-*d*_6_) *δ* 161.96 (d, *J* = 241.8 Hz), 161.84, 150.05, 144.12, 141.86, 141.53, 139.51, 137.85, 136.94, 133.92 (d, *J* = 3.1 Hz), 132.80, 129.53, 129.45, 129.40, 129.24, 128.81, 128.79, 123.00 (d, *J* = 9.6 Hz), 121.43, 121.34, 120.80, 120.64, 116.11 (d, *J* = 21.3 Hz), 115.62, 112.79, 111.70, 111.12, 45.79. HRMS calculated for C_31_H_22_FN_6_O [M + H]^+^: 513.1834; found 513.1832.

*N′-((9H-pyrido[3,4-b]indol-3-yl)methylene)-1-methyl-9H-pyrido[3,4-b]indole-3-carbohydrazide* (**8m**): The compound was obtained as a yellow solid with 85% yield. ^1^H-NMR (400 MHz, DMSO-*d*_6_) *δ* 12.05 (s, 1H, NH), 12.02 (s, 1H, NH), 11.83 (s, 1H, NH), 8.95 (d, *J* = 0.8 Hz, 1H, ArH), 8.90 (s, 1H, ArH), 8.85 (s, 1H, ArH), 8.78 (s, 1H, ArH), 8.42 (t, *J* = 8.4 Hz, 2H, CH, ArH), 7.69–7.57 (m, 4H, ArH), 7.35–7.28 (m, 2H, ArH), 2.94 (s, 3H, CH_3_). ^13^C-NMR (100 MHz, DMSO-*d*_6_) *δ* 161.84, 150.09, 143.33, 141.66, 141.61, 141.28, 138.85, 136.87, 136.60, 133.94, 129.05, 128.88, 128.76, 127.95, 122.77, 122.70, 121.90, 121.33, 120.55, 120.26, 113.68, 112.71, 112.63, 111.84, 20.87. HRMS calculated for C_25_H_19_N_6_O [M + H]^+^: 419.1615; found 419.1617.

*N′-((9H-pyrido[3,4-b]indol-3-yl)methylene)-1-isopropyl-9H-pyrido[3,4-b]indole-3-carbohydrazide* (**8n**): The compound was obtained as a yellow solid with 91% yield. ^1^H-NMR (400 MHz, DMSO-*d*_6_) *δ* 15.65 (s, 1H, CONH), 12.17 (s, 1H, NH), 12.06 (s, 1H, NH), 9.22 (s, 1H, ArH), 8.88 (s, 1H, ArH), 8.65 (s, 1H, ArH), 8.42 (d, *J* = 7.6 Hz, 1H, CH), 8.34 (d, *J* = 7.6 Hz, 1H, ArH), 7.80 (s, 1H, ArH), 7.74–7.60 (m, 4H, ArH), 7.40–7.30 (m, 2H, ArH), 3.92–3.82 (m, 1H, CH(CH_3_)_2_), 1.68 (d, *J* = 6.8 Hz, 6H, CH(CH_3_)_2_). ^13^C-NMR (100 MHz, DMSO-*d*_6_) *δ* 162.96, 149.74, 142.52, 141.59, 141.29, 140.56, 139.03, 135.63, 135.24, 132.99, 129.52, 128.88, 128.79, 128.53, 122.59, 121.98, 121.19, 120.75, 120.50, 119.37, 113.80, 112.96, 112.71, 31.12, 22.22. HRMS calculated for C_27_H_23_N_6_O [M + H]^+^: 447.1928; found 447.1926.

*N′-((9H-pyrido[3,4-b]indol-3-yl)methylene)-1-phenyl-9H-pyrido[3,4-b]indole-3-carbohydrazide* (**8o**): The compound was obtained as a yellow solid with 91% yield. ^1^H-NMR (400 MHz, DMSO-*d*_6_) *δ* 15.97 (s, 1H, CONH), 12.27 (s, 1H, NH), 12.03 (s, 1H, NH), 9.05 (d, *J* = 5.2 Hz, 2H, ArH), 8.63 (s, 1H, ArH), 8.50 (d, *J* = 8.0 Hz, 1H, CH), 8.39 (d, *J* = 6.8 Hz, 2H, ArH), 8.33 (d, *J* = 7.6 Hz, 1H, ArH), 7.91 (t, *J* = 7.6 Hz, 2H, ArH), 7.81 (s, 1H, ArH), 7.79–7.72 (m, 3H, ArH), 7.68–7.62 (m, 2H, ArH), 7.39–7.34 (m, 2H, ArH). ^13^C-NMR (100 MHz, DMSO-*d*_6_) *δ* 162.62, 142.40, 142.07, 141.63, 141.12, 140.82, 139.66, 138.23, 135.57, 134.93, 132.94, 130.58, 129.75, 129.51, 129.20, 128.74, 122.61, 122.56, 121.71, 121.18, 120.84, 120.72, 119.28, 114.68, 113.22, 112.93. HRMS calculated for C_30_H_21_N_6_O [M + H]^+^: 481.1771; found 481.1772.

*N′-((9H-pyrido[3,4-b]indol-3-yl)methylene)-1-(4-methoxyphenyl)-9H-pyrido[3,4-b]indole-3-carbohydrazide* (**8p**): The compound was obtained as a yellow solid with 87% yield. ^1^H-NMR (400 MHz, DMSO-*d*_6_) *δ* 15.89 (s, 1H, CONH), 12.27 (s, 1H, NH), 11.96 (s, 1H, NH), 9.07 (s, 1H, ArH), 9.00 (s, 1H, ArH), 8.64 (s, 1H, ArH), 8.47 (d, *J* = 7.6 Hz, 1H, CH), 8.37–8.32 (m, 2H, ArH), 7.81 (s, 1H, ArH), 7.75 (dd, *J* = 8.0, 4.4 Hz, 2H, ArH), 7.69–7.61 (m, 2H, ArH), 7.44 (d, *J* = 8.8 Hz, 2H, ArH), 7.39–7.32 (m, 2H, ArH), 4.04 (s, 3H, OCH_3_). ^13^C-NMR (100 MHz, DMSO-*d*_6_) *δ* 162.69, 160.72, 142.41, 142.01, 141.63, 141.10, 140.77, 139.53, 135.60, 134.70, 133.00, 130.57, 130.49, 130.34, 129.52, 129.08, 128.77, 122.58, 122.54, 121.75, 121.20, 120.78, 120.73, 119.33, 114.92, 114.17, 113.22, 112.94, 56.01. HRMS calculated for C_31_H_23_N_6_O_2_ [M + H]^+^: 511.1877; found 511.1874.

*N′-((9H-pyrido[3,4-b]indol-3-yl)methylene)-1-benzyl-9H-pyrido[3,4-b]indole-3-carbohydrazide* (**8q**): The compound was obtained as an ivory solid with 83% yield. ^1^H-NMR (400 MHz, DMSO-*d*_6_) *δ* 12.14 (s, 1H, CONH), 11.99 (s, 1H, NH), 11.86 (s, 1H, NH), 8.98 (s, 1H, ArH), 8.91 (d, *J* = 2.4 Hz, 2H, ArH), 8.81 (s, 1H, ArH), 8.45 (d, *J* = 8.0 Hz, 1H, CH), 8.40 (d, *J* = 7.6 Hz, 1H, ArH), 7.72–7.66 (m, 2H, ArH), 7.65–7.55 (m, 4H, ArH), 7.35–7.28 (m, 4H, ArH), 7.21 (t, *J* = 7.2 Hz, 1H, ArH), 4.65 (s, 2H, ArCH_2_). ^13^C-NMR (100 MHz, DMSO-*d*_6_) *δ* 161.65, 150.29, 143.62, 143.18, 141.67, 141.39, 139.26, 138.89, 136.90, 136.13, 133.99, 129.39, 129.08, 129.04, 128.96, 128.83, 128.78, 126.71, 122.77, 122.70, 121.89, 121.32, 120.66, 120.27, 113.96, 112.77, 112.64, 111.94. HRMS calculated for C_31_H_23_N_6_O [M + H]^+^: 495.1928; found 495.1929.

*N′-((9H-pyrido[3,4-b]indol-3-yl)methylene)-9-methyl-9H-pyrido[3,4-b]indole-3-carbohydrazide* (**8r**): The compound was obtained as a light yellow solid with 81% yield. ^1^H-NMR (400 MHz, DMSO-*d*_6_) *δ* 12.31 (s, 1H, CONH), 11.82 (s, 1H, NH), 9.14 (s, 1H, ArH), 9.03 (s, 1H, ArH), 8.94 (d, *J* = 0.8 Hz, 1H, ArH), 8.92 (s, 1H, ArH), 8.78 (s, 1H, ArH), 8.52 (d, *J* = 7.6 Hz, 1H, CH), 8.41 (d, *J* = 8.0 Hz, 1H, ArH), 7.79 (d, *J* = 8.4 Hz, 1H, ArH), 7.73–7.68 (m, 1H, ArH), 7.64 (d, *J* = 8.4 Hz, 1H, ArH), 7.62–7.57 (m, 1H, ArH), 7.41 – 7.36 (m, 1H, ArH), 7.32–7.28 (m, 1H, ArH), 4.10 (s, 3H, CH_3_). ^13^C-NMR (100 MHz, DMSO-*d*_6_) *δ* 161.77, 150.35, 143.37, 142.39, 141.65, 139.73, 138.38, 136.86, 133.88, 131.51, 129.35, 129.04, 128.75, 128.36, 122.96, 122.70, 121.34, 121.09, 120.81, 120.25, 115.37, 112.62, 111.79, 110.90, 30.18. HRMS calculated for C_25_H_19_N_6_O [M + H]^+^: 419.1615; found 419.1618.

*N′-((9H-pyrido[3,4-b]indol-3-yl)methylene)-9-n-butyl-9H-pyrido[3,4-b]indole-3-carbohydrazide* (**8s**): The compound was obtained as a light yellow solid with 92% yield. ^1^H-NMR (400 MHz, DMSO-*d*_6_) *δ* 12.28 (s, 1H, CONH), 11.82 (s, 1H, NH), 9.15 (d, *J* = 0.8 Hz, 1H, ArH), 9.04 (d, *J* = 0.8 Hz, 1H, ArH), 8.95 (d, *J* = 0.8 Hz, 1H, ArH), 8.92 (s, 1H, ArH), 8.79 (s, 1H, ArH), 8.52 (d, *J* = 7.6 Hz, 1H, CH), 8.41 (d, *J* = 8.0 Hz, 1H, ArH), 7.81 (d, *J* = 8.4 Hz, 1H, ArH), 7.71–7.69 (m, 1H, ArH), 7.65 (d, *J* = 8.0 Hz, 1H, ArH), 7.62–7.57 (m, 1H, ArH), 7.40–7.35 (m, 1H, ArH), 7.32–7.28 (m, 1H, ArH), 4.63 (t, *J* = 6.8 Hz, 2H, –CH_2_CH_2_CH_2_CH_3_), 1.88–1.80 (m, 2H, –CH_2_CH_2_CH_2_CH_3_), 1.36–1.26 (m, 2H, –CH_2_CH_2_CH_2_CH_3_), 0.89 (t, *J* = 7.2 Hz, 3H, –CH_2_CH_2_CH_2_CH_3_). ^13^C-NMR (100 MHz, DMSO-*d*_6_) *δ* 161.76, 150.35, 143.36, 141.75, 141.65, 139.72, 137.94, 136.87, 133.89, 131.55, 129.35, 129.04, 128.75, 128.44, 123.07, 122.69, 121.34, 121.19, 120.80, 120.25, 115.45, 112.62, 111.80, 111.11, 49.07, 31.41, 20.21, 14.17. HRMS calculated for C_28_H_25_N_6_O [M + H]^+^: 461.2084; found 461.2085.

*N′-((9H-pyrido[3,4-b]indol-3-yl)methylene)-9-(3-phenylpropyl)-9H-pyrido[3,4-b]indole-3-carbohydrazide* (**8t**): The compound was obtained as a yellow solid with 88% yield. ^1^H-NMR (400 MHz, DMSO-*d*_6_) *δ* 12.32 (s, 1H, CONH), 11.85 (s, 1H, NH), 9.10 (s, 1H, ArH), 9.06 (s, 1H, ArH), 8.97 (s, 1H, ArH), 8.94 (s, 1H, ArH), 8.80 (s, 1H, ArH), 8.52 (d, *J* = 7.6 Hz, 1H, CH), 8.39 (d, *J* = 8.0 Hz, 1H, ArH), 7.76 (d, *J* = 8.0 Hz, 1H, ArH), 7.71–7.64 (m, 2H, ArH), 7.62–7.58 (m, 1H, ArH), 7.38 (t, *J* = 7.6 Hz, 1H, ArH), 7.32–7.25 (m, 3H, ArH), 7.19–7.16 (m, 3H, ArH), 4.65 (t, *J* = 7.2 Hz, 2H, ArCH_2_CH_2_CH_2_), 2.69 (t, *J* = 7.6 Hz, 2H, ArCH_2_CH_2_CH_2_), 2.22–2.14 (m, 2H, ArCH_2_CH_2_CH_2_). ^13^C-NMR (100 MHz, DMSO-*d*_6_) *δ* 161.81, 150.40, 143.37, 141.70, 141.64, 141.50, 139.79, 137.86, 136.88, 133.94, 131.43, 129.37, 129.03, 128.81, 128.73, 128.58, 128.55, 126.39, 123.09, 122.67, 121.34, 121.25, 120.85, 120.24, 115.49, 112.63, 111.84, 110.99, 43.06, 32.85, 30.82. HRMS calculated for C_33_H_27_N_6_O [M + H]^+^: 523.2241; found 523.2242.

*N′-((9H-pyrido[3,4-b]indol-3-yl)methylene)-9-benzyl-9H-pyrido[3,4-b]indole-3-carbohydrazide* (**8u**): The compound was obtained as a light yellow solid with 80% yield. ^1^H-NMR (400 MHz, DMSO-*d*_6_) *δ* 12.28 (s, 1H, CONH), 11.82 (s, 1H, NH), 9.19 (s, 1H, ArH), 9.07 (s, 1H, ArH), 8.95 (s, 1H, ArH), 8.90 (s, 1H, ArH), 8.78 (s, 1H, ArH), 8.55 (d, *J* = 8.0 Hz, 1H, CH), 8.41 (d, *J* = 8.0 Hz, 1H, ArH), 7.84 (d, *J* = 8.0 Hz, 1H, ArH), 7.70–7.63 (m, 2H, ArH, NH), 7.62–7.57 (m, 1H, ArH), 7.39 (t, *J* = 7.5 Hz, 1H, ArH), 7.34–7.24 (m, 6H, ArH), 5.91 (s, 2H, ArCH_2_). ^13^C-NMR (100 MHz, DMSO-*d*_6_) *δ* 161.70, 150.39, 143.33, 141.89, 141.64, 140.14, 138.04, 137.60, 136.87, 133.89, 131.84, 129.54, 129.23, 129.04, 128.84, 128.75, 128.09, 127.41, 123.17, 122.70, 121.40, 121.34, 121.11, 120.25, 115.52, 112.63, 111.80, 111.35, 46.73. HRMS calculated for C_31_H_23_N_6_O [M + H]^+^: 495.1928; found 495.1927.

*N′-((9H-pyrido[3,4-b]indol-3-yl)methylene)-9-(4-fluorobenzyl)-9H-pyrido[3,4-b]indole-3-carbohydrazide* (**8v**): The compound was obtained as a light yellow solid with 90% yield. ^1^H-NMR (400 MHz, DMSO-*d*_6_) *δ* 12.27 (s, 1H, CONH), 11.82 (s, 1H, NH), 9.20 (d, *J* = 1.2 Hz, 1H, ArH), 9.06 (d, *J* = 1.2 Hz, 1H, ArH), 8.94 (d, *J* = 1.2 Hz, 1H, ArH), 8.90 (s, 1H, ArH), 8.78 (s, 1H, ArH), 8.55 (d, *J* = 7.6 Hz, 1H, CH), 8.41 (d, *J* = 8.0 Hz, 1H, ArH), 7.86 (d, *J* = 8.4 Hz, 1H, ArH), 7.71–7.57 (m, 3H, ArH), 7.39 (t, *J* = 7.6 Hz, 1H, ArH), 7.35–7.28 (m, 3H, ArH), 7.19–7.12 (m, 2H, ArH), 5.90 (s, 2H, ArCH_2_). ^13^C-NMR (100 MHz, DMSO-*d*_6_) *δ* 162.00 (d, *J* = 242.1 Hz), 161.68, 150.43, 143.34, 141.77, 141.64, 140.21, 137.94, 136.88, 133.90, 133.82 (d, *J* = 3 Hz), 131.81, 129.59, 129.51, 129.04, 128.89, 128.74, 123.20, 122.70, 121.39 (d, *J* = 10.2 Hz), 121.17, 120.25, 116.05 (d, *J* = 21.3 Hz), 115.52, 112.62, 111.80, 111.32, 49.07. HRMS calculated for C_31_H_22_FN_6_O [M + H]^+^: 513.1834; found 513.1835.

*9-n-butyl-N′-((9-n-butyl-9H-pyrido[3,4-b]indol-3-yl)methylene)-9H-pyrido[3,4-b]indole-3-carbohydrazide* (**8w**): The compound was obtained as a yellow solid with 91% yield. ^1^H-NMR (400 MHz, DMSO-*d*_6_) *δ* 12.28 (s, 1H, CONH), 9.15 (s, 1H, ArH), 9.10 (s, 1H, ArH), 9.03 (s, 1H, ArH), 8.91 (s, 1H, ArH), 8.80 (s, 1H, ArH), 8.52 (d, *J* = 8.0 Hz, 1H, CH), 8.45 (d, *J* = 8.0 Hz, 1H, ArH), 7.82 (d, *J* = 8.4 Hz, 1H, ArH), 7.76 (d, *J* = 8.4 Hz, 1H, ArH), 7.71–7.64 (m, 2H, ArH), 7.40–7.32 (m, 2H, ArH), 4.63 (t, *J* = 6.8 Hz, 2H, –CH_2_CH_2_CH_2_CH_3_), 4.55 (t, *J* = 6.8 Hz, 2H, –CH_2_CH_2_CH_2_CH_3_), 1.88–1.79 (m, 4H, –CH_2_CH_2_CH_2_CH_3_), 1.37–1.27 (m, 4H, –CH_2_CH_2_CH_2_CH_3_), 0.92–0.88 (m, 6H, –CH_2_CH_2_CH_2_CH_3_). ^13^C-NMR (100 MHz, DMSO-*d*_6_) *δ* 161.75, 150.25, 143.59, 141.85, 141.75, 139.70, 137.95, 136.96, 132.61, 131.57, 129.36, 129.17, 128.44, 128.40, 123.08, 122.88, 121.19, 121.09, 120.80, 120.40, 115.45, 111.64, 111.12, 110.90, 43.12, 42.99, 31.42, 31.34, 20.24, 20.22, 14.18. HRMS calculated for C_32_H_33_N_6_O [M + H]^+^: 517.2710; found 517.2706.

*9-n-butyl-N′-((9-n-butyl-9H-pyrido[3,4-b]indol-3-yl)methylene)-1-methyl-9H-pyrido[3,4-b]indole-3-carbohydrazide* (**8x**): The compound was obtained as a yellow solid with 88% yield. ^1^H-NMR (400 MHz, CDCl_3_) *δ* 11.32 (s, 1H, CONH), 9.04 (s, 1H, ArH), 8.92 (s, 1H, ArH), 8.84 (s, 1H, ArH), 8.59 (s, 1H, CH), 8.25 (d, *J* = 8.0 Hz, 1H, ArH), 8.22 (d, *J* = 8.0 Hz, 1H, ArH), 7.64–7.59 (m, 2H, ArH), 7.47 (dd, *J* = 8.4, 2.8 Hz, 2H, ArH), 7.35 (t, *J* = 7.6 Hz, 2H, ArH), 4.54 (t, *J* = 7.6 Hz, 2H, –CH_2_CH_2_CH_2_CH_3_), 4.36 (t, *J* = 7.2 Hz, 2H, –CH_2_CH_2_CH_2_CH_3_), 3.09 (s, 3H, CH_3_), 1.94–1.81 (m, 4H, –CH_2_CH_2_CH_2_CH_3_), 1.53–1.36 (m, 4H, –CH_2_CH_2_CH_2_CH_3_), 1.00 (t, *J* = 7.6 Hz, 3H, –CH_2_CH_2_CH_2_CH_3_), 0.97 (t, *J* = 7.2 Hz, 3H, –CH_2_CH_2_CH_2_CH_3_). ^13^C-NMR (100 MHz, CDCl_3_) *δ* 161.29, 148.92, 142.53, 141.84, 141.81, 139.67, 137.77, 137.00, 136.59, 130.81, 129.58, 129.19, 128.72, 128.53, 122.59, 121.98, 121.69, 121.48, 120.56, 120.27, 113.75, 113.00, 109.97, 109.60, 44.84, 43.34, 33.02, 31.27, 23.71, 20.52, 20.21, 13.87, 13.81. HRMS calculated for C_33_H_35_N_6_O [M + H]^+^: 531.2867; found 531.2873.

*9-benzyl-N′-((9-n-butyl-9H-pyrido[3,4-b]indol-3-yl)methylene)-1-methyl-9H-pyrido[3,4-b]indole-3-carbohydrazide* (**8y**): The compound was obtained as a yellow solid with 84% yield. ^1^H-NMR (400 MHz, CDCl_3_) *δ* 11.27 (s, 1H, CONH), 9.03 (s, 1H, ArH), 8.99 (s, 1H, ArH), 8.84 (s, 1H, ArH), 8.55 (s, 1H, CH), 8.26 (dd, *J* = 10.4, 8.0 Hz, 2H, ArH), 7.64–7.55 (m, 2H, ArH), 7.47 (d, *J* = 8.4 Hz, 1H, ArH), 7.41–7.34 (m, 3H, ArH), 7.33–7.23 (m, 3H, ArH), 7.01–6.95 (m, 2H, ArH), 5.83 (s, 2H, ArCH_2_), 4.37 (t, *J* = 7.2 Hz, 2H, –CH_2_CH_2_CH_2_CH_3_), 2.93 (s, 3H, CH_3_), 1.94–1.87 (m, 2H, –CH_2_CH_2_CH_2_CH_3_), 1.47–1.37 (m, 2H, –CH_2_CH_2_CH_2_CH_3_), 0.96 (t, *J* = 7.2 Hz, 3H, –CH_2_CH_2_CH_2_CH_3_). ^13^C-NMR (100 MHz, CDCl_3_) *δ* 161.19, 149.29, 142.63, 142.26, 141.78, 140.15, 138.33, 137.49, 137.06, 131.05, 129.80, 129.13, 129.07, 128.91, 128.64, 127.72, 125.32, 122.56, 122.05, 121.75, 121.51, 121.03, 120.23, 113.81, 113.00, 110.11, 109.59, 48.30, 43.32, 31.27, 23.32, 20.52, 13.82. HRMS calculated for C_36_H_33_N_6_O [M + H]^+^: 565.2710; found 565.2715.

*N′-((9-benzyl-1-methyl-9H-pyrido[3,4-b]indol-3-yl)methylene)-9-n-butyl-9H-pyrido[3,4-b]indole-3-carbohydrazide* (**8z**): The compound was obtained as a yellow solid with 85% yield. ^1^H-NMR (400 MHz, CDCl_3_) *δ* 11.22 (s, 1H, CONH), 9.06 (s, 1H, ArH), 8.92 (s, 1H, ArH), 8.78 (s, 1H, ArH), 8.50 (s, 1H, CH), 8.25 (t, *J* = 7.2 Hz, 2H, ArH), 7.65–7.61 (m, 1H, ArH), 7.56–7.48 (m, 2H, ArH), 7.39–7.32 (m, 3H, ArH), 7.31 – 7.23 (m, 3H, ArH), 7.00 (d, *J* = 6.4 Hz, 2H, ArH), 5.76 (s, 2H, ArCH_2_), 4.39 (t, *J* = 7.2 Hz, 2H, –CH_2_CH_2_CH_2_CH_3_), 2.88 (s, 3H, CH_3_), 1.94–1.87 (m, 2H, –CH_2_CH_2_CH_2_CH_3_), 1.46–1.36 (m, 2H, –CH_2_CH_2_CH_2_CH_3_), 0.97 (t, *J* = 7.2 Hz, 3H, –CH_2_CH_2_CH_2_CH_3_). ^13^C-NMR (100 MHz, CDCl_3_) *δ* 161.36, 149.11, 142.47, 142.18, 141.57, 140.82, 138.81, 137.87, 137.70, 136.08, 130.00, 129.88, 129.05, 128.95, 128.81, 128.75, 127.59, 125.40, 122.41, 122.16, 121.76, 121.48, 120.69, 120.57, 115.31, 111.46, 109.91, 109.81, 48.26, 43.48, 31.32, 23.00, 20.52, 13.81. HRMS calculated for C_36_H_33_N_6_O [M + H]^+^: 565.2710; found 565.2703.

*N′-((9-benzyl-1-methyl-9H-pyrido[3,4-b]indol-3-yl)methylene)-9-n-butyl-1-methyl-9H-pyrido[3,4-b]indole-3-carbohydrazide* (**8aa**): The compound was obtained as a yellow solid with 83% yield. ^1^H-NMR (400 MHz, CDCl_3_) *δ* 11.30 (s, 1H, CONH), 8.90 (d, *J* = 4.4 Hz, 2H, ArH), 8.51 (s, 1H, CH, ArH), 8.26 (d, *J* = 7.6 Hz, 1H, ArH), 8.22 (d, *J* = 7.6 Hz, 1H, ArH), 7.63–7.59 (m, 1H, ArH), 7.56–7.52 (m, 1H, ArH), 7.46 (d, *J* = 8.4 Hz, 1H, ArH), 7.38–7.29 (m, 4H, ArH), 7.29–7.22 (m, 2H, ArH), 7.01–6.98 (m, 2H, ArH), 5.74 (s, 2H, ArCH_2_), 4.51 (t, *J* = 7.6 Hz, 2H, –CH_2_CH_2_CH_2_CH_3_), 3.06 (s, 3H, CH_3_), 2.88 (s, 3H, CH_3_), 1.88–1.80 (m, 2H, –CH_2_CH_2_CH_2_CH_3_), 1.51–1.42 (m, 2H, –CH_2_CH_2_CH_2_CH_3_), 1.00 (t, *J* = 7.6 Hz, 3H, –CH_2_CH_2_CH_2_CH_3_). ^13^C-NMR (100 MHz, CDCl_3_) *δ* 161.26, 142.48, 142.10, 141.79, 140.81, 139.64, 137.73, 137.68, 136.55, 136.02, 129.90, 129.54, 129.06, 128.78, 128.52, 127.60, 125.40, 125.32, 122.17, 121.97, 121.73, 121.68, 120.71, 120.55, 113.67, 111.45, 109.97, 109.91, 48.24, 44.82, 33.01, 23.71, 22.93, 20.21, 13.86. HRMS calculated for C_37_H_35_N_6_O [M + H]^+^: 579.2867; found 579.2861.

*9-benzyl-N′-((9-benzyl-1-methyl-9H-pyrido[3,4-b]indol-3-yl)methylene)-1-methyl-9H-pyrido[3,4-b]indole-3-carbohydrazide* (**8ab**): The compound was obtained as a yellow solid with 85% yield. ^1^H-NMR (400 MHz, CDCl_3_) *δ* 11.25 (s, 1H, CONH), 8.96 (s, 1H, ArH), 8.90 (s, 1H, ArH), 8.48 (s, 1H, ArH), 8.28–8.25 (m, 2H, ArH, CH), 7.58–7.52 (m, 2H, ArH), 7.39–7.32 (m, 4H, ArH), 7.30–7.26 (m, 3H, ArH), 7.25–7.21 (m, 2H, ArH), 7.00–6.95 (m, 5H, ArH), 5.78 (s, 2H, ArCH_2_), 5.74 (s, 2H, ArCH_2_), 2.88 (s, 3H, CH_3_), 2.86 (s, 3H, CH_3_). ^13^C-NMR (100 MHz, CDCl_3_) *δ* 161.08, 142.44, 142.22, 142.14, 140.82, 140.09, 138.28, 137.70, 137.49, 136.98, 136.05, 129.83, 129.73, 129.11, 129.04, 128.88, 128.72, 127.70, 127.58, 125.39, 125.30, 122.14, 122.01, 121.75, 121.72, 120.99, 120.68, 113.67, 111.34, 110.09, 109.90, 48.24, 23.31. HRMS calculated for C_40_H_33_N_6_O [M + H]^+^: 613.2710; found 613.2719.

### 3.5. MTT Assay

Target compounds were assayed by the MTT method for determining cytotoxic activity as described previously [41]. The panel of cell lines included the human umbilical vein cell line EA.HY926, Lewis lung carcinoma (LLC), gastric carcinoma (BGC-823), murine colon carcinoma (CT-26), liver carcinoma (Bel-7402), and breast carcinoma (MCF-7). Cell lines were obtained from Shanghai Cell Institute, Chinese Academy of Science. Growth inhibition rates were calculated with the following equitation: Inhibition ratio (%) = $\frac{{\mathrm{OD}}_{compd}-{\mathrm{OD}}_{blank}}{{\mathrm{OD}}_{DMSO}-{\mathrm{OD}}_{blank}}\times$ 100%. The half-maximal inhibitory concentration (IC_50_) of each compound was calculated using GraphPad Prism software (version 6.0).

### 3.6. CAM Assay in Vivo

To determine in vivo anti-angiogenic activity of heterobivalent β-carbolines, a CAM assay was performed as previously described [39]. In brief, five-day-old fertilized chicken eggs were purchased from a local hatchery. All the eggs were incubated at 37 °C in an incubator. We injected 0.5 mL of saline, and the eggs were incubated horizontally to allow the CAM to detach from the shell to make a bogus chamber. Compound **8z** was prepared in gelatin sponge discs at concentrations of 0.5, 5.0, and 50 µM/disc. CA4P was used as a positive control drug. Discs containing the vehicle only (DMSO) were used as negative controls. A small window opening was made in the shell, and the discs were directly applied onto the CAM. The square opening was covered with sterilized surgical tape, and the embryos were incubated for 48 h at 38.5 °C. The CAMs were photographed under a dissecting microscope, and blood vessels in each CAM were counted. The results are presented as a mean percentage of inhibition compared to the control ± SD, *n* = 3.

## 4. Conclusions

On the basis of our previous work, this study has focused on the synthesis of a series of heterobivalent β-carbolines bearing an acylhydrazone bond (**8a**–**ab**). All of the target compounds were investigated for their in vitro antiproliferative activity using the MTT-based assay against five cancer cell lines (LLC, BGC-823, CT-26, Bel-7402, and MCF-7). Most compounds showed medium antiproliferative activities against the tested cancer cell lines. In particular, compound **8z** showed antitumor activities with IC_50_ values of 9.9, 8.6, 6.2, 9.9, and 5.7 µM against the LLC, BGC-823, CT-26, Bel-7402, and MCF-7 cell lines, respectively. The anti-angiogenic activity of compound **8z** was comparable with CA4P in an in vivo CAM assay at the 5 μM level.

## Figures and Tables

**Table 1:**
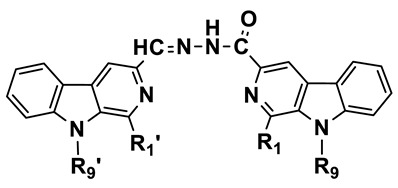
Cytotoxic activity of acylhydrazone linked heterobivalent β-carbolines **8a**–**ab** in vitro^a^ (IC_50_, µM^b^).

Comp.	R_1_′	R_9_′	R_1_	R_9_	IC_50_(μM) ± SD				
					LLC^c^	BGC823	CT-26	Bel-7402	MCF-7
**8a**	H	H	H	H	91.9 ± 6.8	68.4 ± 6.2	63.6 ± 7.5	86.3 ± 9.4	56.6 ± 3.5
**8b**	CH_3_	H	H	H	81.2 ± 7.5	3.2 ± 0.7	94.7 ± 11.8	45.7 ± 3.2	40.6 ± 5.4
**8c**	CH(CH_3_)_2_	H	H	H	76.7 ± 6.3	9.7 ± 0.9	38.5 ± 10.4	14.6 ± 3.2	15.3 ± 2.7
**8d**	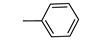	H	H	H	61.3 ± 5.5	6.3 ± 1.6	57.2 ± 4.1	20.7 ± 3.3	11.5 ± 2.1
**8e**	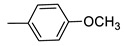	H	H	H	58.5 ± 5.7	10.8 ± 1.4	68.4 ± 4.8	54.3 ± 6.9	34.2 ± 5.2
**8f**	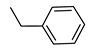	H	H	H	70.3 ± 6.8	10.2 ± 2.3	22.2 ± 3.2	11.7 ± 0.9	17.6 ± 2.6
**8g**	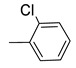	H	H	H	48.8 ± 3.8	61.4 ± 7.6	57.4 ± 4.5	52.6 ± 5.8	30.9 ± 4.2
**8h**	H	CH_3_	H	H	71.3 ± 10.8	78.6 ± 6.5	43.4 ± 4.2	41.2 ± 3.1	21.4 ± 5.3
**8i**	H	n-butyl	H	H	30.7 ± 2.9	27.4 ± 3.2	57.1 ± 5.6	15.3 ± 3.5	14.0 ± 3.5
**8j**	H	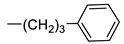	H	H	40.7 ± 4.7	25.6 ± 2.1	23.4 ± 2.8	68.9 ± 5.4	21.6 ± 3.7
**8k**	H	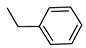	H	H	17.4 ± 4.6	52.9 ± 5.8	15.6 ± 4.1	16.3 ± 4.7	18.5 ± 5.1
**8l**	H	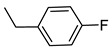	H	H	29.2 ± 5.1	69.9 ± 8.4	15.8 ± 3.2	15.5 ± 2.9	17.3 ± 3.4
**8m**	H	H	CH_3_	H	24.7 ± 3.9	17.5 ± 2.1	13.8 ± 2.7	15.6 ± 2.9	16.0 ± 4.3
**8n**	H	H	CH(CH_3_)_2_	H	38.2 ± 5.7	19.9 ± 2.9	22.0 ± 3.7	41.1 ± 7.8	14.5 ± 2.1
**8o**	H	H	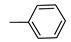	H	72.1 ± 6.5	9.1 ± 1.4	56.3 ± 4.2	73.7 ± 9.8	14.4 ± 4.2
**8p**	H	H	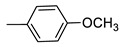	H	68.4 ± 9.4	76.6 ± 5.2	69.5 ± 11.7	70.2 ± 8.5	>100
**8q**	H	H		H	20.3 ± 1.3	24.5 ± 5.1	13.4 ± 3.7	13.3 ± 2.1	14.7 ± 4.2
**8r**	H	H	H	CH_3_	88.7 ± 5.6	34.8 ± 4.6	14.0 ± 3.3	12.4 ± 4.2	68.9 ± 7.5
**8s**	H	H	H	n-butyl	86.5 ± 10.7	2.4 ± 0.6	3.1 ± 1.2	21.5 ± 4.2	16.8 ± 1.9
**8t**	H	H	H	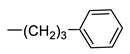	13.3 ± 2.3	14.7 ± 2.1	12.6 ± 1.7	20.1 ± 5.2	2.5 ± 0.4
**8u**	H	H	H	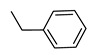	83.2 ± 14.6	5.3 ± 0.9	2.7 ± 0.3	8.7 ± 1.5	2.8 ± 0.7
**8v**	H	H	H	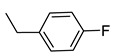	41.5 ± 5.1	43.4 ± 3.6	33.5 ± 2.7	46.7 ± 8.3	76.8 ± 5.2
**8w**	H	n-butyl	H	n-butyl	58.6 ± 4.4	28.2 ± 2.4	33.3 ± 6.2	25.3 ± 7.5	73.0 ± 12.9
**8x**	H	n-butyl	CH_3_	n-butyl	70.2 ± 6.4	66.2 ± 5.8	55.8 ± 4.7	72.2 ± 11.4	49.5 ± 9.8
**8y**	H	n-butyl	CH_3_	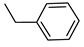	57.1 ± 4.6	39.2 ± 3.4	41.8 ± 5.3	60.4 ± 4.2	>100
**8z**	CH_3_	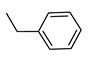	H	n-butyl	9.9 ± 0.9	8.6 ± 1.4	6.2 ± 2.5	9.9 ± 0.5	5.7 ± 1.2
**8aa**	CH_3_	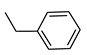	CH_3_	n-butyl	44.5 ± 4.9	54.8 ± 3.2	44.3 ± 5.5	58.6 ± 4.3	28.2 ± 3.2
**8ab**	CH_3_	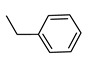	CH_3_	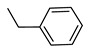	18.4 ± 2.7	61.6 ± 4.3	19.9 ± 3.5	44.7 ± 5.7	30.1 ± 4.2
**DDP**					21.3 ± 1.1	8.4 ± 0.7	4.2 ± 0.7	15.4 ± 1.9	10.5 ± 2.3

^a^ Data represent the mean values of three independent determinations; ^b^ cytotoxicity as IC_50_ for each cell line is the concentration of compound which reduced the optical density of treated cells by 50% with respect to untreated cells using the MTT assay. The data represent the mean values ± SD of at least three independent determinations. Values > 100 μM indicate less than 50% growth inhibition at > 100 μM; ^c^ cell lines include Lewis lung carcinoma (LLC), gastric carcinoma (BGC), murine colon carcinoma (CT-26), liver carcinoma (Bel-7402), and breast carcinoma (MCF-7).

## Supplementary Materials

The following are available online, ^1^H and ^13^C-NMR spectra for the target compounds are available online.
