# Supplementary material for: Design, Synthesis, and Biological Evaluation of Novel N-Acylhydrazone Bond Linked Heterobivalent β-Carbolines as Potential Anticancer Agents
Source: Molecules. 2019 Aug 14;24(16):2950. doi: 10.3390/molecules24162950 (PMC6720801; doi:10.3390/molecules24162950)
Supplement: Supplementary file 1 [file molecules-24-02950-s001.pdf]

Supplementary Information

Design, synthesis, and biological evaluation of novel N-acylhydrazone bond linked heterobivalent β-carbolines as potential anticancer agents

Xiao-fei Chen [1], Liang Guo [1], Qin Ma [2], Wei Chen [2], Wen-xi Fan [2], Jie Zhang [1,*]

[1] *School of Chemistry and Chemical Engineering, Key Laboratory for Green Processing of Chemical Engineering of Xinjiang Bingtuan, Shihezi University, Shihezi 832003, P R China;*

[2] *Xinjiang Huashidan Pharmaceutical Research Co. Ltd., 175 He Nan East Road, Urumqi 830011, P R China;*

Contents

1. **Figure S1** the ^{1}H NMR and ^{13}C NMR spectrum of compound **8a**··········S3
2. **Figure S2** the ^{1}H NMR and ^{13}C NMR spectrum of compound **8b**··········S4
3. **Figure S3** the ^{1}H NMR and ^{13}C NMR spectrum of compound **8c**··········S5
4. **Figure S4** the ^{1}H NMR and ^{13}C NMR spectrum of compound **8d**··········S6
5. **Figure S5** the ^{1}H NMR and ^{13}C NMR spectrum of compound **8e**··········S7
6. **Figure S6** the ^{1}H NMR and ^{13}C NMR spectrum of compound **8f**··········S8
7. **Figure S7** the ^{1}H NMR and ^{13}C NMR spectrum of compound **8g**··········S9
8. **Figure S8** the ^{1}H NMR and ^{13}C NMR spectrum of compound **8h**·········S10
9. **Figure S9** the ^{1}H NMR and ^{13}C NMR spectrum of compound **8i**··········S11
10. **Figure S10** the ^{1}H NMR and ^{13}C NMR spectrum of compound **8j**········S12
11. **Figure S11** the ^{1}H NMR and ^{13}C NMR spectrum of compound **8k**·······S13
12. **Figure S12** the ^{1}H NMR and ^{13}C NMR spectrum of compound **8l**········S14
13. **Figure S13** the ^{1}H NMR and ^{13}C NMR spectrum of compound **8m**······S15
14. **Figure S14** the ^{1}H NMR and ^{13}C NMR spectrum of compound **8n**·······S16
15. **Figure S15** the ^{1}H NMR and ^{13}C NMR spectrum of compound **8o**··········S17
16. **Figure S16** the ^{1}H NMR and ^{13}C NMR spectrum of compound **8p**··········S18
17. **Figure S17** the ^{1}H NMR and ^{13}C NMR spectrum of compound **8q**··········S19
18. **Figure S18** the ^{1}H NMR and ^{13}C NMR spectrum of compound **8r**··········S20
19. **Figure S19** the ^{1}H NMR and ^{13}C NMR spectrum of compound **8s**··········S21

20. **Figure S20** the ^{1}H NMR and ^{13}C NMR spectrum of compound **8t**···········S22

21. **Figure S21** the ^{1}H NMR and ^{13}C NMR spectrum of compound **8u**···········S23

22. **Figure S22** the ^{1}H NMR and ^{13}C NMR spectrum of compound **8v**···········S24

23. **Figure S23** the ^{1}H NMR and ^{13}C NMR spectrum of compound **8w**···········S25

24. **Figure S24** the ^{1}H NMR and ^{13}C NMR spectrum of compound **8x**···········S26

25. **Figure S25** the ^{1}H NMR and ^{13}C NMR spectrum of compound **8y**···········S27

26. **Figure S26** the ^{1}H NMR and ^{13}C NMR spectrum of compound **8z**···········S28

27. **Figure S27** the ^{1}H NMR and ^{13}C NMR spectrum of compound **8aa**···········S29

28. **Figure S28** the ^{1}H NMR and ^{13}C NMR spectrum of compound **8ab**···········S30

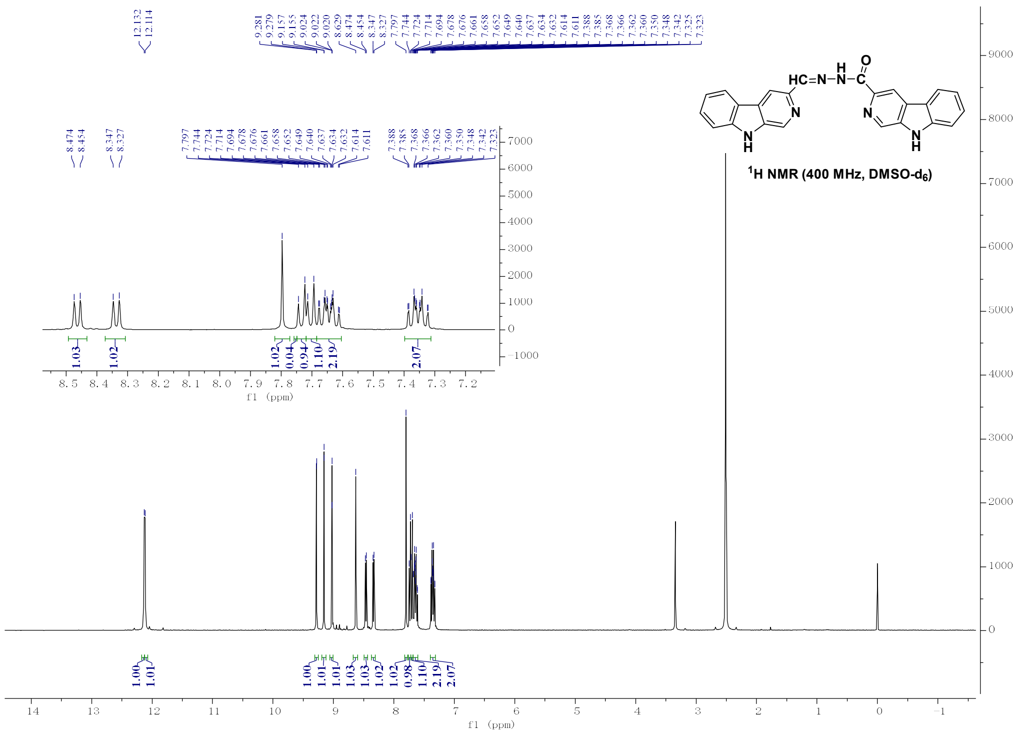

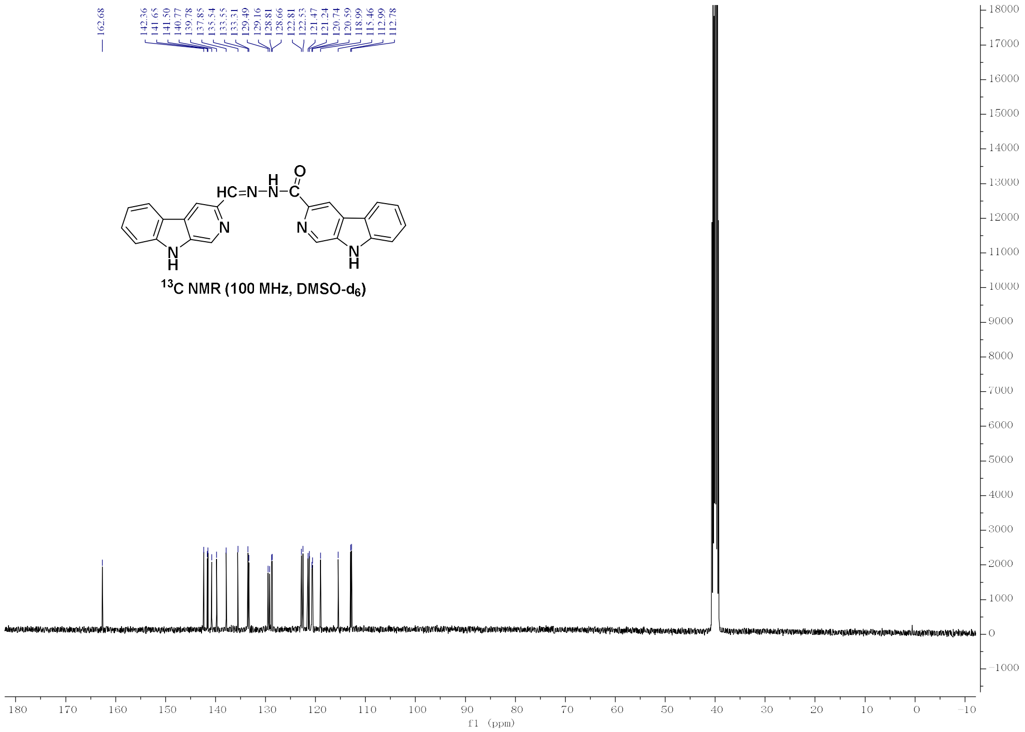

Figure S1 the ^{1}H NMR spectrum and ^{13}C NMR spectrum of compound **8a**

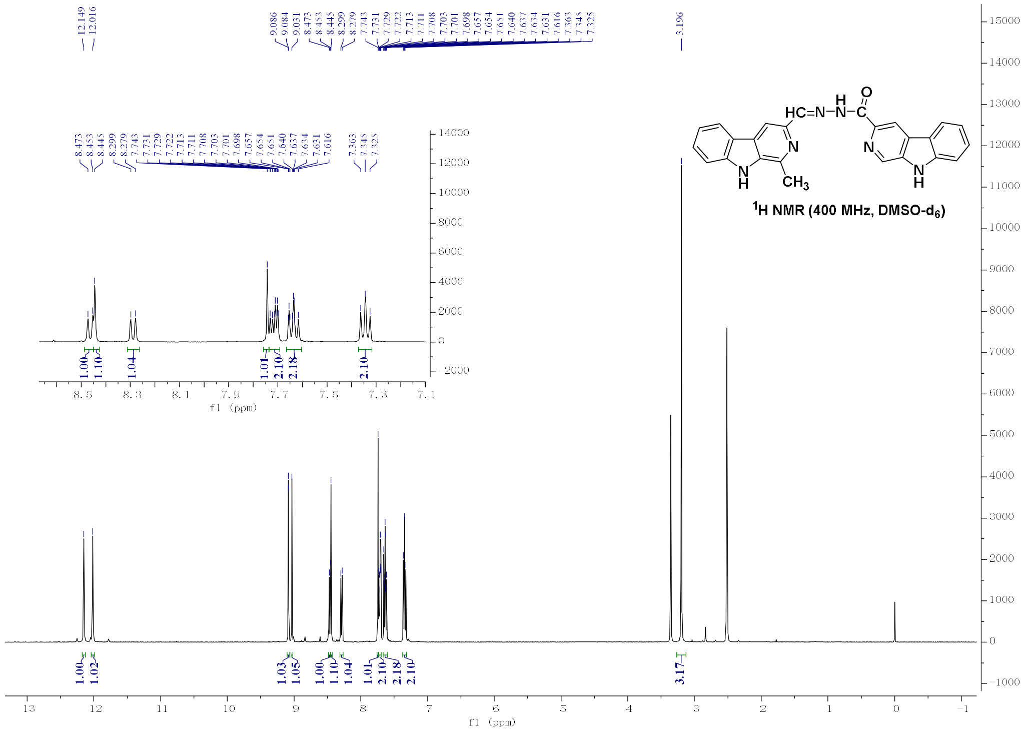

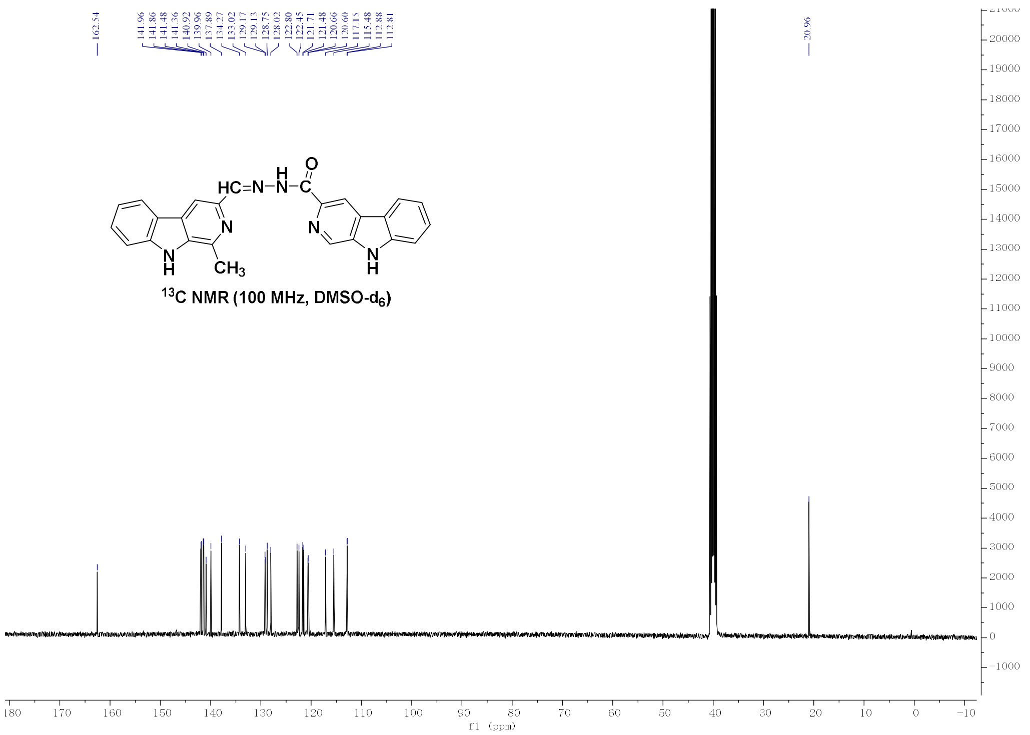

Figure S2 the ^{1}H NMR spectrum and ^{13}C NMR spectrum of compound **8b**

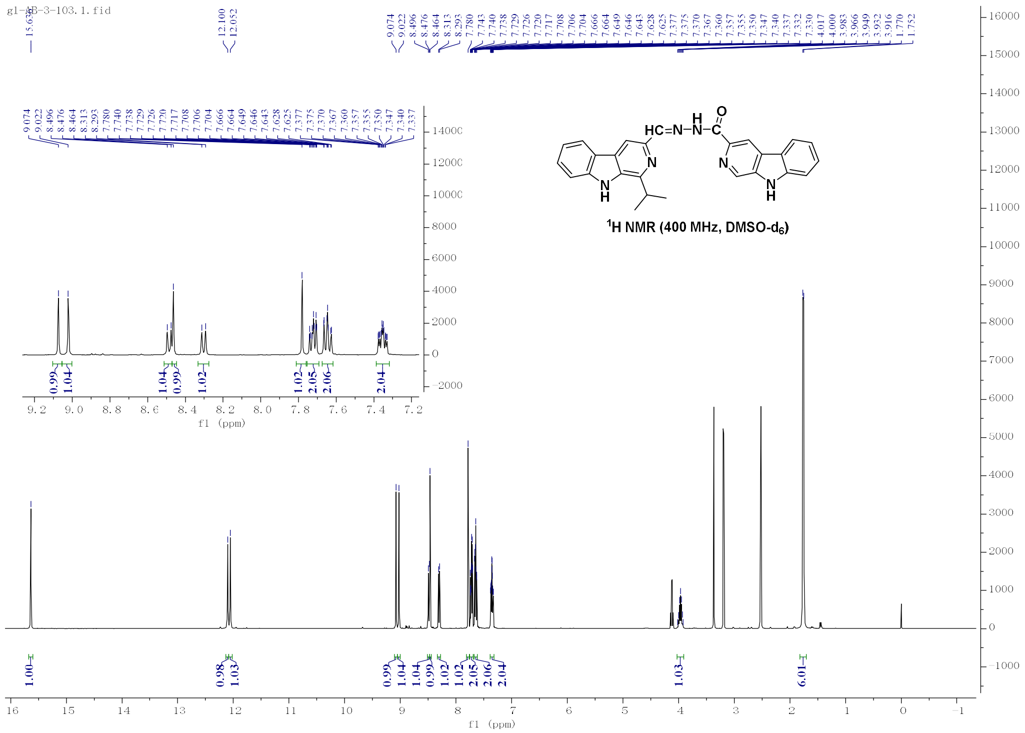

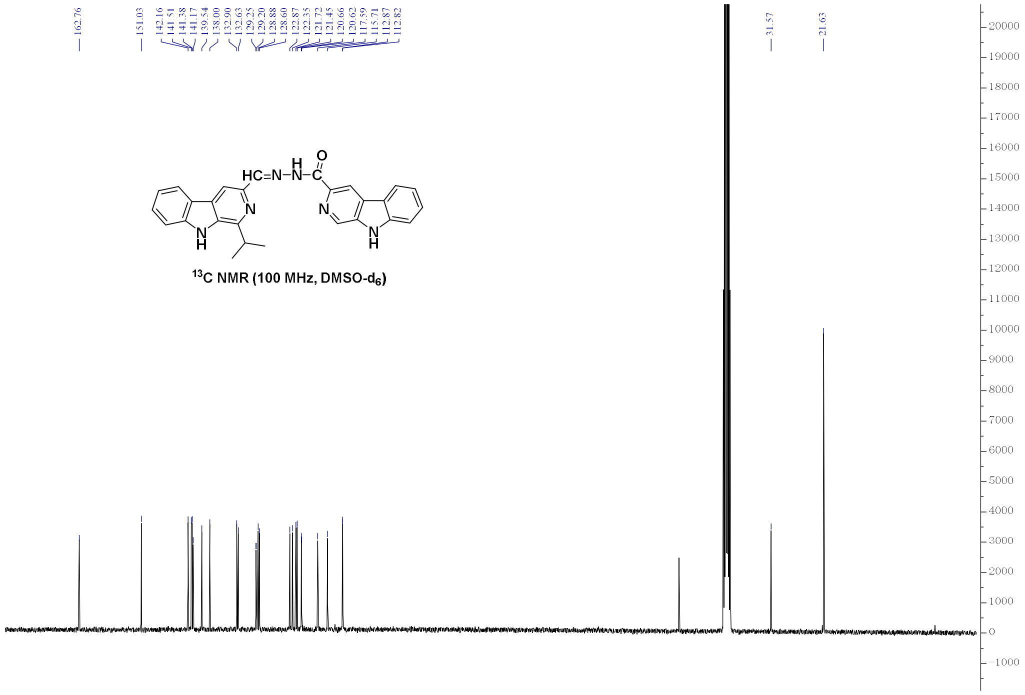

Figure S3 the ^{1}H NMR spectrum and ^{13}C NMR spectrum of compound **8c**

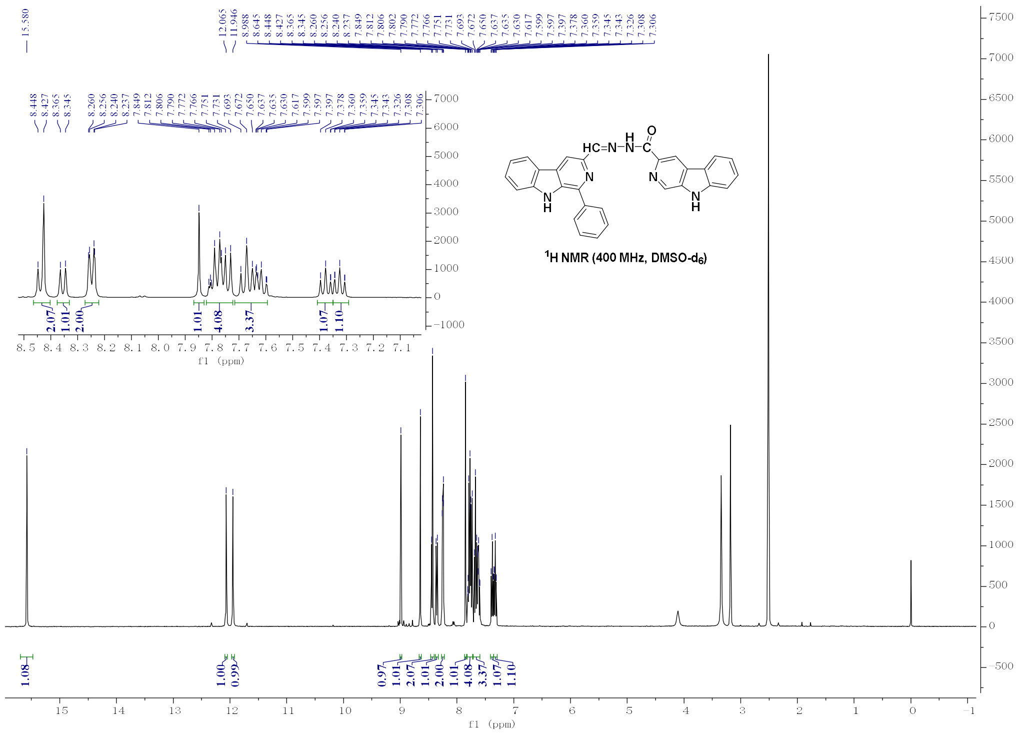

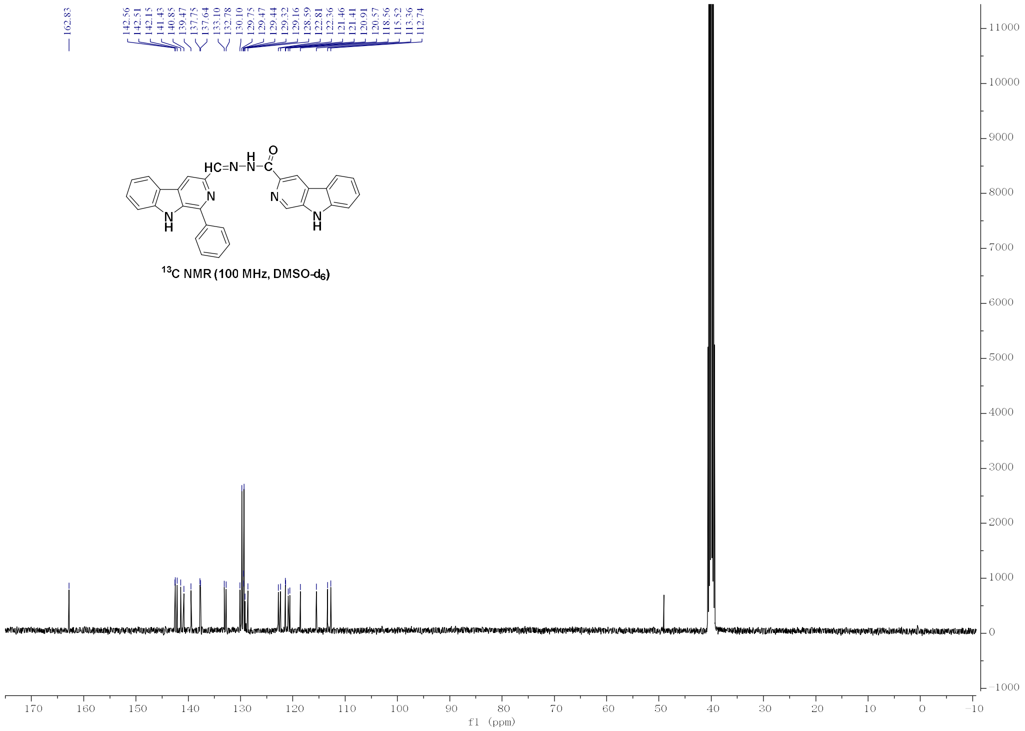

Figure S4 the ^{1}H NMR spectrum and ^{13}C NMR spectrum of compound **8d**

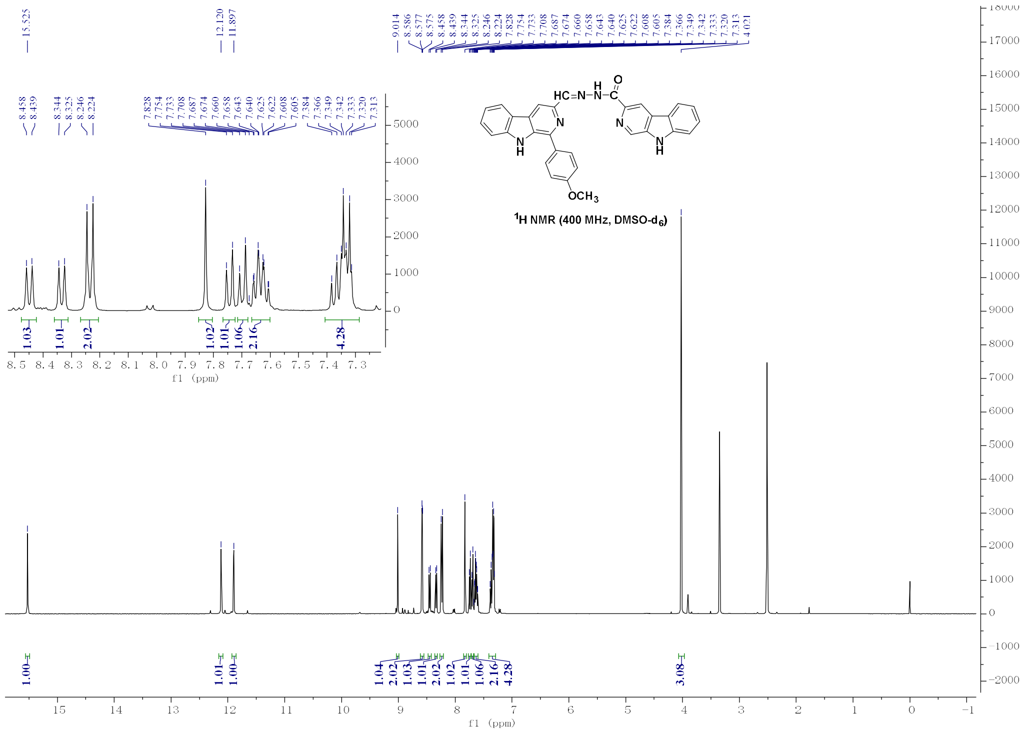

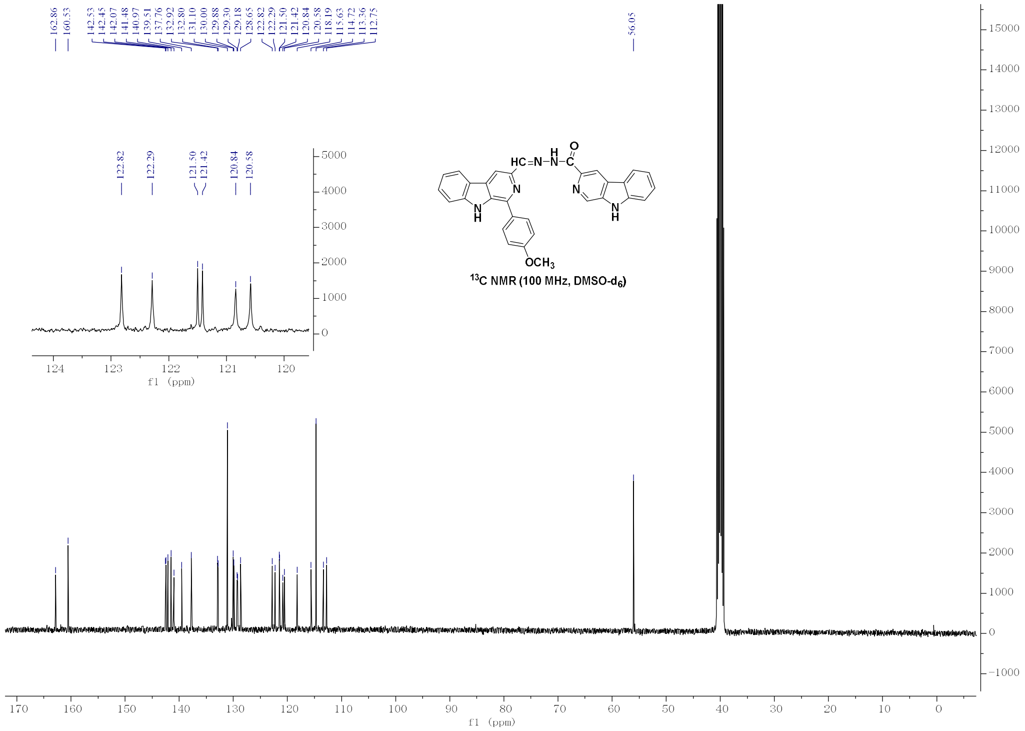

Figure S5 the ^{1}H NMR spectrum and ^{13}C NMR spectrum of compound **8e**

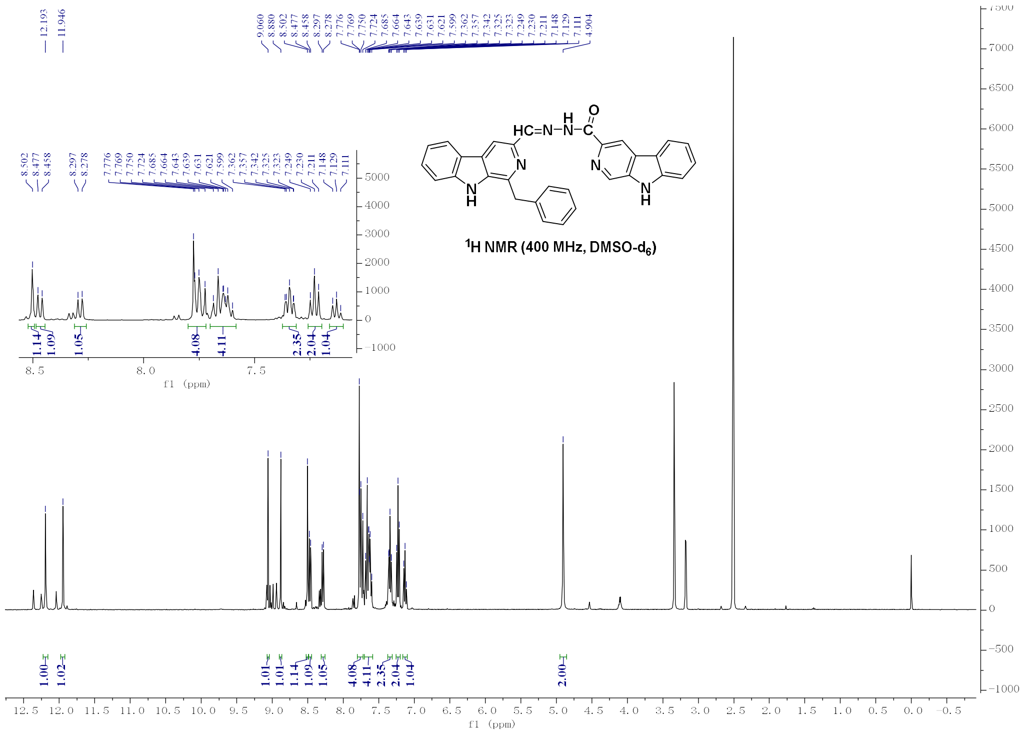

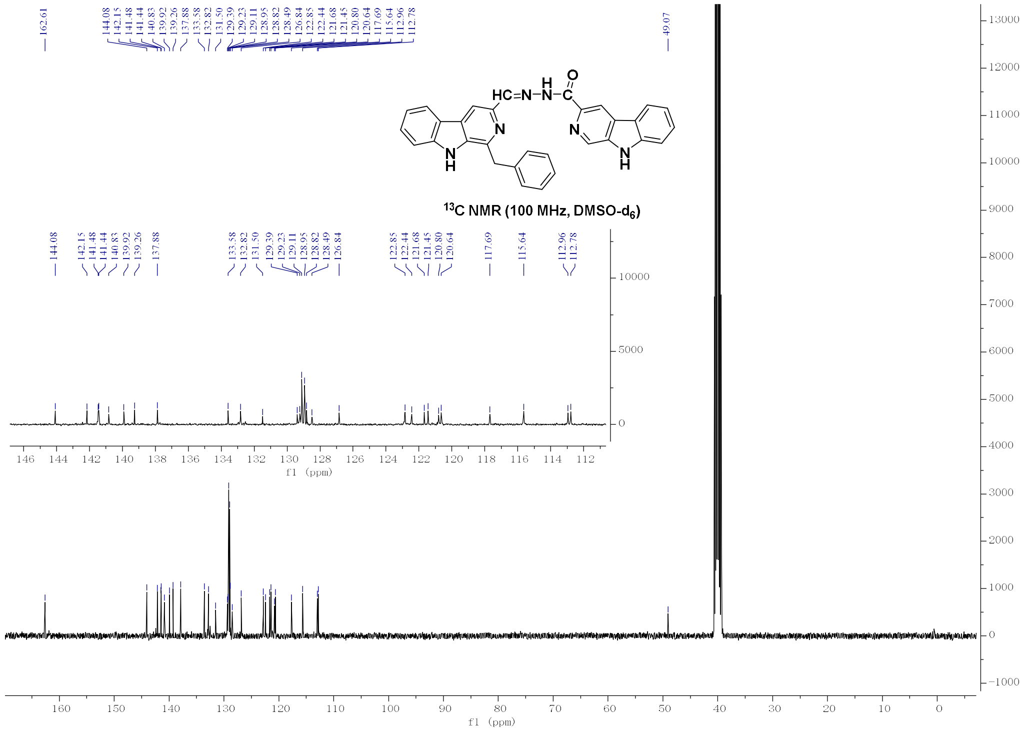

Figure S6 the ^{1}H NMR spectrum and ^{13}C NMR spectrum of compound **8f**

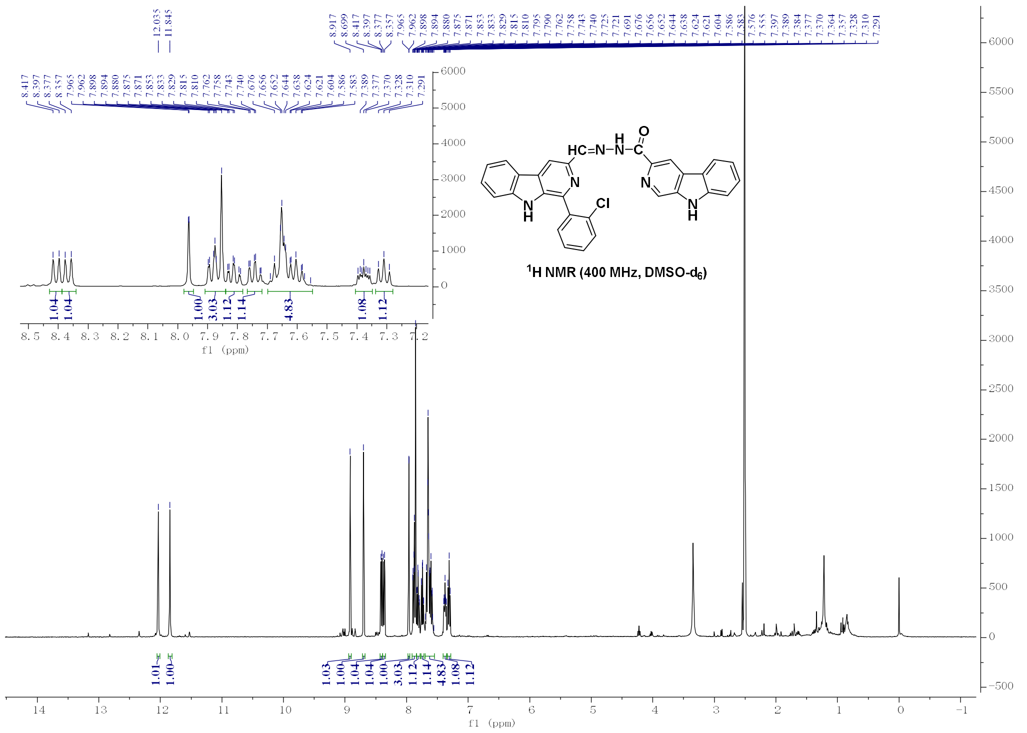

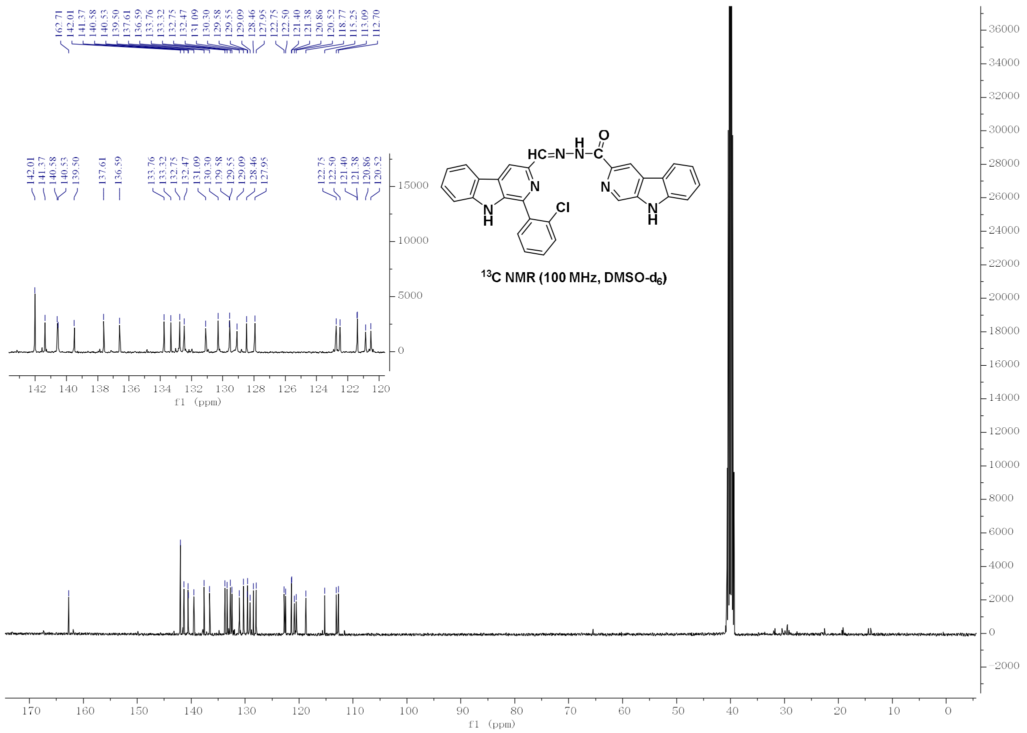

Figure S7 the ^{1}H NMR spectrum and ^{13}C NMR spectrum of compound **8g**

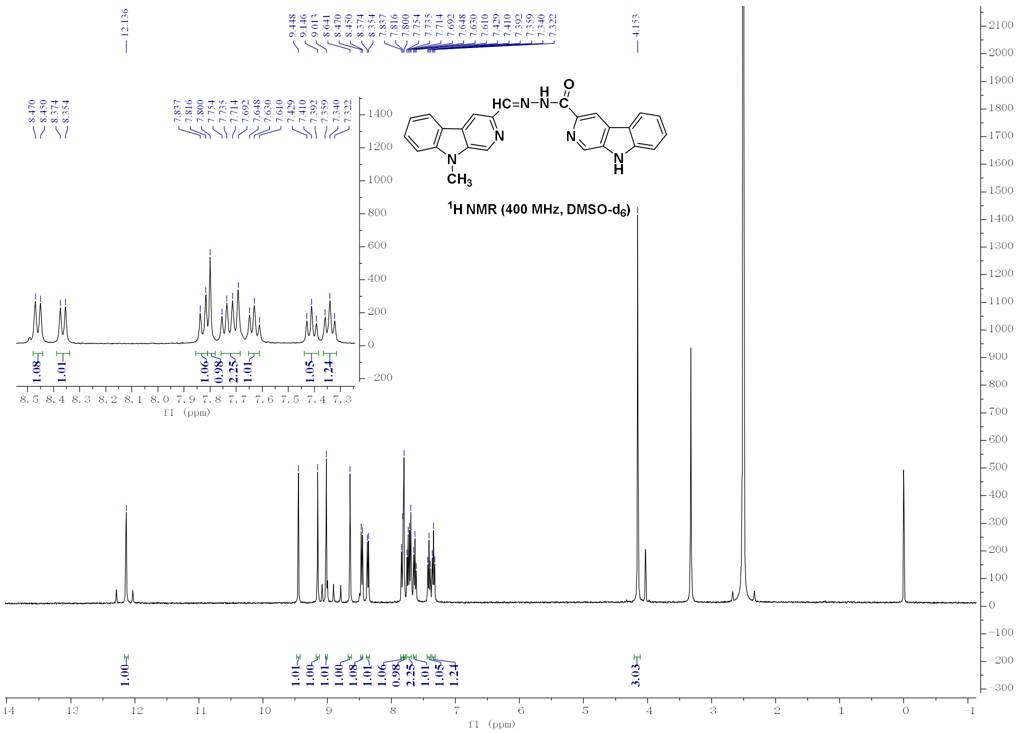

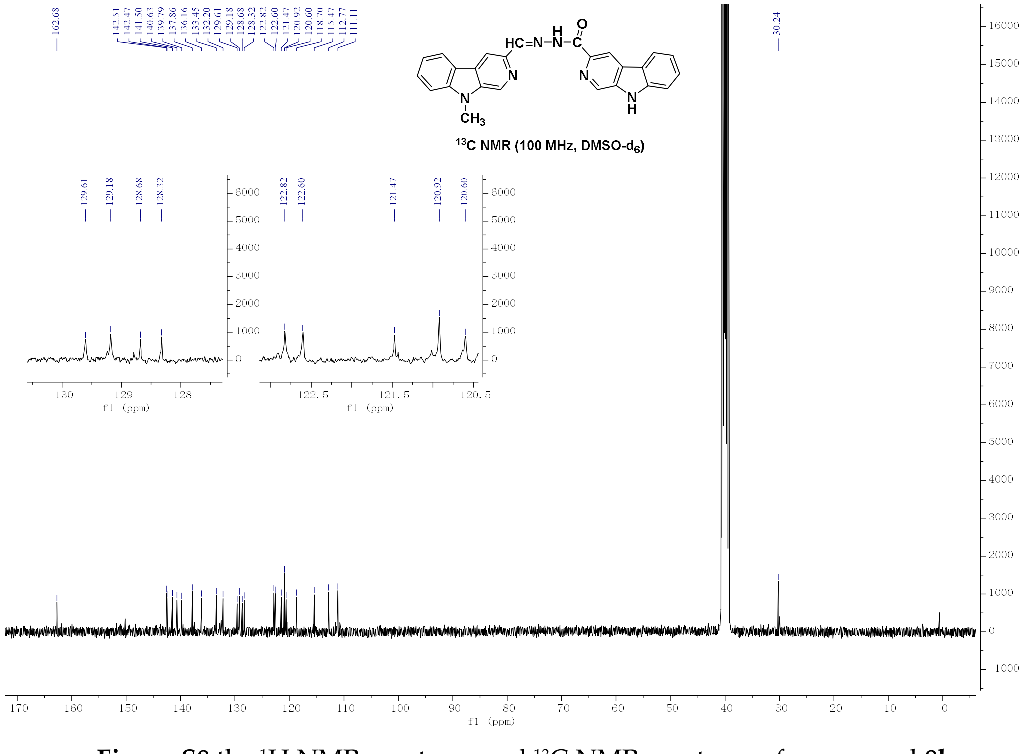

Figure S8 the ^{1}H NMR spectrum and ^{13}C NMR spectrum of compound **8h**

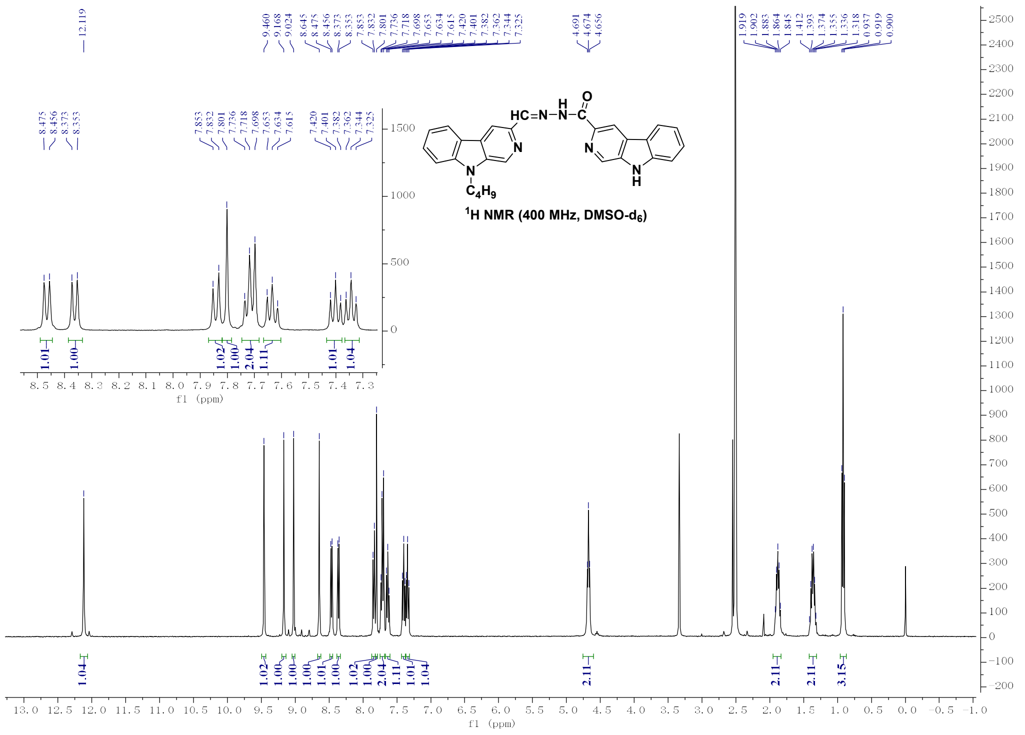

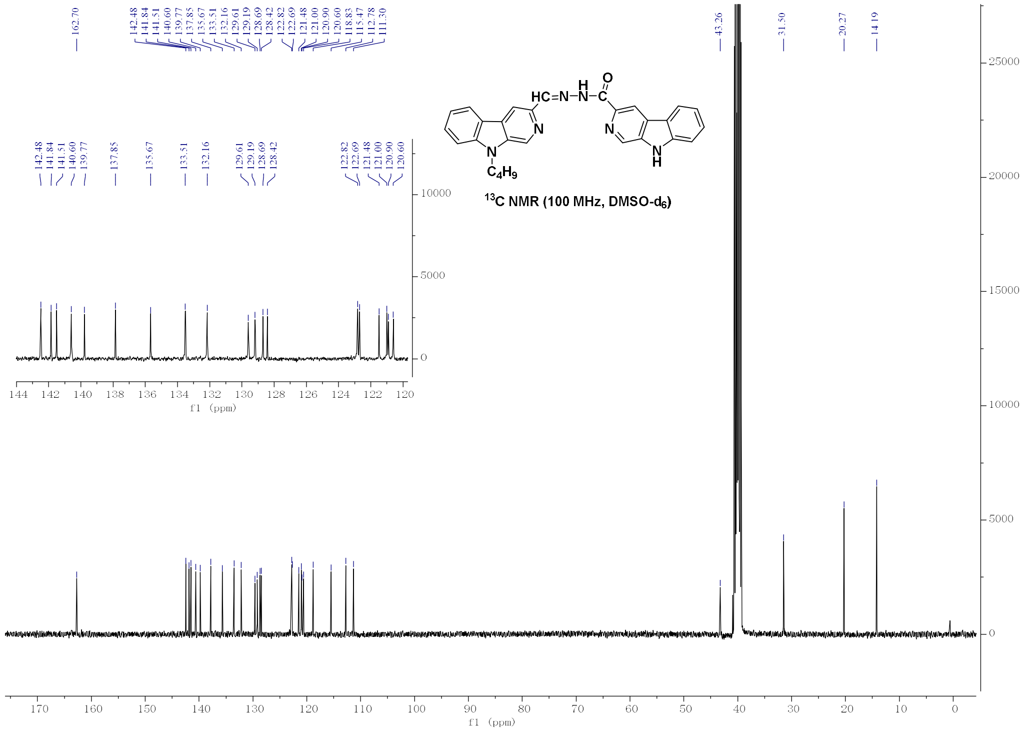

Figure S9 the ^{1}H NMR spectrum and ^{13}C NMR spectrum of compound **8i**

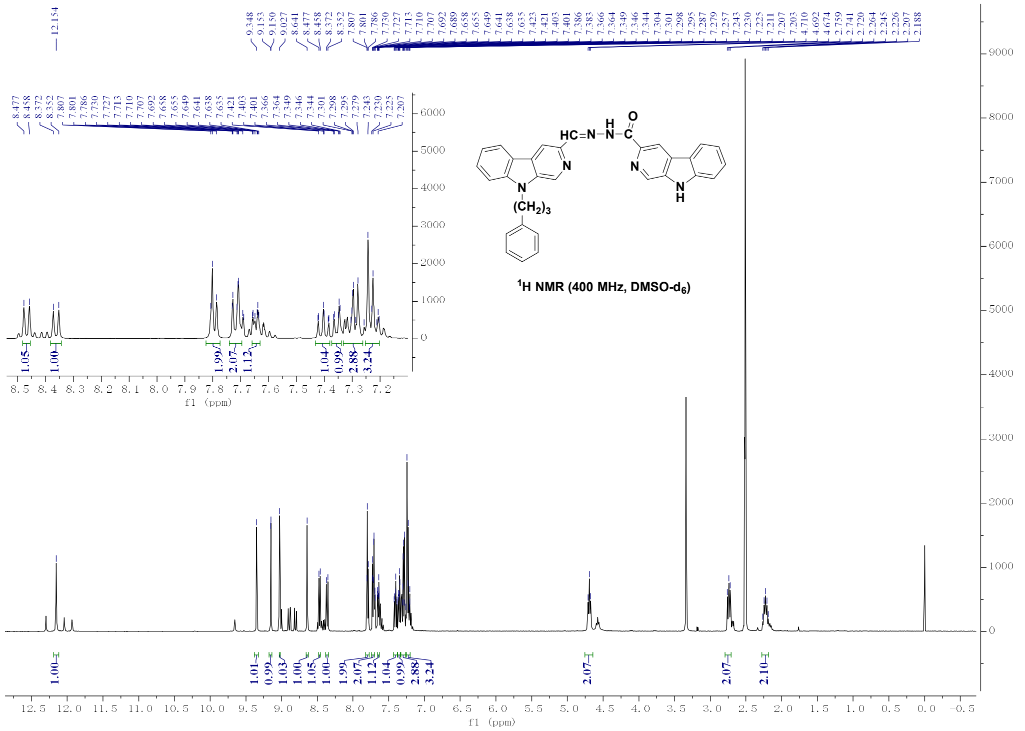

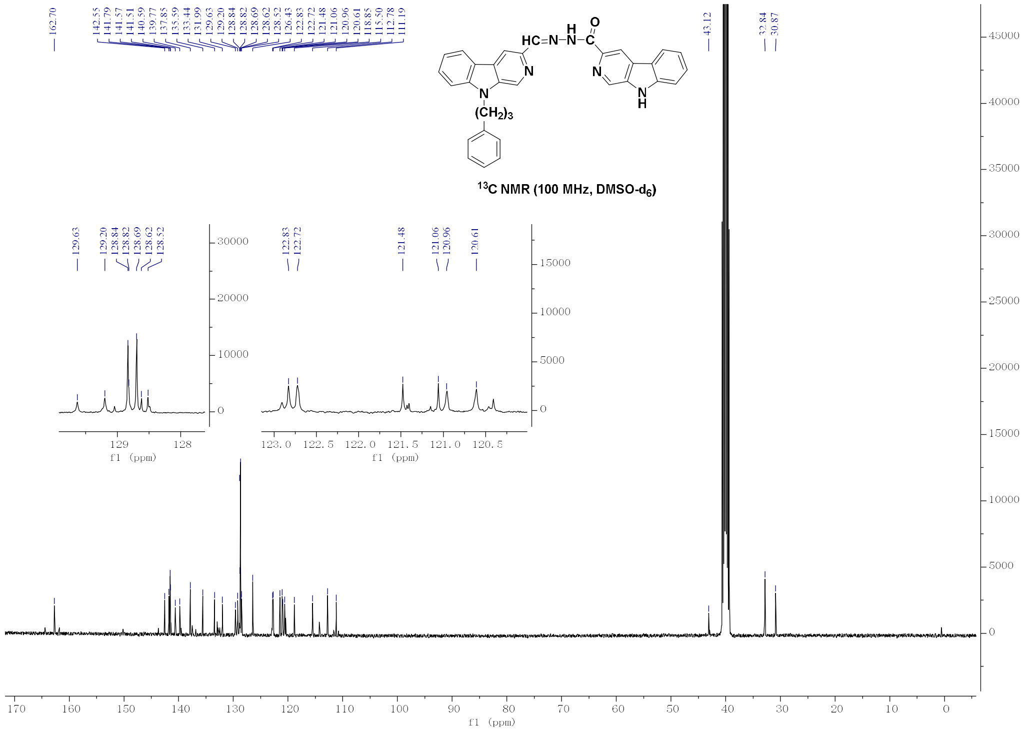

Figure S10 the ^{1}H NMR spectrum and ^{13}C NMR spectrum of compound **8j**

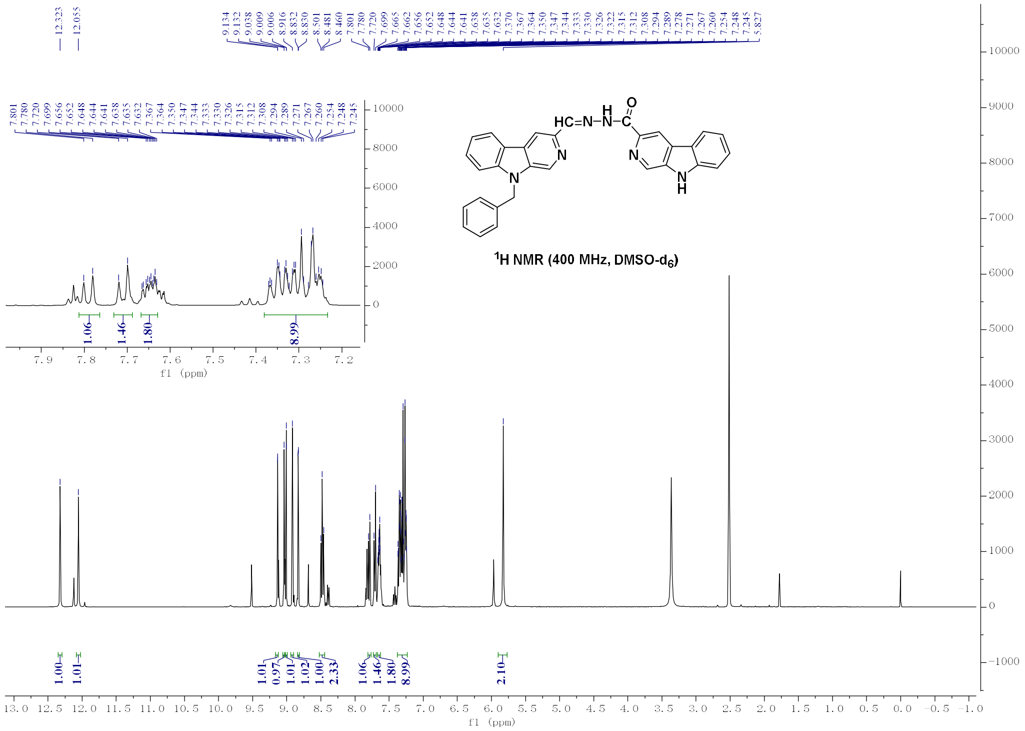

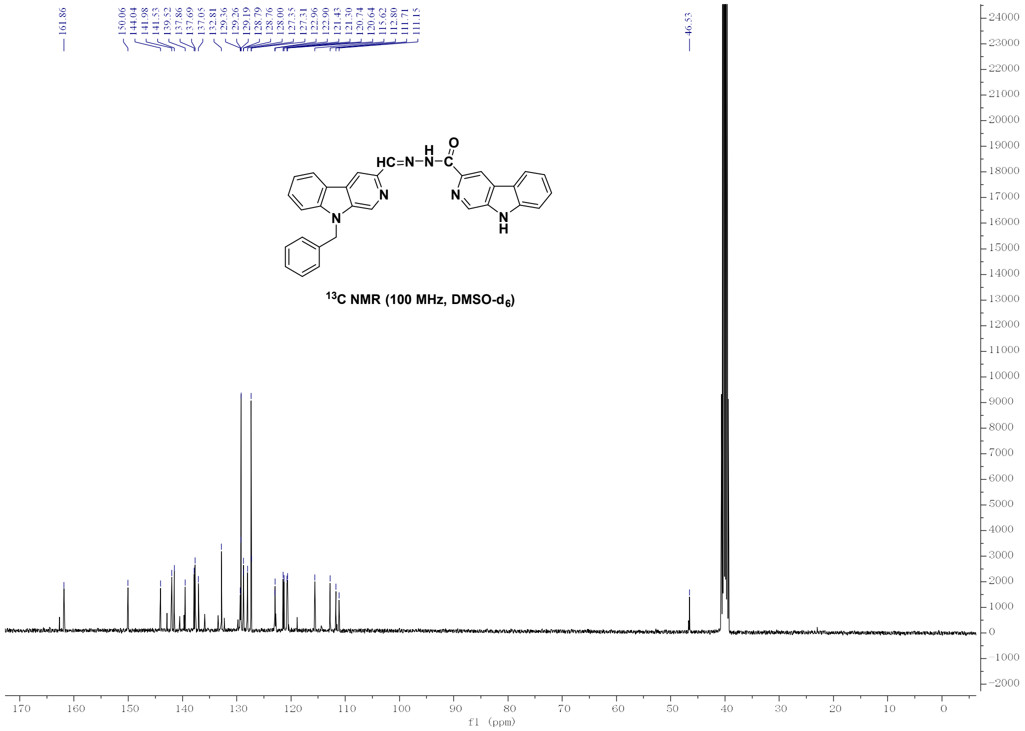

Figure S11 the ^{1}H NMR spectrum and ^{13}C NMR spectrum of compound **8k**

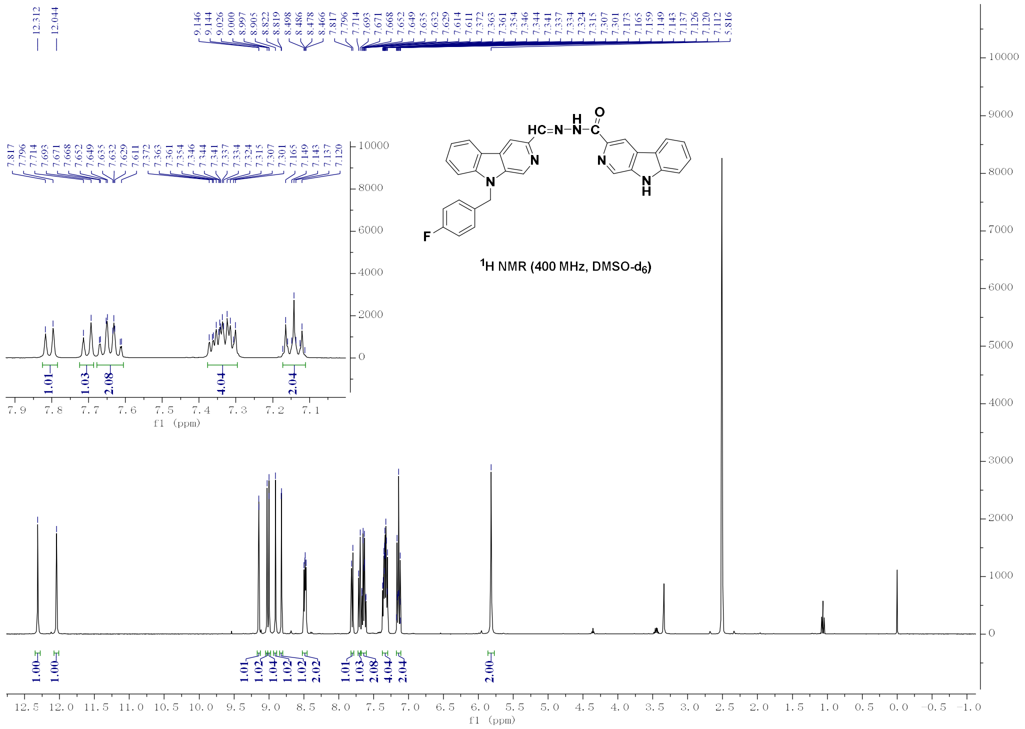

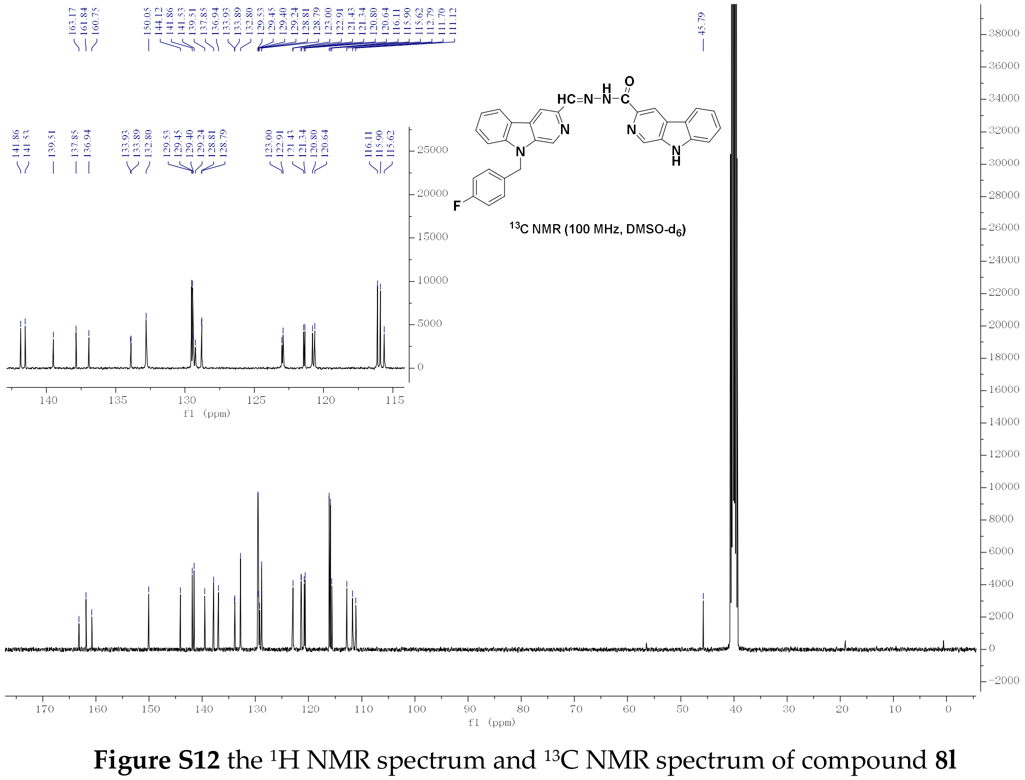

Figure S12 the ^{1}H NMR spectrum and ^{13}C NMR spectrum of compound **8l**

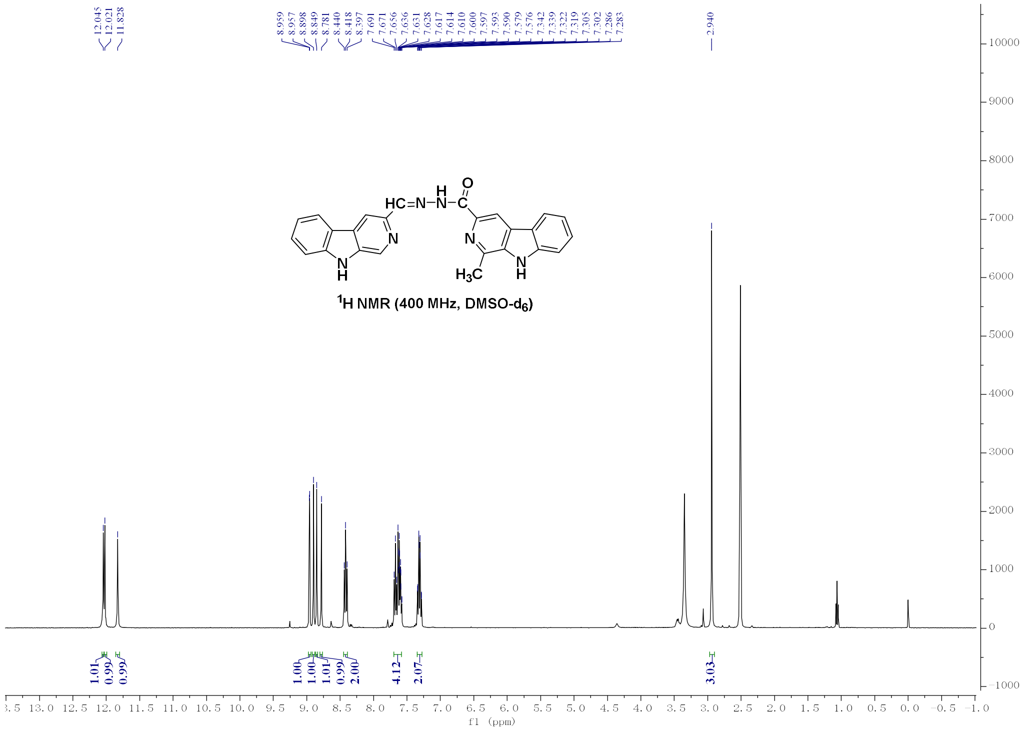

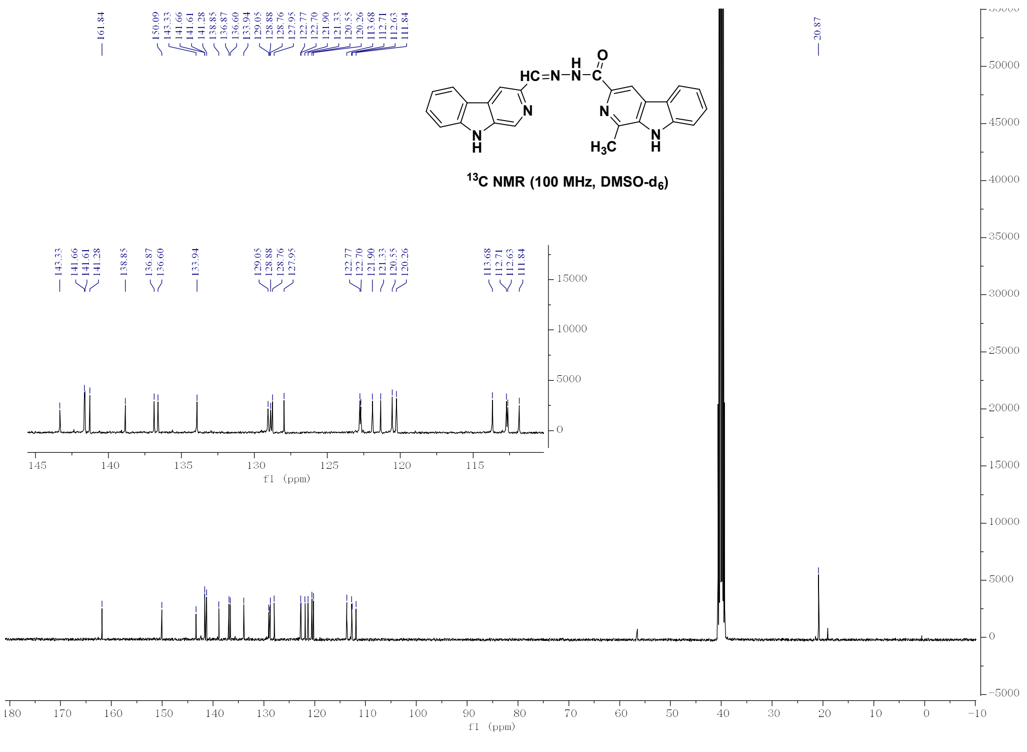

Figure S13 the ^{1}H NMR spectrum and ^{13}C NMR spectrum of compound **8m**

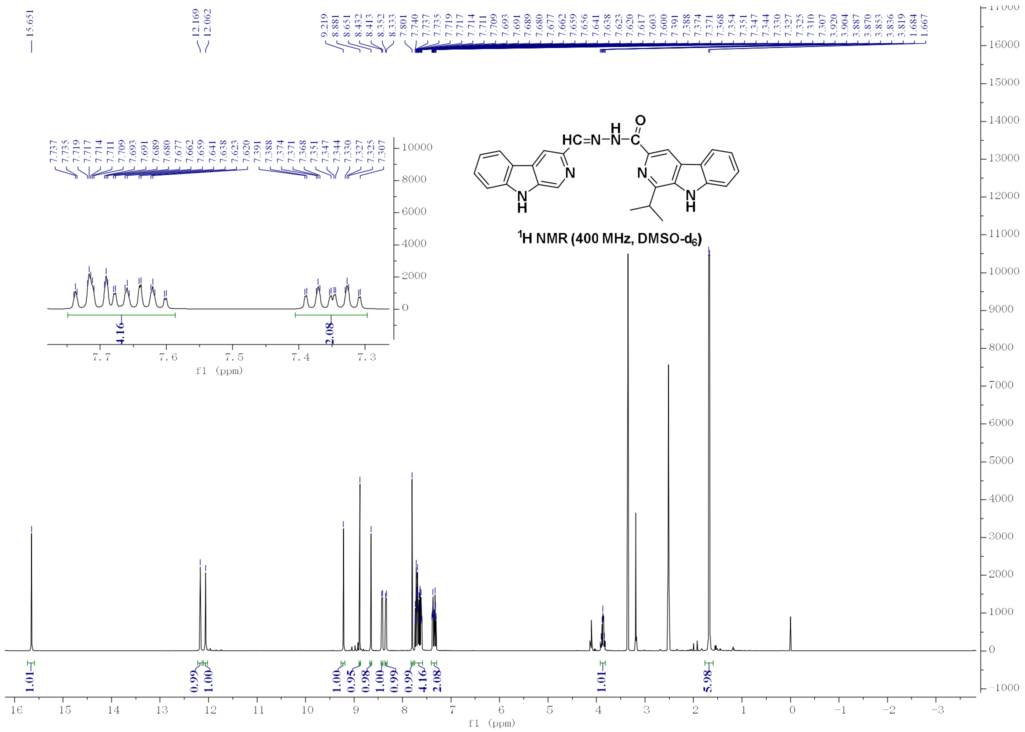

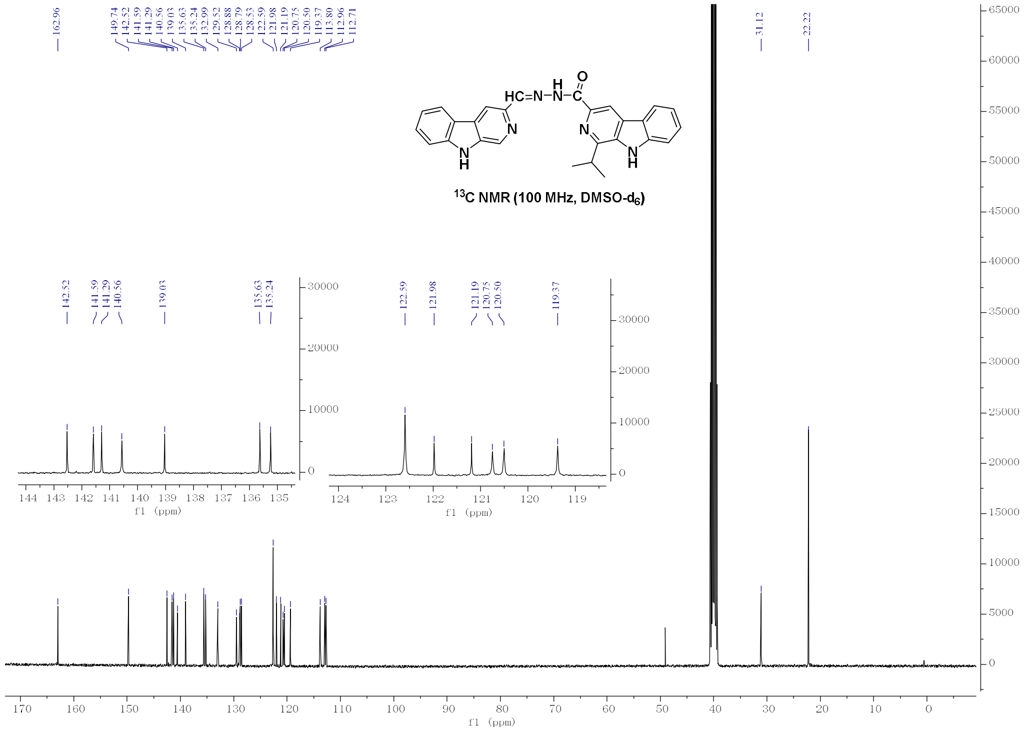

Figure S14 the ¹H NMR spectrum and ¹³C NMR spectrum of compound **8n**

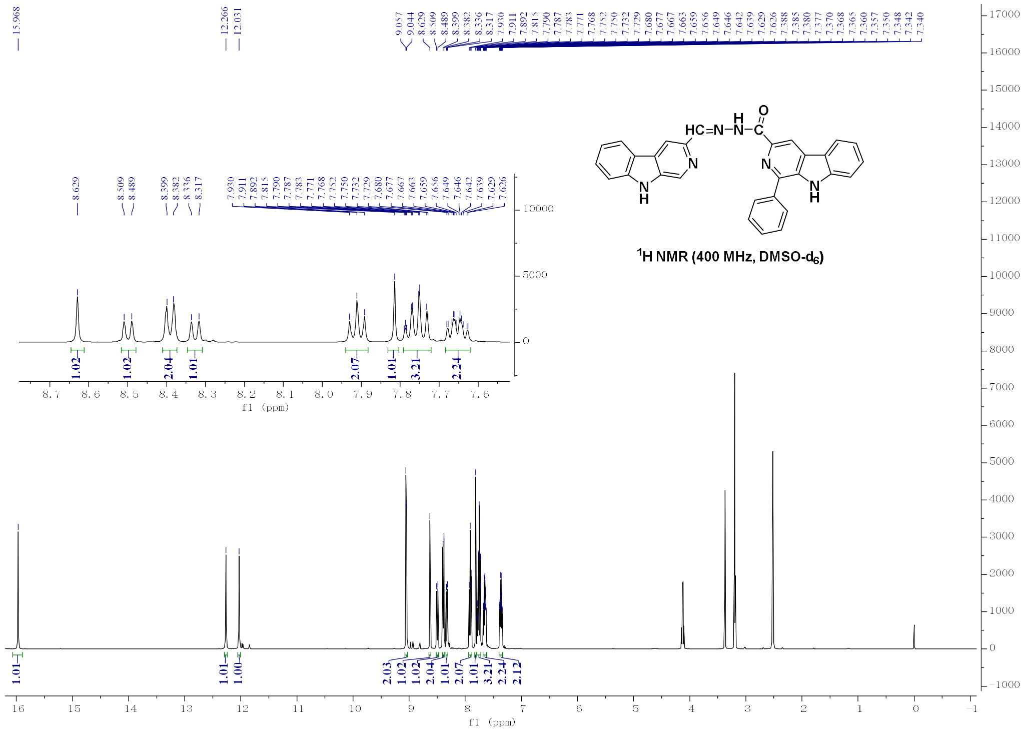

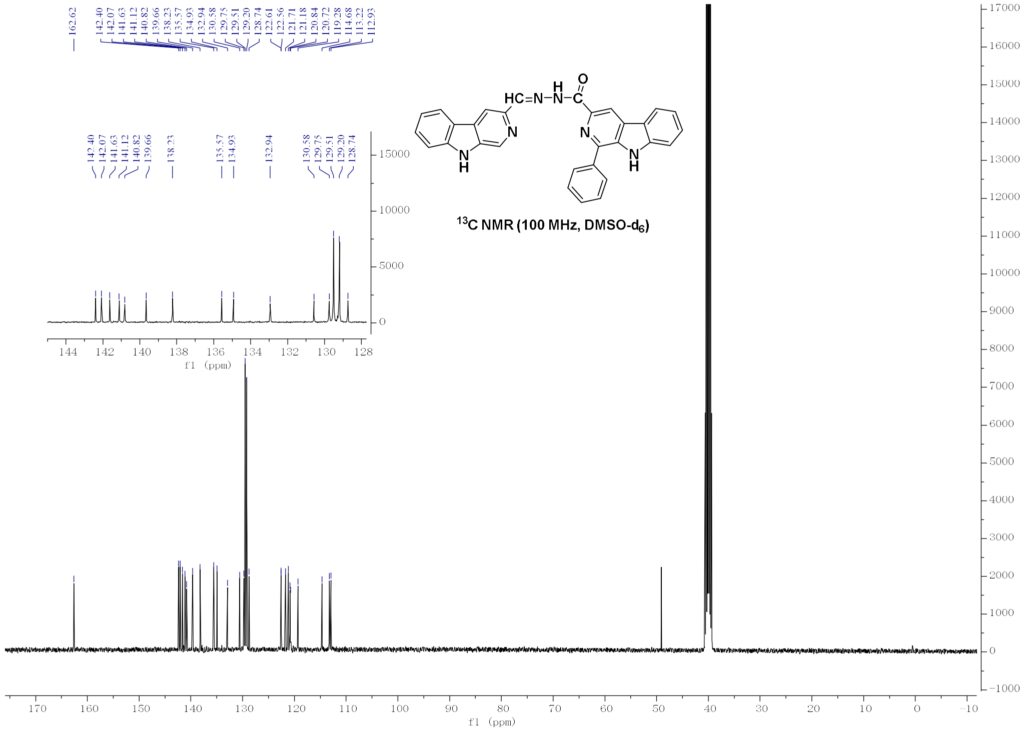

Figure S15 the ^{1}H NMR spectrum and ^{13}C NMR spectrum of compound **8o**

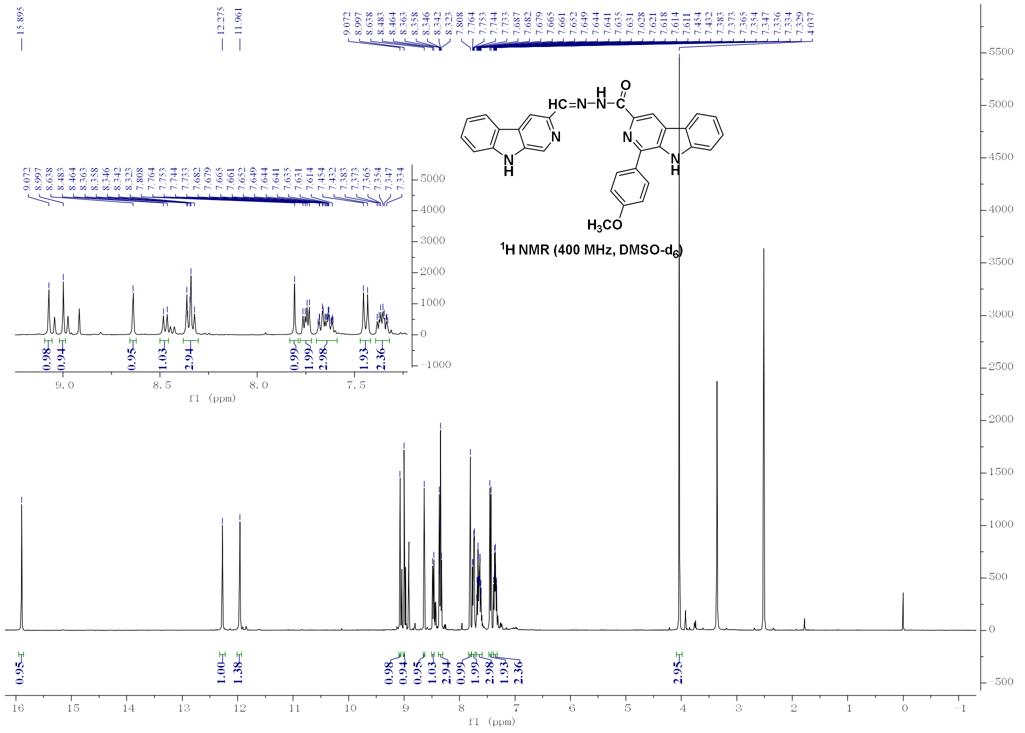

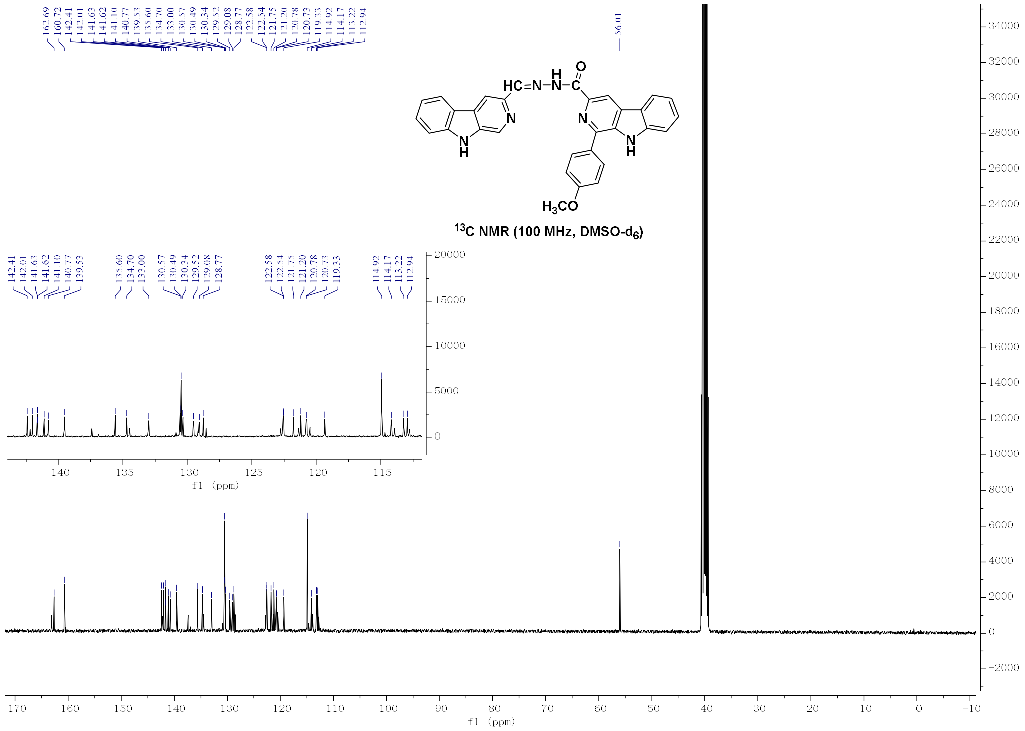

Figure S16 the ^{1}H NMR spectrum and ^{13}C NMR spectrum of compound **8p**

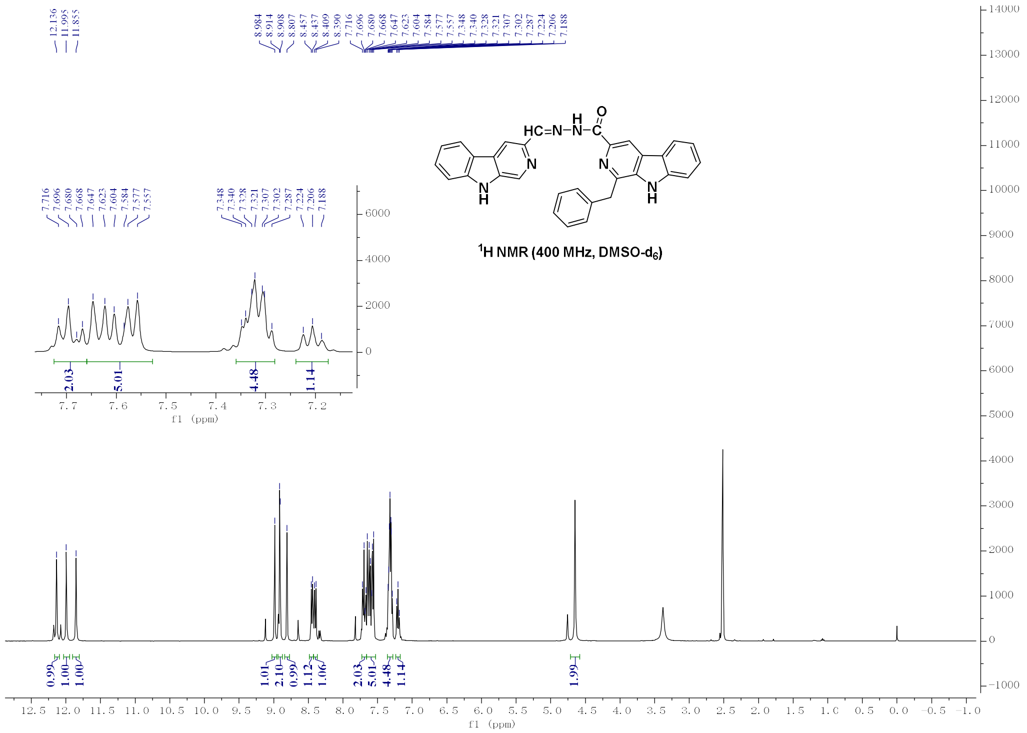

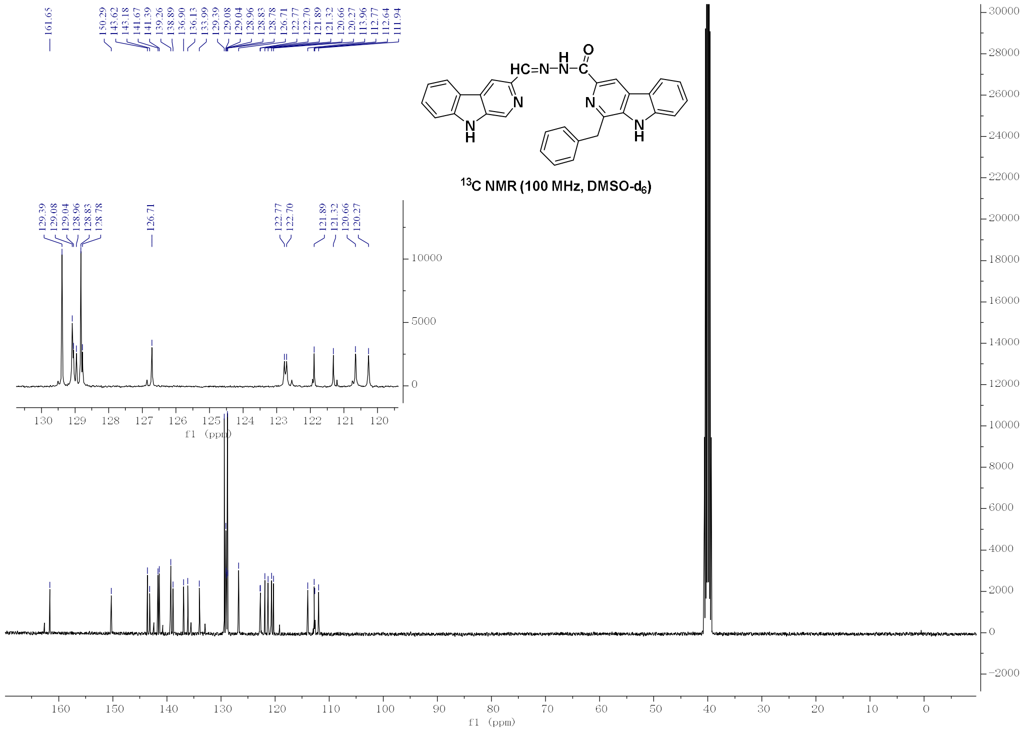

Figure S17 the ^{1}H NMR spectrum and ^{13}C NMR spectrum of compound **8q**

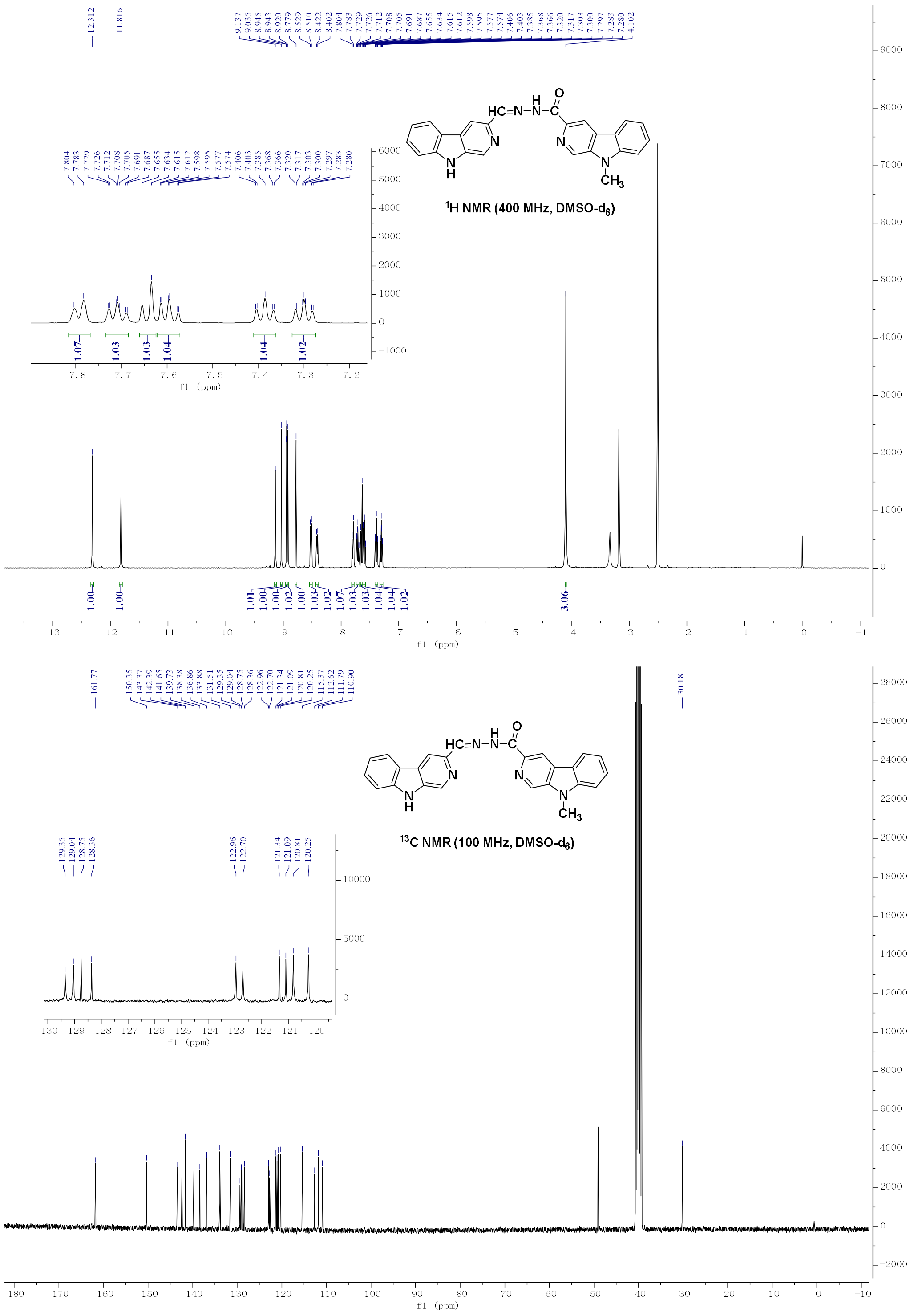

Figure S18 the ^{1}H NMR spectrum and ^{13}C NMR spectrum of compound **8r**

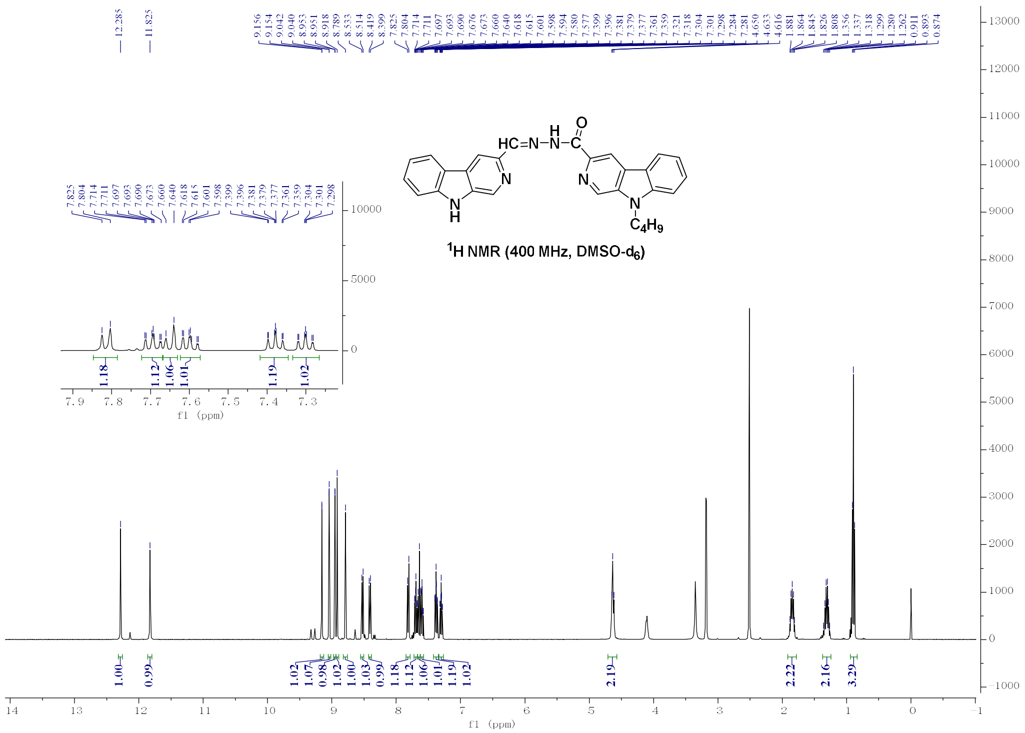

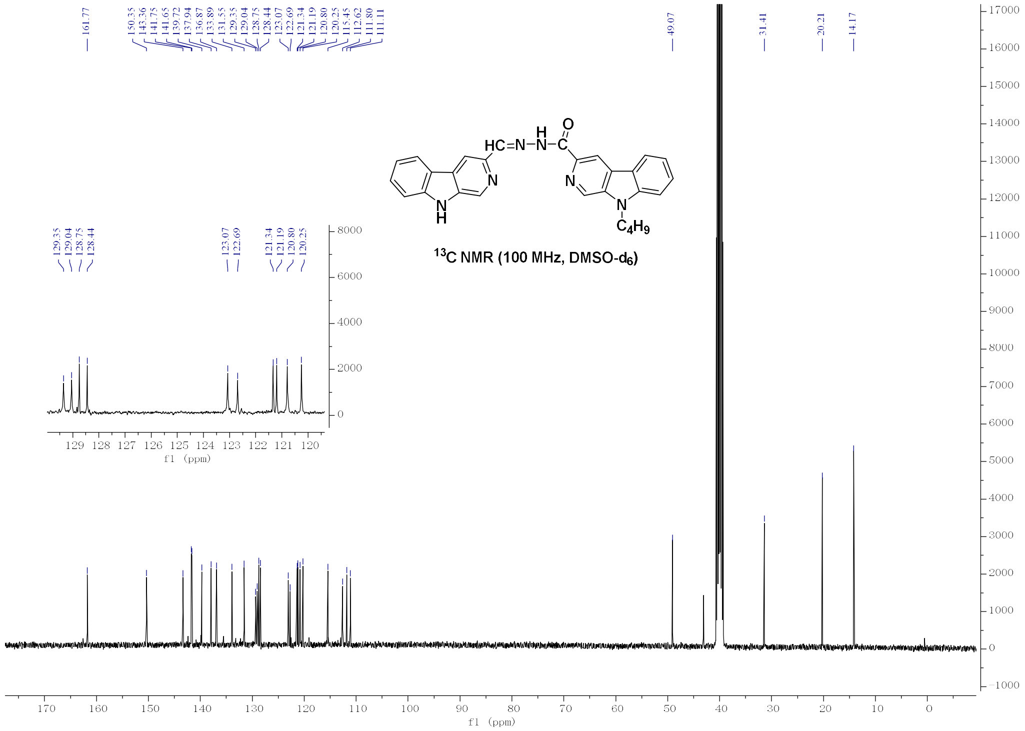

Figure S19 the ^{1}H NMR spectrum and ^{13}C NMR spectrum of compound **8s**

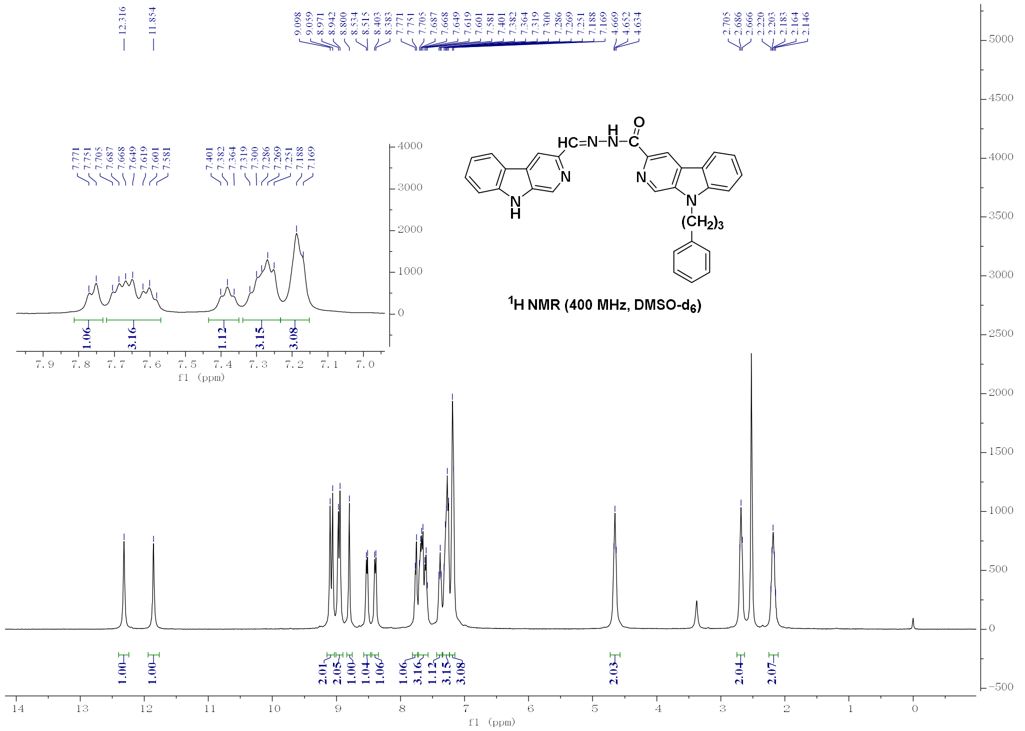

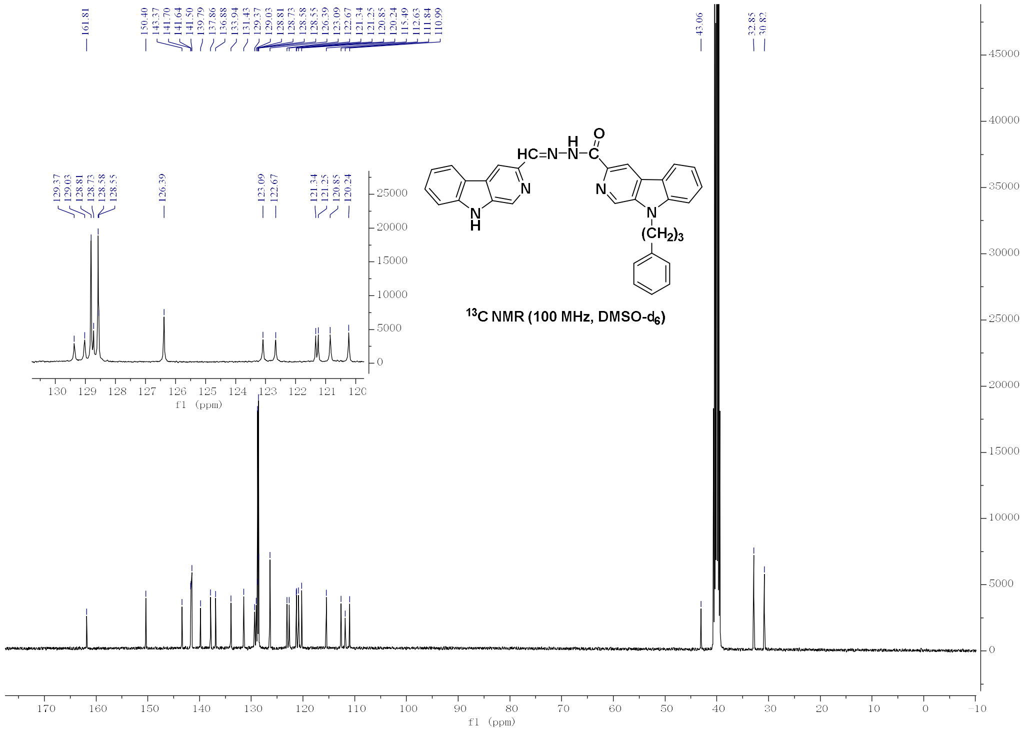

Figure S20 the ^{1}H NMR spectrum and ^{13}C NMR spectrum of compound **8t**

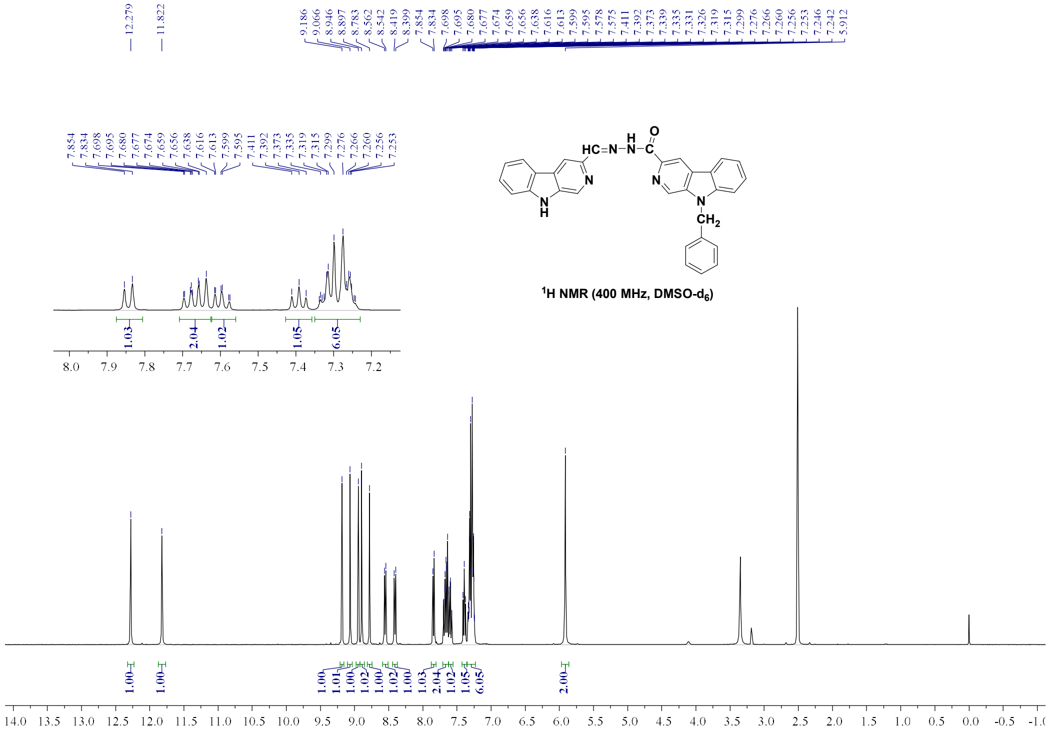

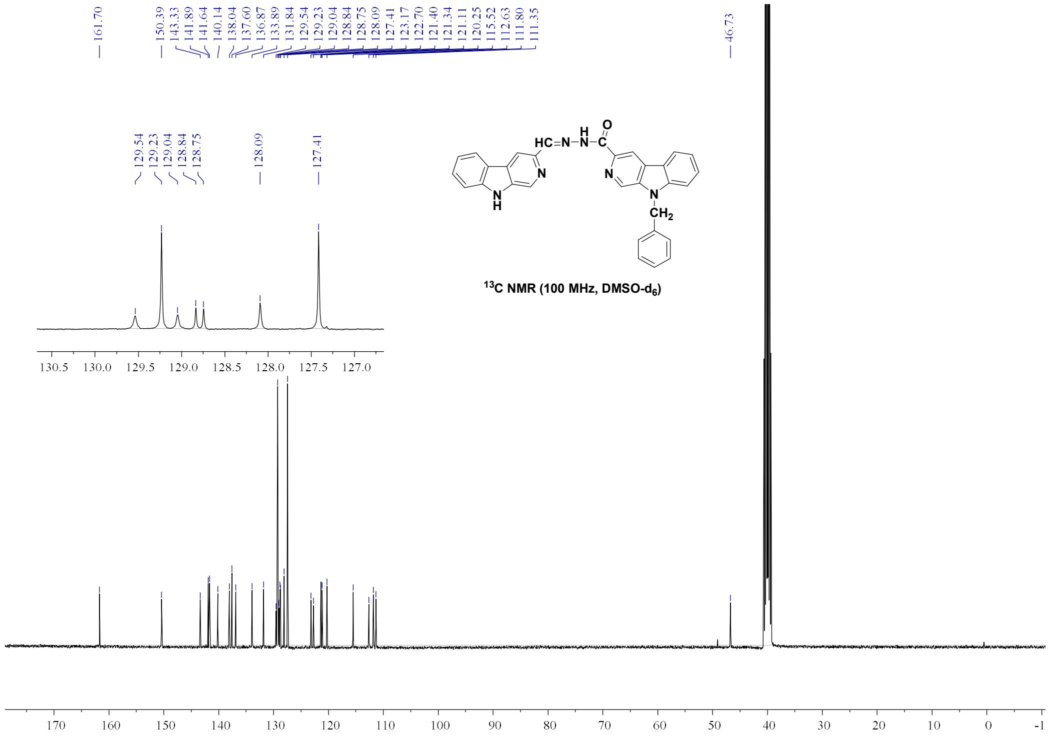

Figure S21 the ^{1}H NMR spectrum and ^{13}C NMR spectrum of compound **8u**

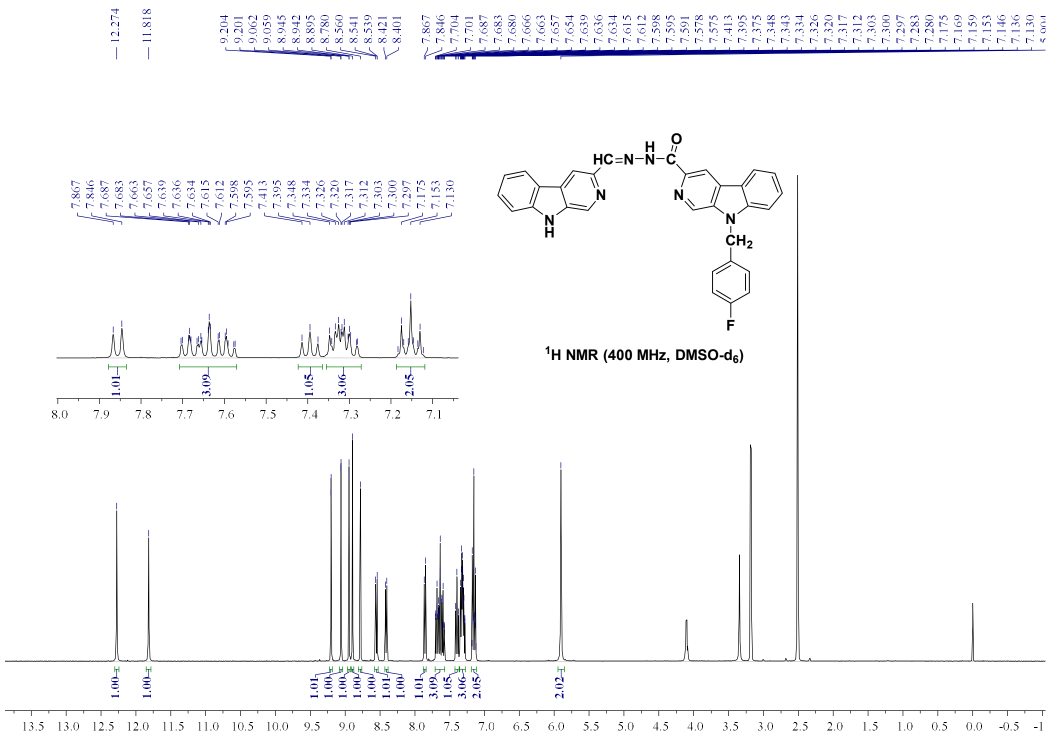

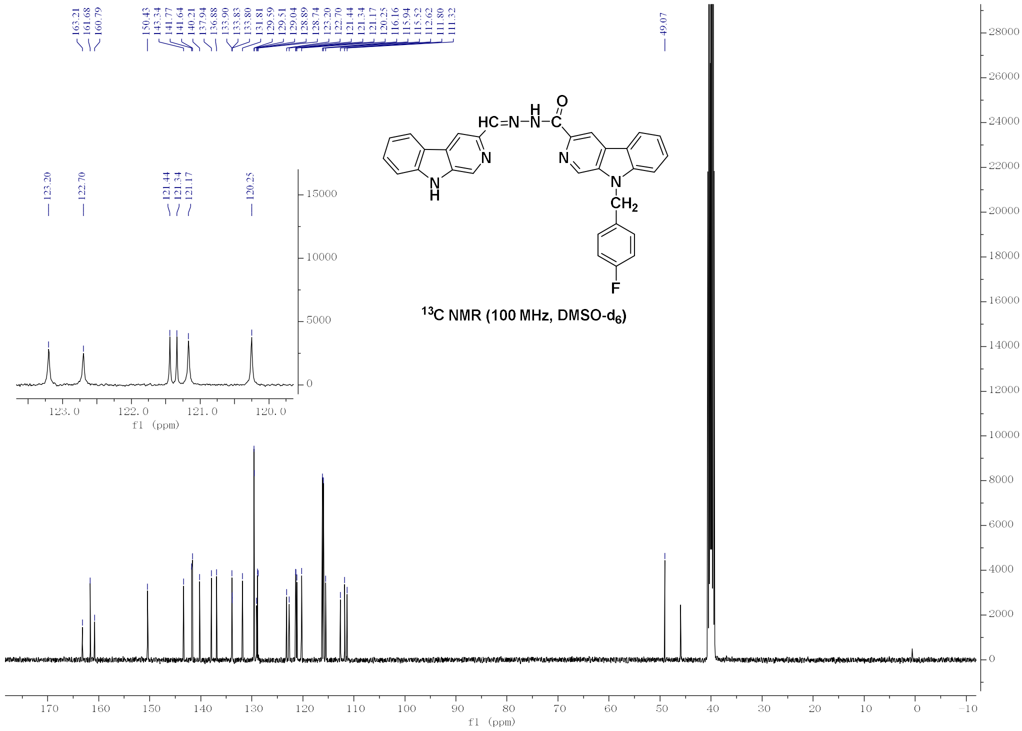

Figure S22 the ^{1}H NMR spectrum and ^{13}C NMR spectrum of compound **8v**

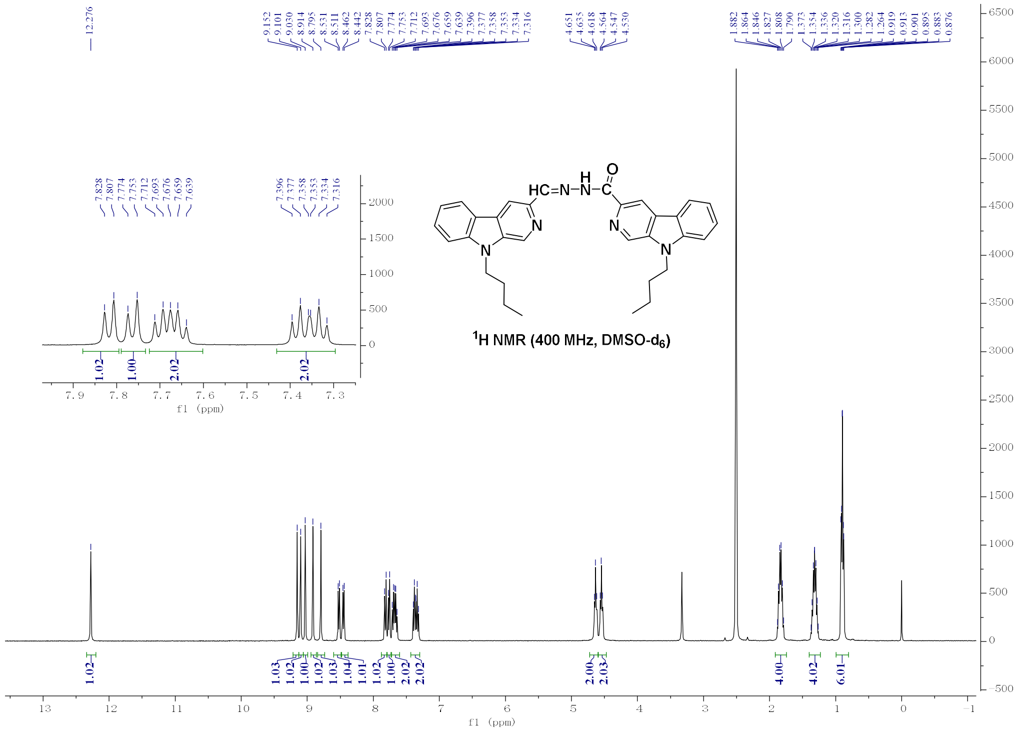

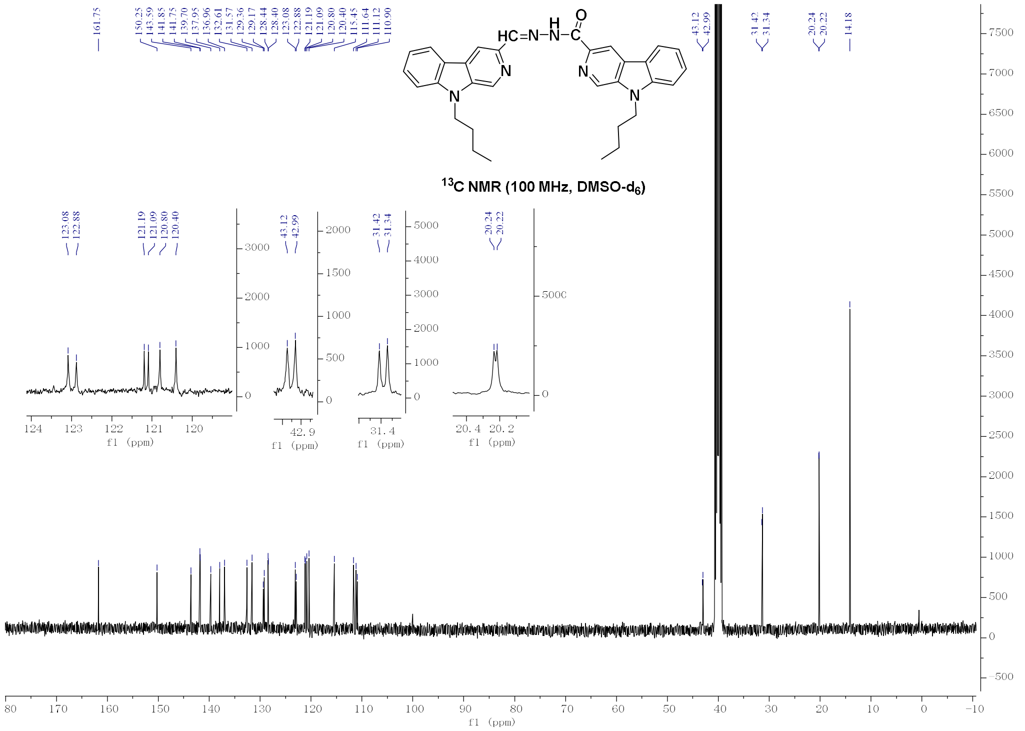

Figure S23 the ^{1}H NMR spectrum and ^{13}C NMR spectrum of compound **8w**

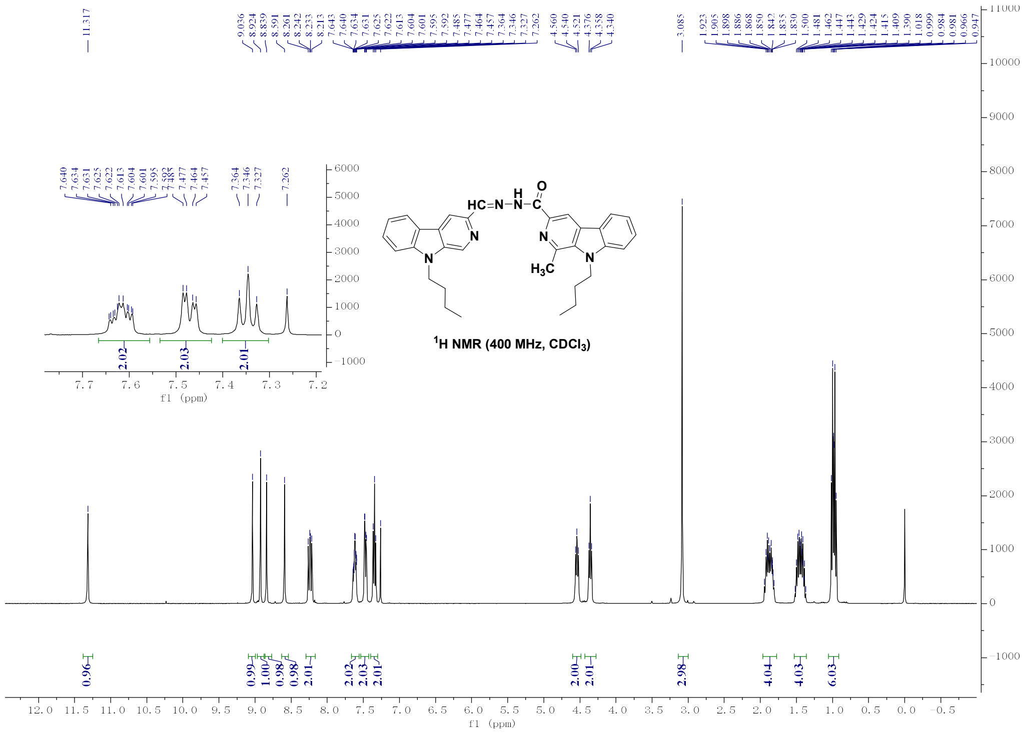

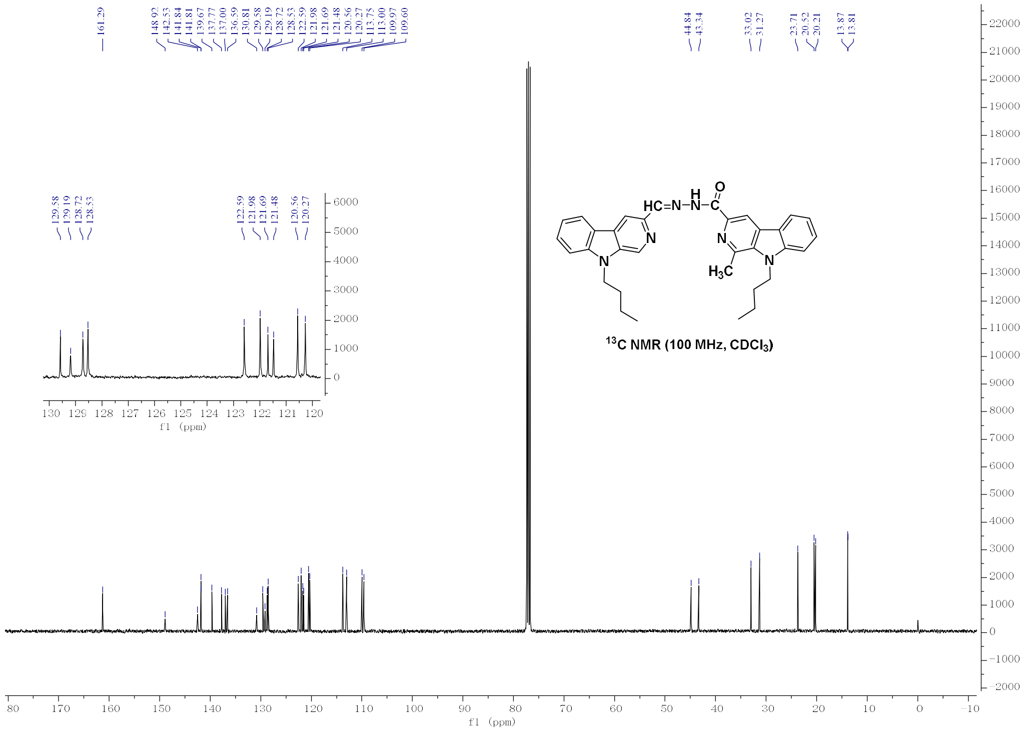

Figure S24 the ^{1}H NMR spectrum and ^{13}C NMR spectrum of compound **8x**

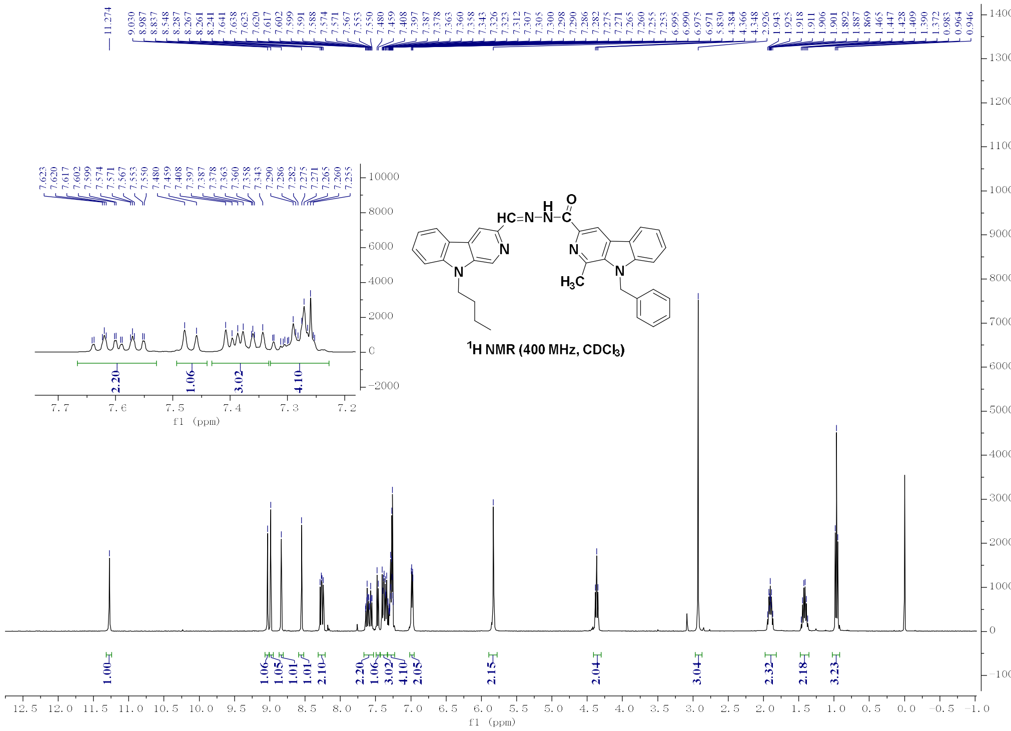

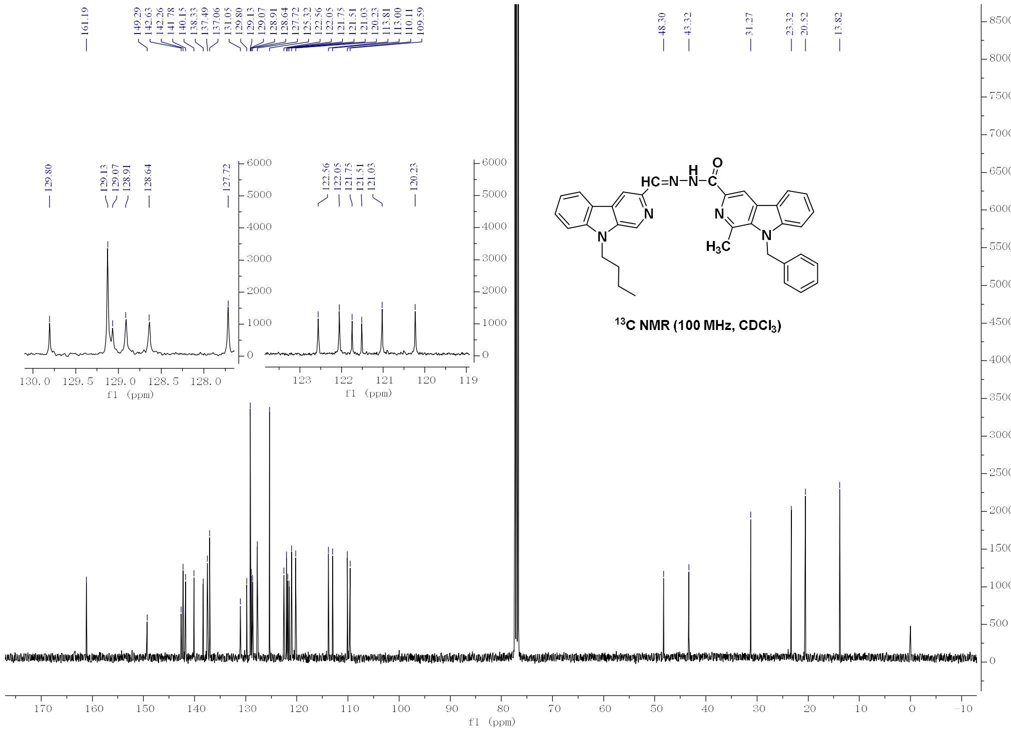

Figure S25 the ^{1}H NMR spectrum and ^{13}C NMR spectrum of compound **8y**

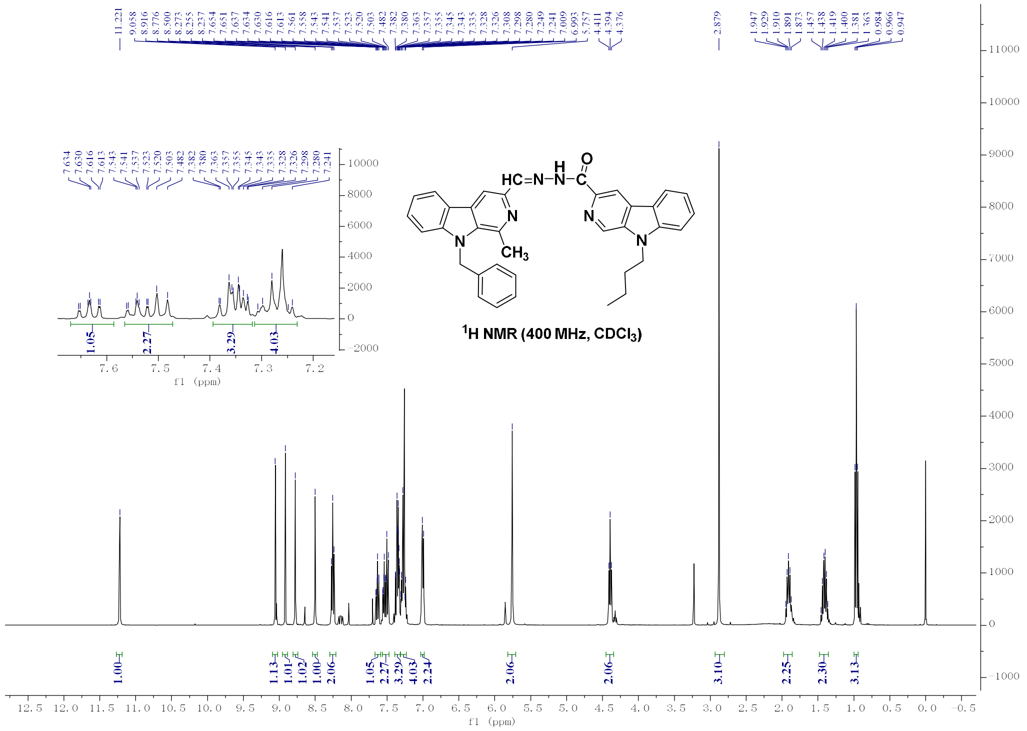

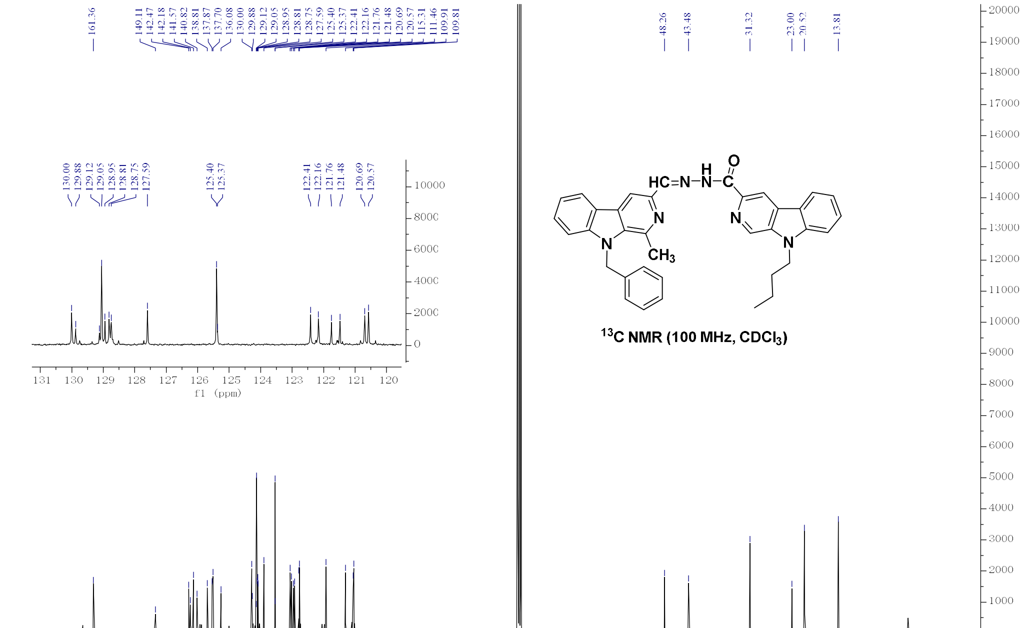

Figure S26 the ^{1}H NMR spectrum and ^{13}C NMR spectrum of compound **8z**

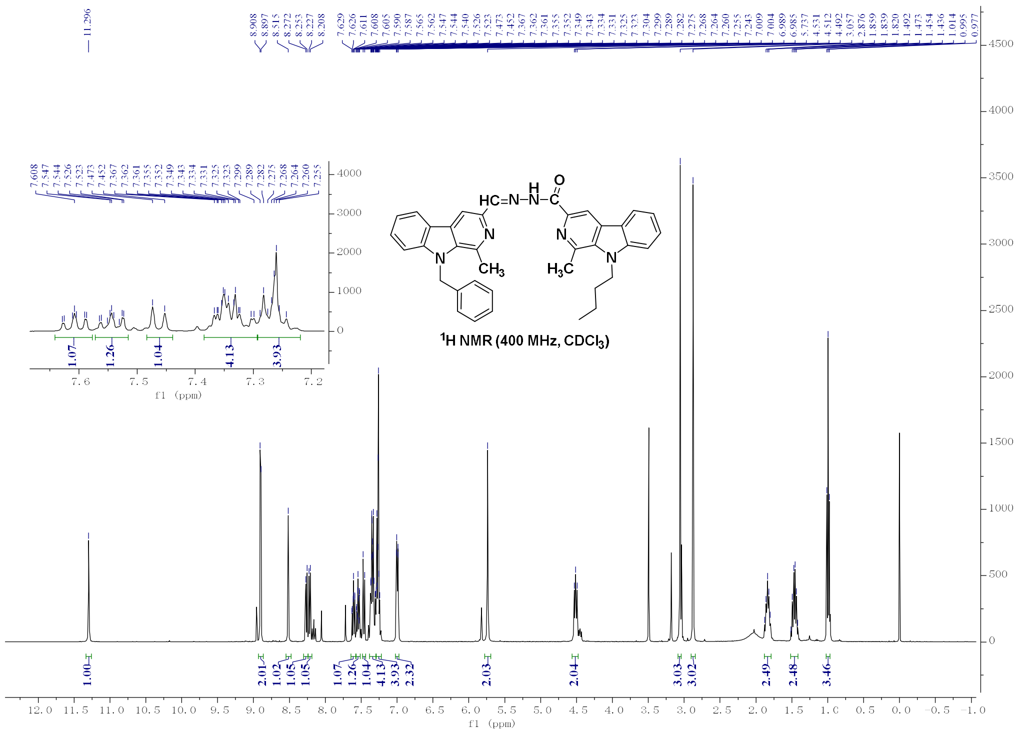

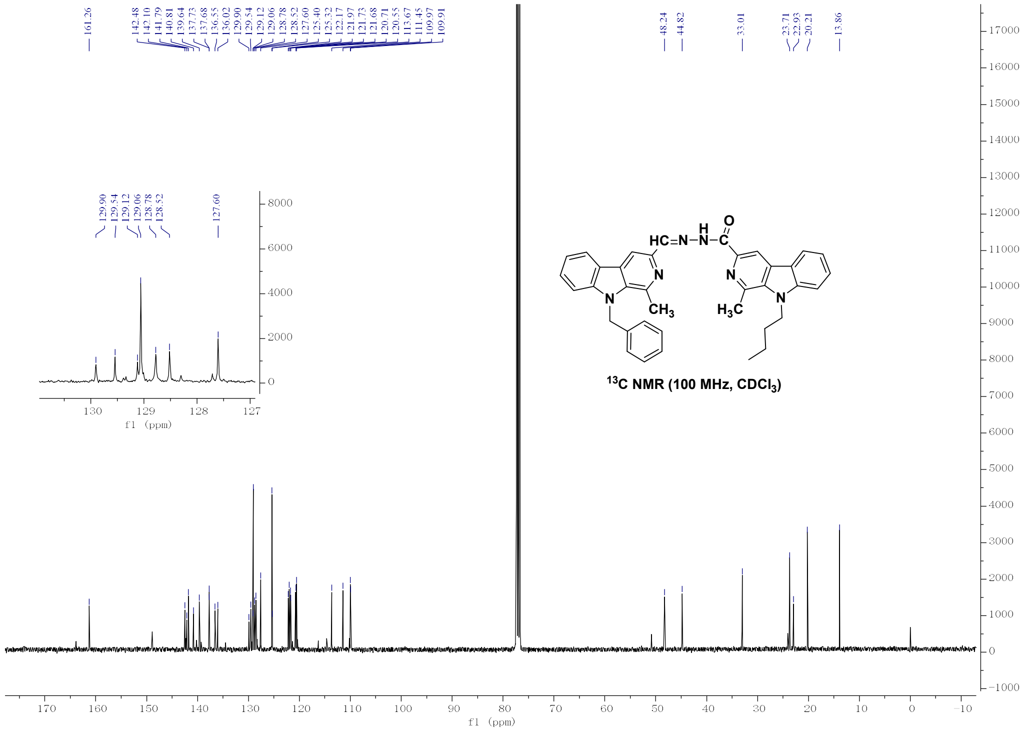

Figure S27 the ^{1}H NMR spectrum and ^{13}C NMR spectrum of compound **8aa**

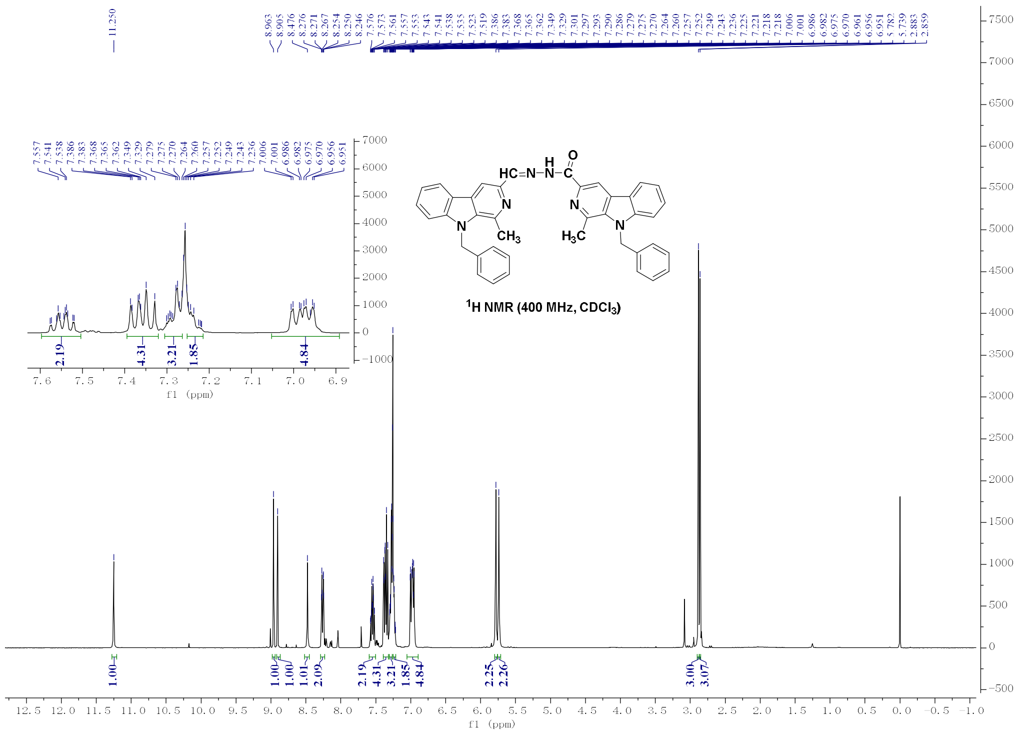

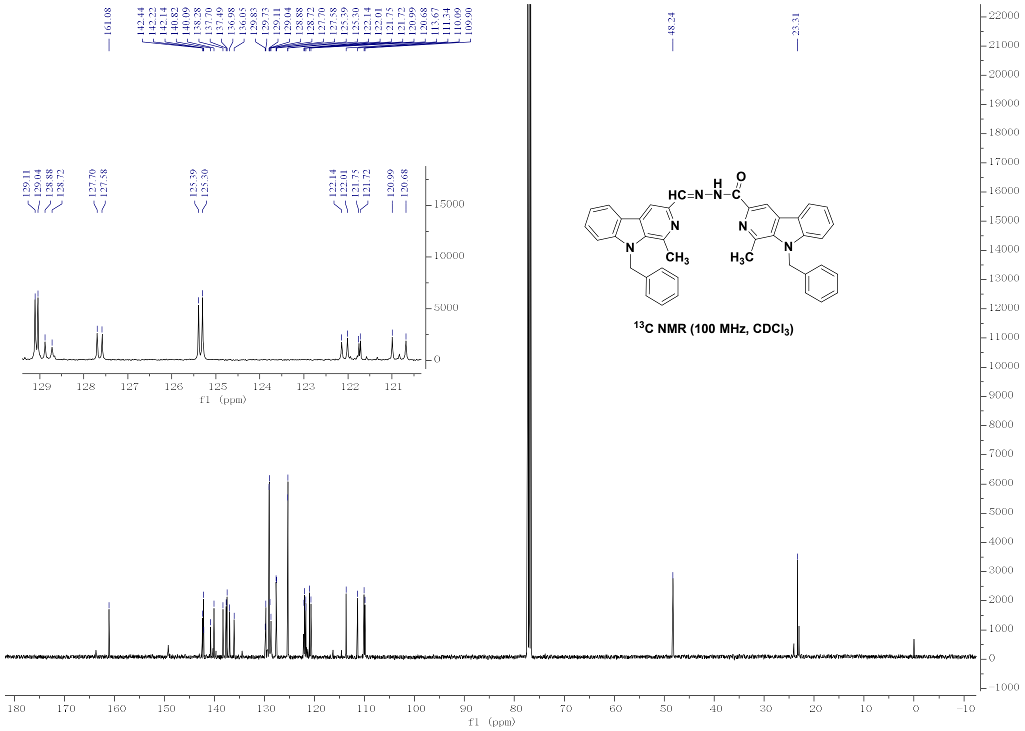

Figure S28 the ^{1}H NMR spectrum and ^{13}C NMR spectrum of compound **8ab**
